# Low-Cost 3D-Printed Binocular Indirect Ophthalmoscope

**DOI:** 10.1155/joph/5638606

**Published:** 2025-04-17

**Authors:** Brian Taeju Hwang, Weston Charles Young, Charles Campbell, Bailey Yuguan Shen

**Affiliations:** ^1^Department of Ophthalmology, Loma Linda University, 11370 Anderson St., Loma Linda, California 92354, USA; ^2^Independent Researcher, Berkeley, California, USA

**Keywords:** 3D printing, binocular indirect ophthalmoscope, wireless spectacle style

## Abstract

**Background:** Currently available binocular indirect ophthalmoscopes (BIOs) are large and expensive. We sought to create a compact, low-cost 3D-printed BIO.

**Methods:** The BIO was made with off-the-shelf electronics and optical components, computer-aided design (CAD), and a consumer-grade 3D printer. Ocular light safety was tested with a spectrometer.

**Results:** The component cost of the 3D-printed BIO was $182.26. The wireless, spectacle-style BIO weighed 120 g and was more compact than commercially available BIO's, with the advantage of a battery incorporated into the frame. The BIO met the International Organization for Standardization's standards for indirect ophthalmoscopes, as well as the American National Standards Institute's Group 1 light hazard protection standards for ophthalmic instruments.

**Conclusions:** It is possible to produce a high-quality, low-cost BIO using CAD and 3D printing. Such a BIO may be useful in both resource-rich and resource-limited settings.

## 1. Introduction

Binocular indirect ophthalmoscopes (BIOs) are commonly used by general ophthalmologists and retinal specialists in the diagnosis and management of retinal diseases. However, commercially available BIOs tend to be large and expensive, possibly limiting their use in resource-limited settings. Portable, low-cost indirect ophthalmoscopes have been described, but these are either monocular or not available [1–3].

3D printing and computer-aided design (CAD) have become more affordable and accessible to consumers over the last decade, which has led to their increased use in the field of ophthalmology [4]. Luca et al. used CAD and 3D printing to create personal protective equipment for ophthalmologists wearing BIOs [5]. Here, we describe a compact, low-cost, 3D-printed BIO, capable of being produced with a consumer-grade 3D printer and off-the-shelf optical and electronic components. A preprint of this manuscript has previously been published [6].

## 2. Materials and Methods

All 3D printed parts were designed using the CAD program, Autodesk Fusion 360 (Version 16, San Francisco, California, United States of America). OrcaSlicer (Version 2.0.0, https://github.com/SoftFever/OrcaSlicer) was used to slice the files made in Fusion 360 to prepare for printing. OrcaSlicer was chosen over Bambu Slicer because of OrcaSlicer's calibration and filament configuration options. The Bambu Lab X1-Carbon 3D printer (https://bambulab.com/en-us, purchased in 2024, manufacturer's suggested retail price of $1449 at the time of writing) was used to print the components. The BIO was 3D printed from acrylonitrile styrene acrylate (optics housing, frame, and temples), transparent polyethylene terephthalate glycol (window for charging light), and thermoplastic polyurethane (eyepieces, light-emitting diode [LED] holder, charge cover, and washer for nosepiece).

### 2.1. Ocular Light Safety

Ocular light safety measurements were taken according to American National Standards Institute Z80.36-2021 [7]. Retinal radiant exposure was calculated from our method previously described [8]. We evaluated the irradiances created when the indirect ophthalmoscope was used at maximum brightness and maximum aperture with a 20D handheld condensing lens. The diameter of the light beam in the anterior segment (for standard 5.4.1.5) was found to be 1.5 mm. The distance between the anterior surface of the central mirrors and the retinal image plane was found to be 45 cm (focal length of the +2D eyepiece lenses (50 cm) minus the optical path length between the eyepiece lenses and the anterior surface of the central mirrors (5 cm)). Assuming an emmetropic eye with the distance from the posterior nodal point to the retina being 17 mm, we multiplied the irradiance of the indirect at the retinal image plane by 3460/(power of handheld condensing lens)^2^ to calculate the irradiance at the retinal surface (used for standards 5.4.1.3 and 5.4.1.6) [7, 8]. A Black-Comet spectroradiometer and CR2 receptor (purchased in 2020, StellarNet, Tampa, Florida, United States of America) were used for irradiance measurements in 5 nm increments.

## 3. Results

**Figure 1:**
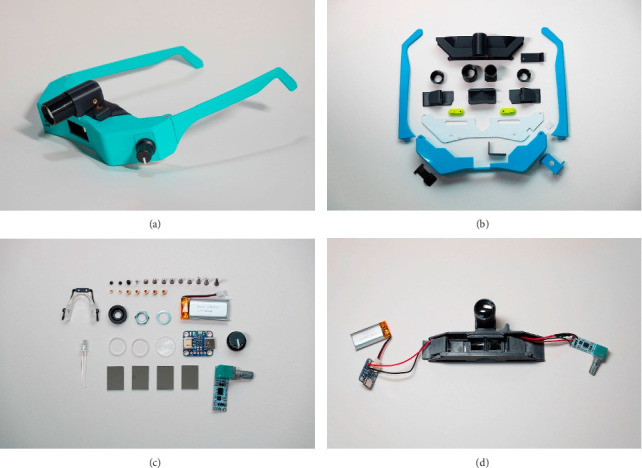
3D-printed binocular indirect ophthalmoscope. Completed indirect without glasses strap (a). 3D-printed components (b). Hardware, electronic, and optical components (c). Assembled circuit and optical system (d).

**Figure 2:**
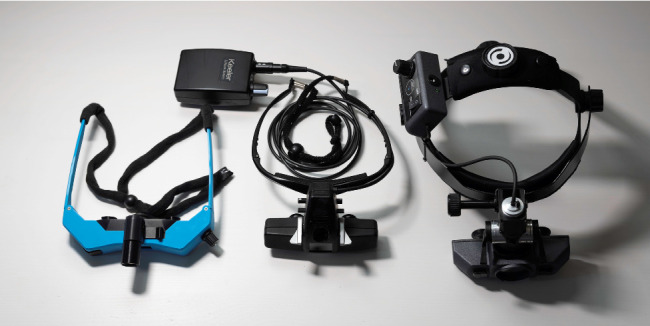
Size comparison of the 3D-printed binocular indirect ophthalmoscope (left), Keeler Spectra Iris (middle), and generic headband-mounted ophthalmoscope (right).

Figure 1 shows the complete 3D-printed BIO as well as its components. The 3D-printed BIO resembles a commercially available spectacle-mounted BIO (such as the Keeler Spectra Iris, Heine Sigma 250, or Scan Optics SO-2200), with the exception that the battery is contained within the body of the BIO. The indirect weighed 120 g and was more compact than commercially available indirects (see Figure 2). The total cost of all components needed to create one indirect was $182.26 (see Supporting Table). The total cost of the tools needed to build the BIO (including the 3D printer) was $1707.20 (Supporting Table). A parts list (Supporting Table), standard tesselation language files for the 3D printed parts, step-by-step build guide and video are available as Supporting Information. An ophthalmologist with some electronics experience can print the parts in 4 h and 38 min and assemble our indirect from its components in about 2–4 h using a soldering iron and small hand tools.

**Figure 3:**
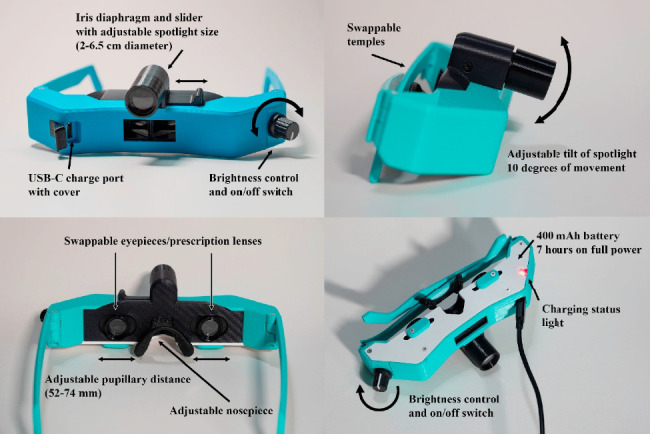
Features of the 3D-printed binocular indirect ophthalmoscope.

Figure 3 displays the features of the 3D-printed BIO. The BIO met the International Organization for Standardization's standards for indirect ophthalmoscopes [9]. The lighting system consists of a 5 mm warm white LED, iris diaphragm, and plano-convex lens, all of which produce a collimated circular spotlight that can be tilted from 0 to 10° down with respect to the optical axis (see Video). The iris diaphragm can be used to change the spotlight size from 2 to 6.5 cm (measured at 50 cm away from the BIO). The intensity of the light is controlled by a pulse-width modulation board and dimmer knob. Power is provided by a 400 mAh lithium battery contained within the frame, which is charged via a USB-C port. The battery lasts approximately 7 h at maximum brightness.

The optical system consists of eyepieces that can hold different power lenses (e.g., +2D lenses for emmetropic users), mirrors for the right and left eyes that can be slid to accommodate different interpupillary distances over a range of 52–74 mm, and central mirrors that can slide anteriorly and posteriorly to adjust the amount of stereopsis. With +2D lenses in the eyepieces, the magnification of the retinal image for the user is 1.5x. The optical system is similar to commercially available indirects, and the quality of the retinal image is comparable to commercially available indirects based on the authors' testing. We found that our indirect has a shorter vertex distance than commercially available spectacle-style indirects, leading to a slightly larger field of view with our indirect. The temples were made in different sizes and shapes to accommodate different face shapes and head sizes. An adjustable nosepiece on the frame was used to accommodate different nose shapes and bridge heights.

### 3.1. Ocular Light Safety

The 3D-printed BIO met the American National Standards Institute Group 1 standards for ophthalmic instruments [7]. The irradiances of the 3D-printed BIO for wavelengths less than 400 nm or greater than 800 nm were found to be negligible. For the retinal photochemical aphakic light hazard (5.4.1.3), the weighted retinal irradiance of the 3D-printed indirect was 330 μW/cm^2^, which was below the Group 1 limit of 440 μW/cm^2^. The unweighted anterior segment visible and infrared radiation irradiance of the 3D-printed BIO was 0.13 W/cm^2^, which was below the Group 1 limit of 4 W/cm^2^ (5.4.1.5). The weighted retinal visible and infrared radiation thermal irradiance of the 3D-printed BIO was 0.0053 W/cm^2^, which was below the Group 1 limit of 0.7 W/cm^2^ (5.4.1.6).

## 4. Discussion

It is possible to use a consumer-grade 3D printer and off-the-shelf optical and electronics components to produce a BIO that meets the International Organization for Standardization's standards for indirect ophthalmoscopes [9], as well as the American National Standards Institute's Group 1 light hazard standards for ophthalmic instruments [7]. In addition, our 3D-printed BIO is more compact and costs less than commercially available BIOs. For the cost of a single Keeler Vantage Plus indirect ophthalmoscope ($4336) [10], one could purchase a 3D printer, all the hand tools necessary to build the 3D-printed indirect, and the components necessary to build 14 3D-printed indirects.

Our 3D-printed indirect also has the advantage of being a spectacle-style design with the battery incorporated into the frame itself. Its eyepieces can accommodate different lens powers to correct for a user's refractive error. The BIO was mainly intended to be used in the clinic, but its design may offer advantages in the operating room. For example, its cordless design and ease of donning may make it useful as a BIO for scleral buckling or choroidal biopsy surgery.

The indirect can be assembled, disassembled, and repaired easily, with all its non-3D-printed parts available online. A 3D printer can be used to replace or upgrade all its 3D-printed components. These advantages make our indirect potentially useable in both resource-rich and resource-limited settings. We chose to use a $1449 3D printer capable of printing acrylonitrile styrene acrylate, a very durable material. Extremely resource-limited settings could potentially use a $349 3D printer [11] and print the indirect in polylactic acid, but the indirect would be less durable.

By making our 3D-printed parts available online, we hope to encourage ophthalmologists to print their own BIOs. Moreover, we hope that ophthalmologists will take our designs and improve on them in the future. A current limitation of our 3D-printed indirect is the length of time needed to print and assemble the components (4 h 38 min for printing and 2–4 h for assembly). Design improvements and new methods of 3D printing may shorten these times. An incorporated blue light filter and wireless video recording capability could be features added to the indirect in the future.

## 5. Conclusions

3D printers and CAD programs allow for rapid prototyping through an iterative process, and they are becoming more affordable and easier to use. These advantages helped us create a low-cost, lightweight BIO with the battery incorporated into the frame. We expect that 3D printing will lead to additional improvements in existing devices in ophthalmology (such as the BIO), as well as the production of novel devices in our field.

## Data Availability

The data that support the findings of this study are available in the Supporting Information of this article.

## Nomenclature

BIO: Binocular indirect ophthalmoscope
CAD: Computer-aided design
LED: Light-emitting diode

## Supporting Information

Table: Components and toolslist.

Step-by-step build guide.

Video: Printing of 3D components and features of the 3D-printed BIO.
