# Supplementary material for: Low-Cost 3D-Printed Binocular Indirect Ophthalmoscope
Source: J Ophthalmol. 2025 Apr 17;2025:5638606. doi: 10.1155/joph/5638606 (PMC12021479; doi:10.1155/joph/5638606)

V4.9 Complete Build Guide

# Material List

## 3D Printer and filament

We recommend using ABS or ASA for printing rigid components and TPU for the flexible components. ABS and ASA require an enclosed 3D printer to achieve proper print temperatures. We used the [Bambu Lab X1C](https://us.store.bambulab.com/collections/3d-printer/products/bambu-lab-x1c-3d-printer). A cheaper alternative that still retains an enclosure is the [Bambu Lab P1S](https://us.store.bambulab.com/products/p1s?variant=42153262743688).

You will need at least one roll of rigid filament (we recommend black if using only one) and one roll of flexible filament. Here are some filaments we have tested and recommend.

Rigid:

[Bambu Lab ABS](https://us.store.bambulab.com/products/abs-filament)

[Hatchbox ABS](https://www.amazon.com/dp/B00J0H8EWA?ref=ppx_yo2ov_dt_b_product_details&th=1)

[Polymaker ASA](https://www.amazon.com/dp/B09DKPYYBP?psc=1&ref=ppx_yo2ov_dt_b_product_details)

[Polymaker ABS](https://www.amazon.com/dp/B0B2P3D3YB?ref=ppx_yo2ov_dt_b_product_details&th=1)

Flexible:

[Overture TPU](https://www.amazon.com/dp/B07VDP2S3P?psc=1&ref=ppx_yo2ov_dt_b_product_details)

[Bambu Lab TPU](https://us.store.bambulab.com/collections/pc-tpu/products/tpu-95a-hf)

Budget option: If you have a printer that cannot print ABS or ASA, PLA can be used as an alternative though it will not have the same heat resistance. Reduced heat resistance may result in warping and consequently, diplopia of the optical arrangement if left in a hot car for example.

##

## Tools
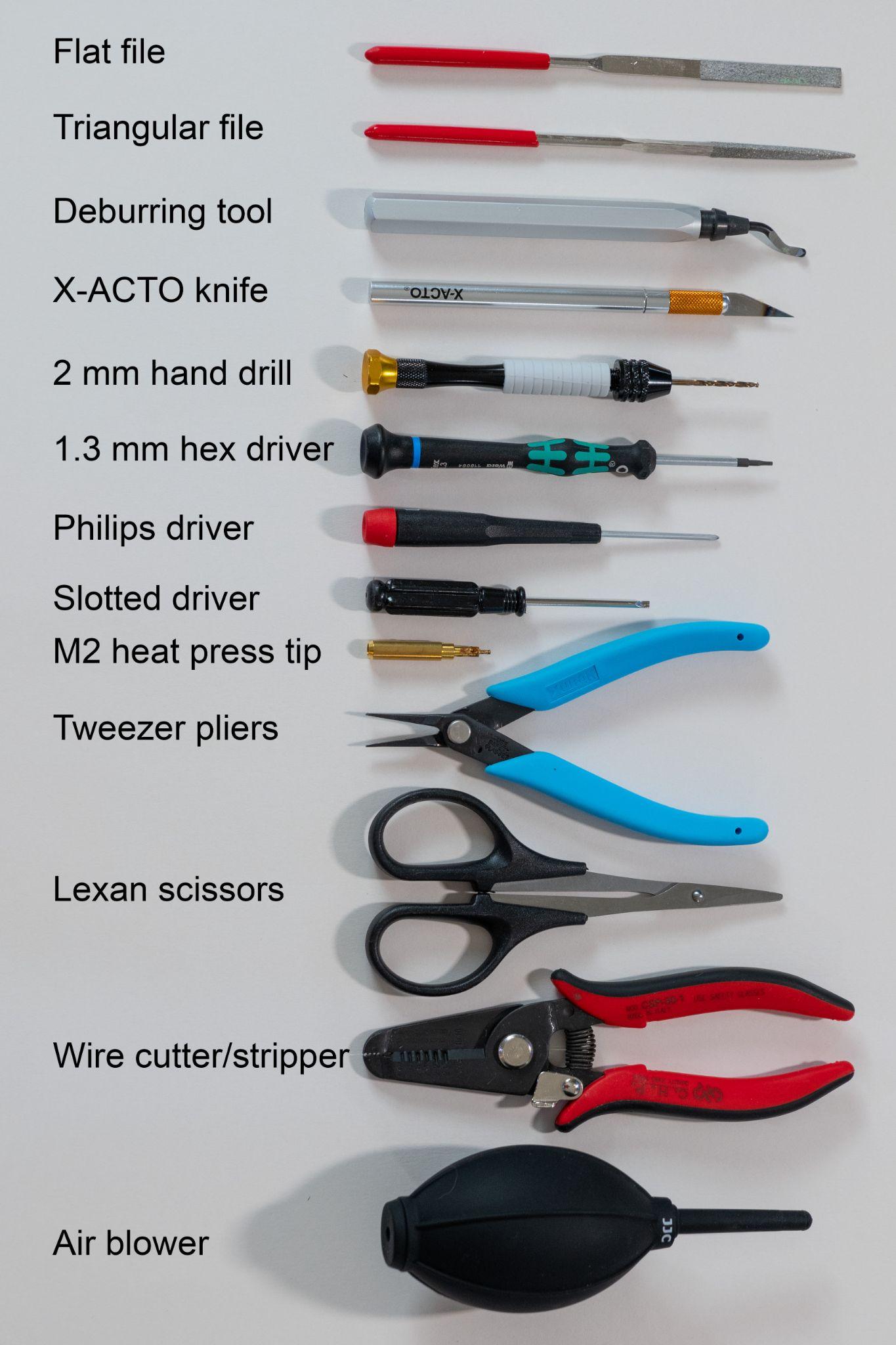


List:

[File set](https://a.co/d/0cJyeSkE)

[Deburring tool](https://a.co/d/0grnPk81)

[X-ACTO knife #1](https://a.co/d/01tJDt8U)

[Micro hand drill](https://a.co/d/0g10ezSh)

[Hexagon screwdriver 1.3 mm](https://www.amazon.com/dp/B003ES5MDU?psc=1&ref=ppx_yo2ov_dt_b_product_details)

[Slotted and Philips screwdriver set](https://www.amazon.com/Wiha-26197-Precision-Phillips-Screwdriver/dp/B01L46TEN2/ref=sr_1_2?dib=eyJ2IjoiMSJ9.89mbbfE1EXCp0e3Bla8zbk5CYaxR3gBuvICN6X3t6J_AbUGcNRcpny7ATpBMdlHDEusZC91VAu4W_fztnw5S8BDBWM2nuJBrrGhld3mzWBAdwzCCxBcIOjkW_Rd7dabXmANTDnDX36iSV6_RriS2i4TcBPcdClDWr1R0r-IRa1jyj0b-CyGcz0X8-lWmZNAJgvyAu7Qo6Yzh1v560ul_W4N0nlGTFgaIkA95b0CtUaAb5cFQryPTHy0OJ1pFS_E1siObdZkQMFdK5zS6JLqLgGogDDa86VkBFAY5zPfF3ao.v6hCDNkVFzHgxjNf6OIp3jvkF5ul2myj861hm0utTiE&dib_tag=se&keywords=slotted%2Band%2Bphilips%2Bscrewdriver%2Bset&qid=1722209412&sr=8-2&th=1)

[Heat press insert tip M2](https://www.amazon.com/Virtjoule-Heat-Insert-Tips-Sizes/dp/B08B17VQLD/ref=sr_1_8?crid=2KN7N1BAOKU3O&dib=eyJ2IjoiMSJ9.QRxwgsm2oiTZifw97eoLrX9JX6zvqyoKBb4Nv8nAAJser_EG4CBpZIcPEpb6rI3Mz5nOK3Kjchy9M_QuItIaRz85hfHA3STrbiEg33iyQrbmB4eHlNrSOM5Y7DNJYYSXHAeF1BECEDEaPQXuAXgyepLhhx4B73-abdiVhuQVXqP4nNH9MR2gGdIAXwKw3sSM-L_RAaBHq_Mnja7bY7MTGLcOwVdU7M8u72_I6VuayHi0Kq21c_ZEQze_Ii_EQob3G0JvlCPtC7Iq0mBKdpCZRWVG2IowYGpNU4WCT1srDAY.5oQXVxC8IomiOd9b_D4uBKvjbDu7uPXIa_E1xI36hW0&dib_tag=se&keywords=heat+press+insert+tip+M2&qid=1722209481&s=hi&sprefix=heat+press+insert+tip+m%2Ctools%2C132&sr=1-8)

[Tweezer nose pliers](https://a.co/d/0ipWidOs)

[Lexan scissors](https://a.co/d/0ea84PAp)

[Wire cutter and stripper](https://a.co/d/03I5aMOY)

[Air blower](https://a.co/d/0apM7urn)

[Solder station](https://a.co/d/004Xbgtf)

[Lighter](https://www.amazon.com/BIC-Multi-purpose-Lighters-Fireplaces-Assorted/dp/B00GUQWAS8/ref=sr_1_5?dib=eyJ2IjoiMSJ9._tBizJFR0JTV8DfXchiUuudoD3DAmdIMnk5rXjYjxYW-TBXbYPa_tICVgZHaKNoGtRFdskZsYVwoeRndQwIr0Dd5Ew4vhO8YP1tDTUPxzhGMIeUSjE_qaRhiI3o_vWXQ4BRSm-ZozIjRaM5EJmYHc0EsVKtZy6JCv4Fsm02LrA-5KjzgHk3dcVrMCy8aea7a6m39CQEED82A9dGN-Mx1bRS2h6CrgUYCsuARglyTIJngEYpzbHbijoDL-7bL4ynopQIVk7sN6NZVo13cJRzlaGl9x-UePehEyZw3bP2GEL0.xT0ywh7sW_bhGgliTuK5_C49ayKDXlNhO2WpgYgmPc0&dib_tag=se&keywords=lighter&qid=1722140315&sr=8-5&th=1)

Optional:

[Electronic grade silicone adhesive sealent](https://a.co/d/1trs8Jc)

[Liquid flux](https://a.co/d/6lquObM)

[Silicone grease](https://a.co/d/elEeXiI)

[Wet sandpaper 400 grit](https://a.co/d/aPvy9J9)

**Figure 1: Tools**

##

## Components
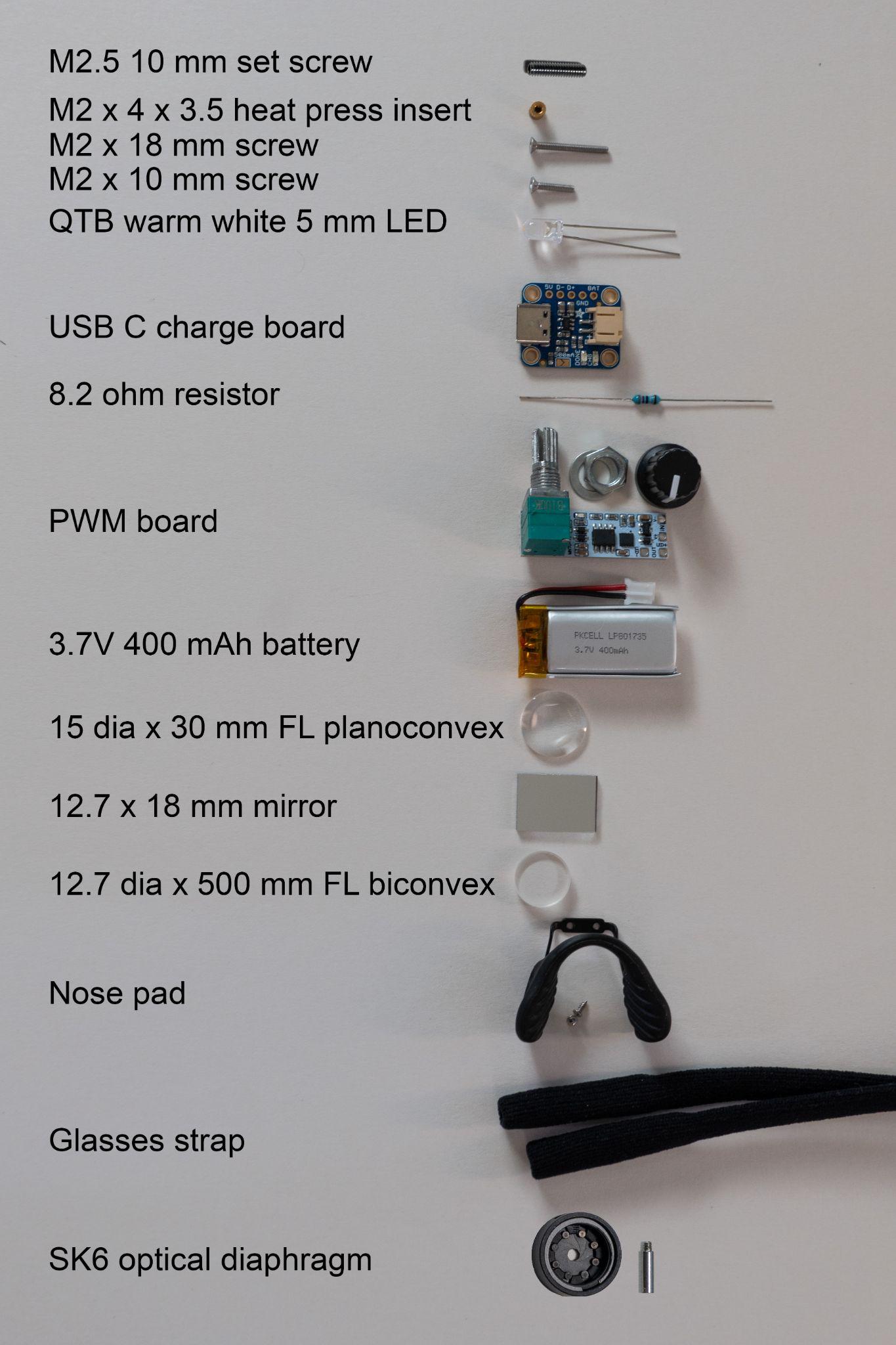


List:

[M2.5 10 mm set screw](https://www.mcmaster.com/92605A073/)

5x [M2 x 4 x 3.5 insert](https://a.co/d/0bkjrB9r)

3x [M2 x 18 mm screws](https://www.mcmaster.com/92125A101/)

6x [M2 x 10 mm screws](https://www.mcmaster.com/92125A056/)

[QTB warm white 5 mm LED](https://www.digikey.be/en/products/detail/qt-brightek-qtb/QBL8IW15C-WW/10441157)

[USB-C charge board](https://www.adafruit.com/product/4410)

[8.2 ohm resistor](https://a.co/d/049ot1eG)

[PWM board](https://www.icstation.com/driver-dimmer-module-stepless-potentiometer-circuit-control-board-p-16056.html)

[3.7V 400 mAh battery](https://www.adafruit.com/product/3898)

[15 dia x 30 mm FL planoconvex](https://www.edmundoptics.com/p/150mm-dia-x-300mm-fl-uncoated-plano-convex-lens/2079/)

4x [12.7 x 18 mm mirror](https://www.edmundoptics.com/p/127-x-18mm-4-6lambda-mirror/26616/)

2x [12.7 dia x 500 mm FL biconvex](https://eksmaoptics.com/optical-components/lenses/bk7-biconvex-lenses/)

Surgitel Ergo Fit Nose Pad

[Glasses strap](https://a.co/d/06WBZk8T)

[SK6 optical diaphragm](https://a.co/d/0gUV6ELP)

[Polycarbonate sheet](https://a.co/d/iRZZjpw)

[Cyanoacrylate glue](https://a.co/d/1Trtq8A)

[26 AWG wire](https://a.co/d/048E1Ht7)

[63/37 solder](https://a.co/d/0dsUC8aa)

[Heat shrink](https://a.co/d/0bjx68EG)

For optional blue filter accessory:

[Blue gel filter](https://a.co/d/aAxwNum)

Note: Optical and electrical components necessary to meet ANSI light safety standards according to our testing are highlighted.

**Figure 2: Components**

# 3D Printing instructions

## 3D printed components
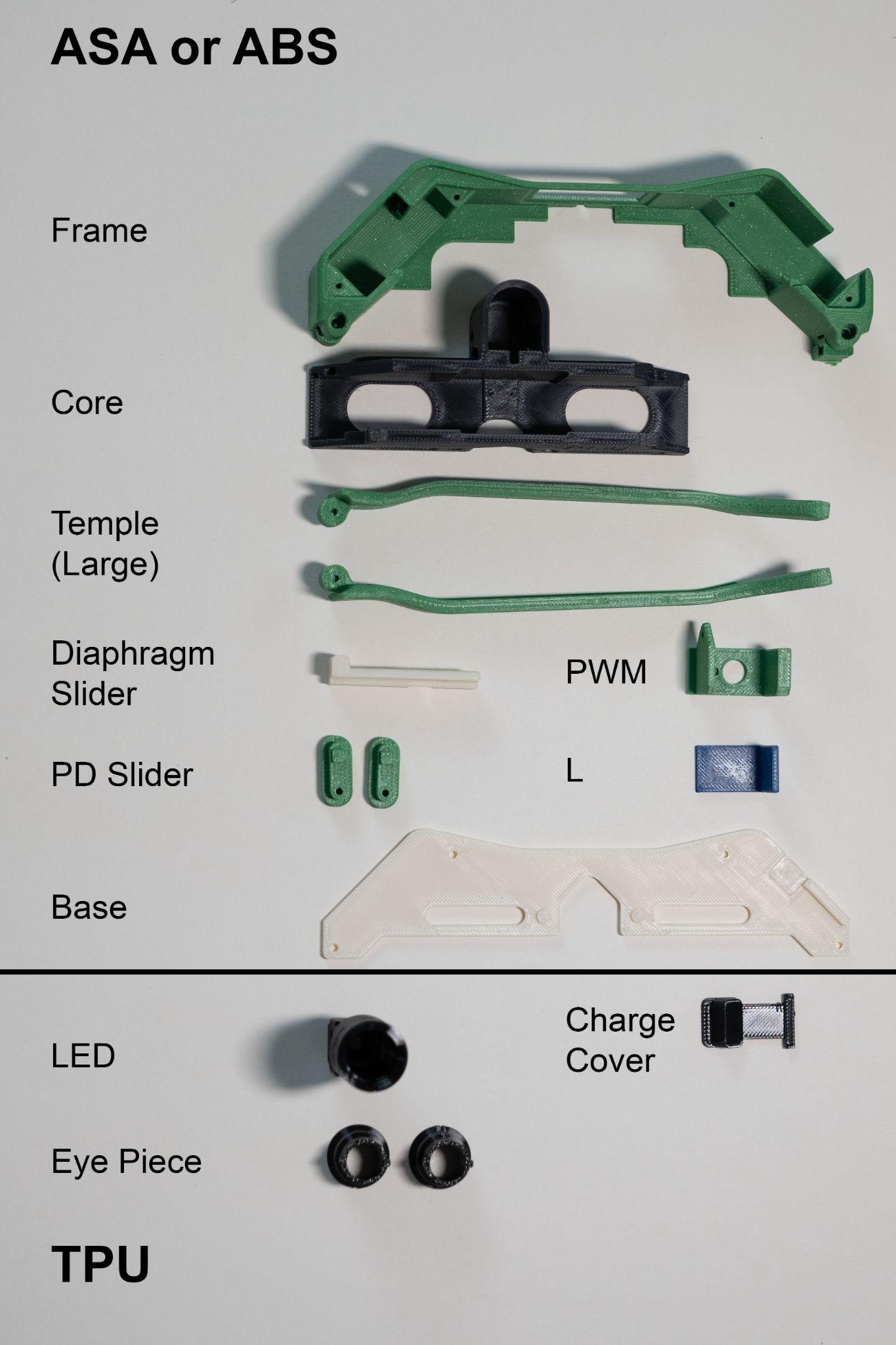


Print these with ASA or ABS:

- Base
- Core
- Diaphragm Slider
- Frame
- L bracket
- Mirror Center (not pictured)
- Mirror Left (not pictured)
- Mirror Right (not pictured)
- PD Slider (print 2x)
- PWM
- Temple Left (chose size)
- Temple Right (chose size)
- Template Polycarbonate Window (not pictured)

Print these with TPU:

- Charge Cover
- Eyepiece (print 2x)
- LED holder
- Washer for nosepiece

Print these with transparent PETG:

- Charge Indicator

Optional for blue light filter accessory:

- Template Blue Light Filter

**Figure 3: 3D Printed Components**

##

## Slicing and Printing

Open STL files in your preferred slicing software (we used Bambu Studio or OrcaSlicer). Some settings may vary depending on the filament or printer. Here are a few of our recommendations:

- Nozzle size 0.4 mm
- Layer height: 0.20 mm for all parts
- Wall printing order: Outer/Inner (for dimensional accuracy)
- Wall loops: 3
- Top shell layers: 5
- Bottom shell layers 3
- No support or brim unless specified below
- Print all optical components in black (Core, Mirror Center, Mirror Left, Mirror Right, TPU Eye Piece, TPU LED)

Part specific recommendations

- Core: enable support but use support blocker as shown in figure 4
- Diaphragm Slider: enable support and use brim
- Frame: enable support but use support blocker as shown in figure 5
- Temple: enable support and use brim
- TPU Charge Cover: enable support


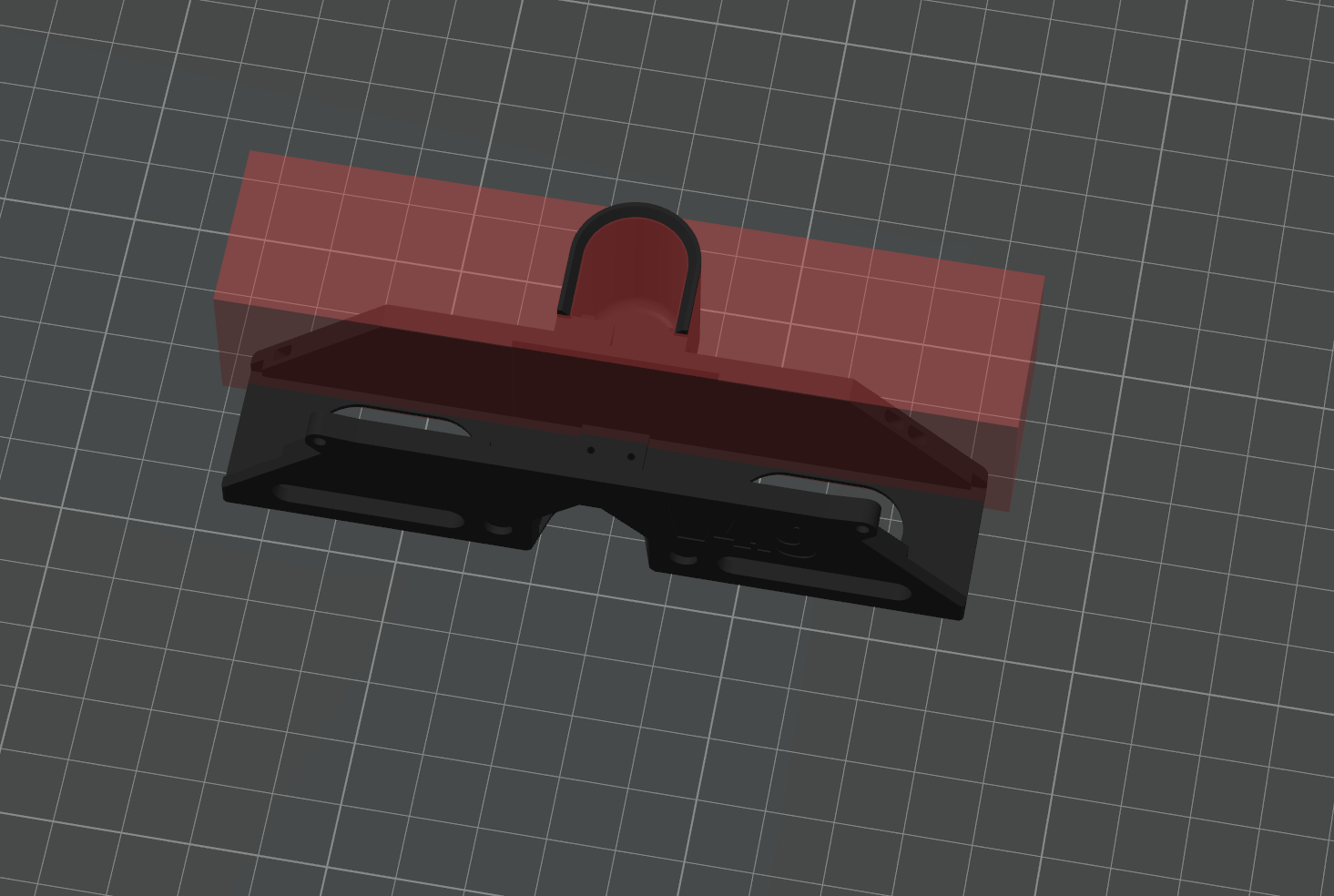

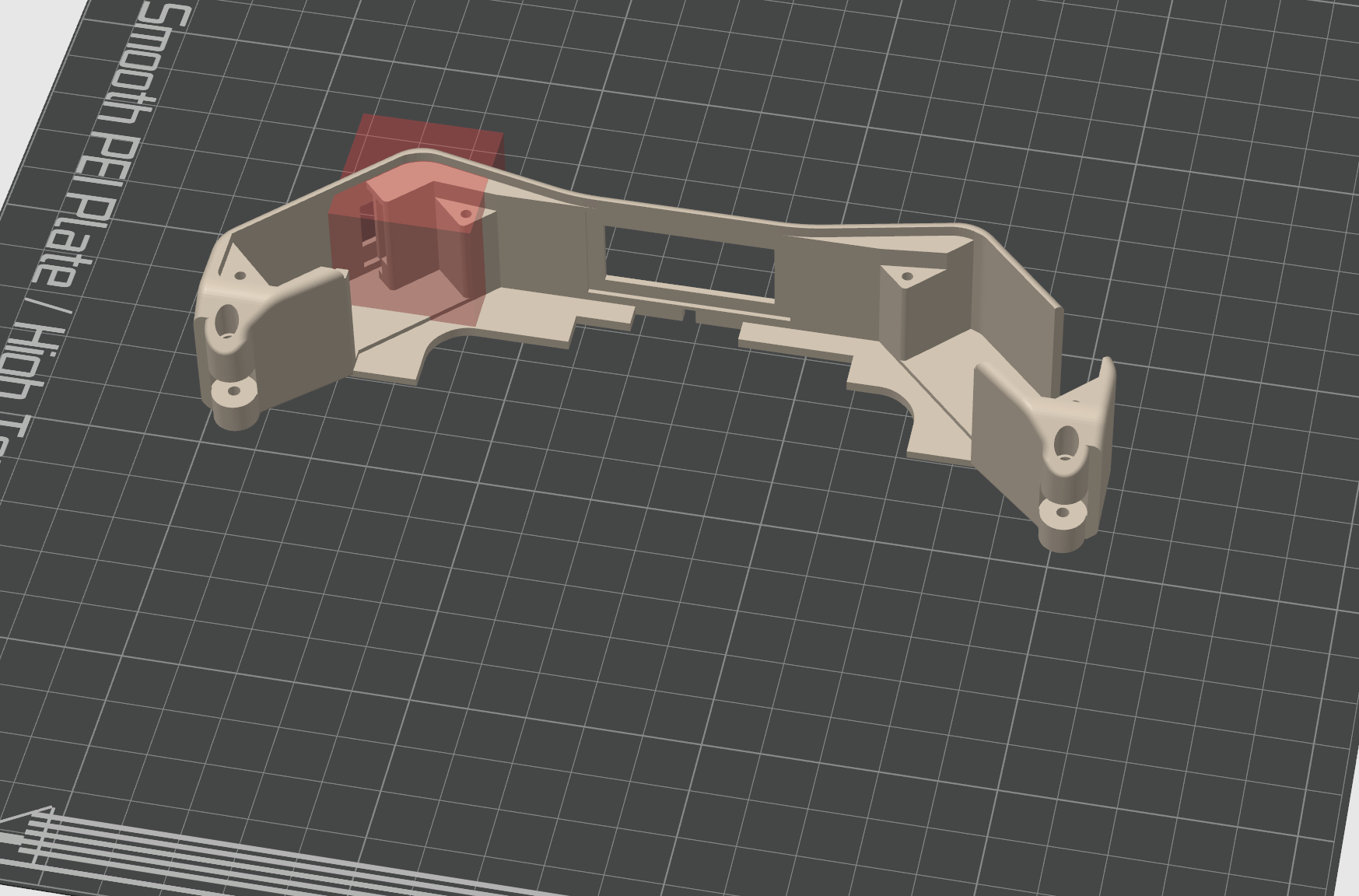


#

#

**Figure 4: Core support blocker settings Figure 5: Frame support blocker settings**

# Post-processing printed parts

Tools:

- Flat file
- Triangle file
- Fine pliers
- Hand drill with 2 mm bit
- 400 grit sandpaper

## Core

- Remove supports.
- File rough edges. Use flat file to file roof (Figure 6) and floor (Figure 7) where the central mirror slides. Use triangle file to file vertical edge where diaphragm slides (Figure 7).


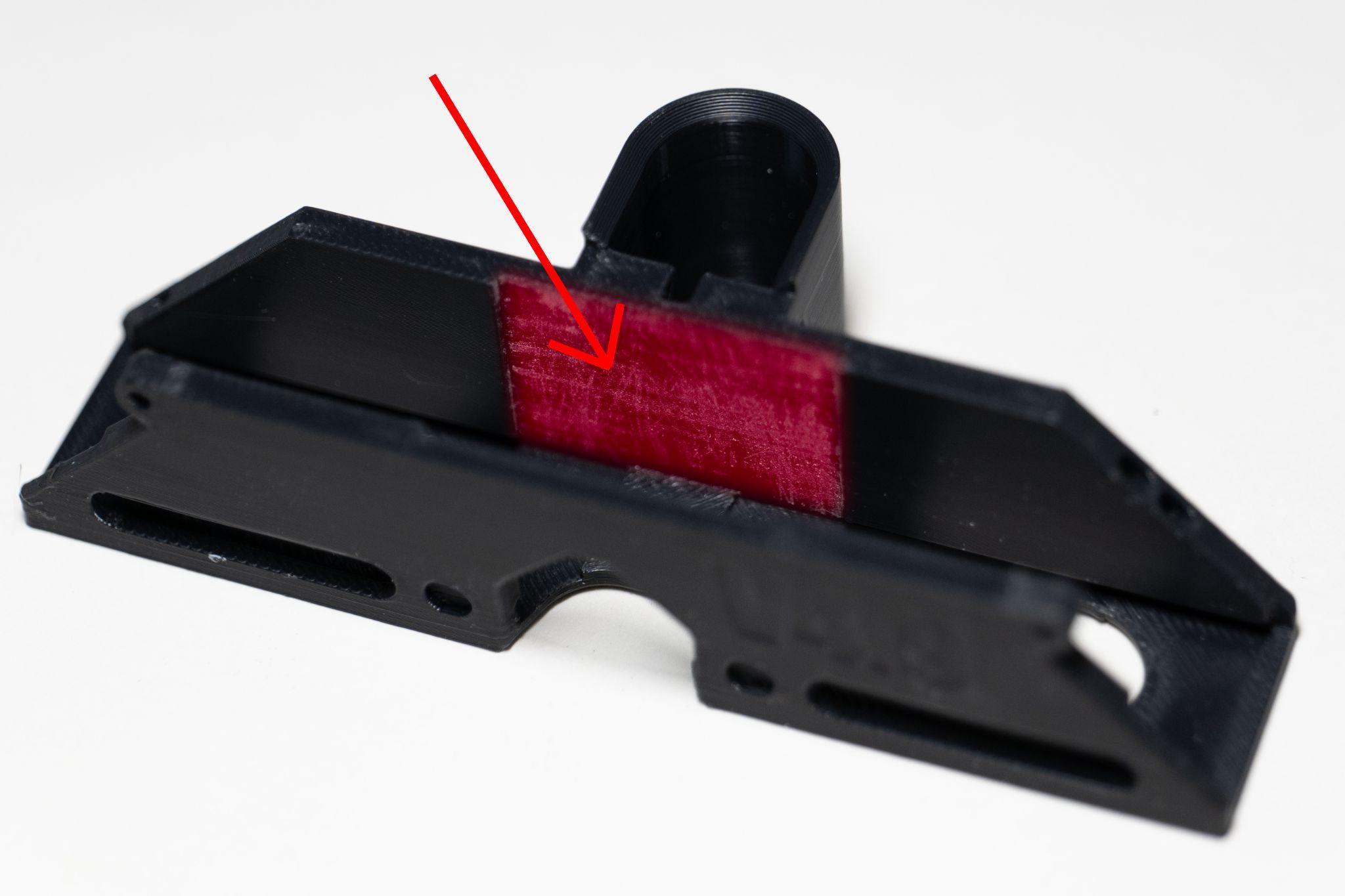

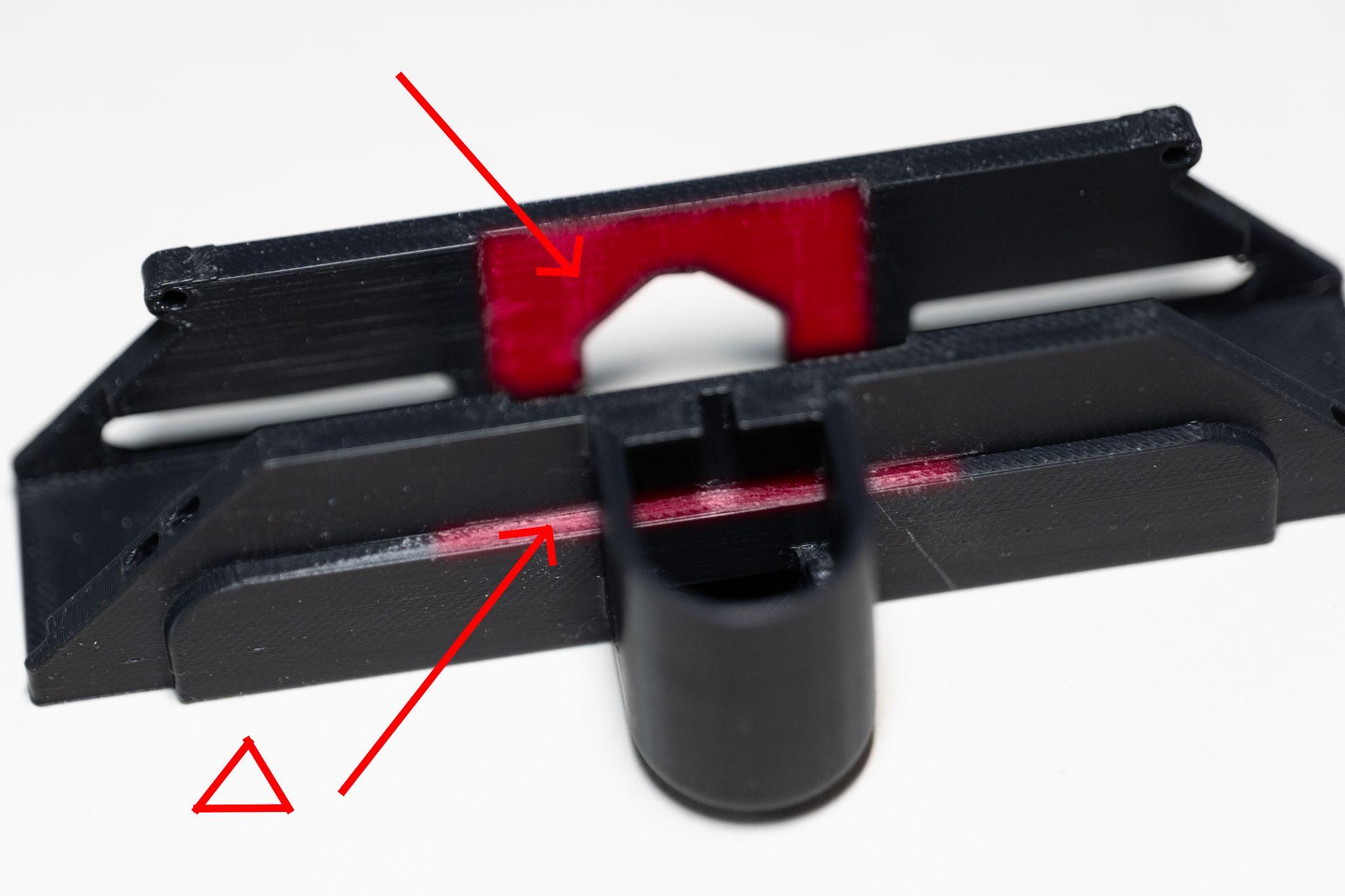


**Figure 6: File shaded area Figure 7: File shaded area**

## Diaphragm slider

- File diaphragm slider on surfaces that touch core (Figure 8 and 9)
- Test fit slider in core. There should be some resistance.


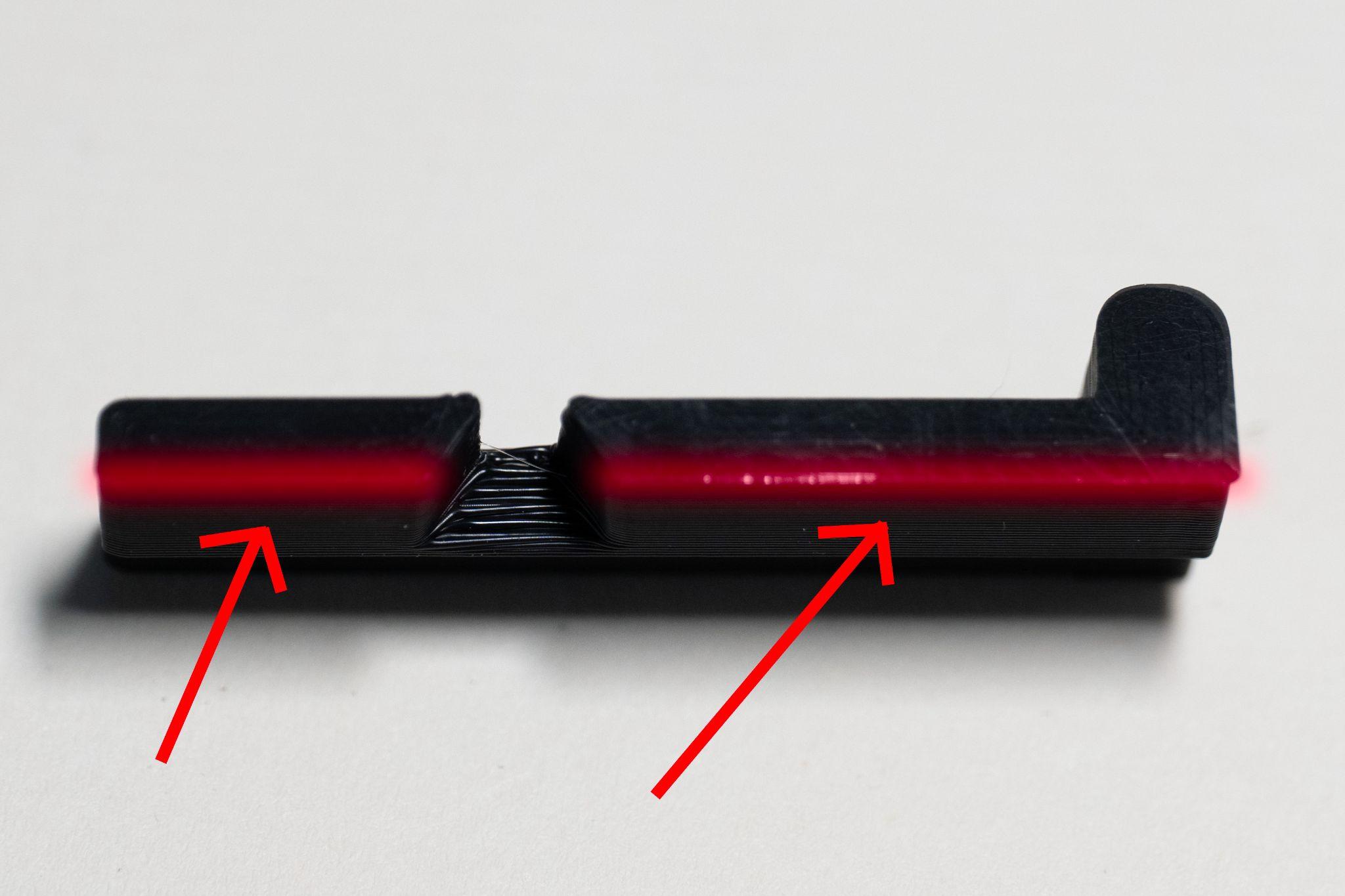

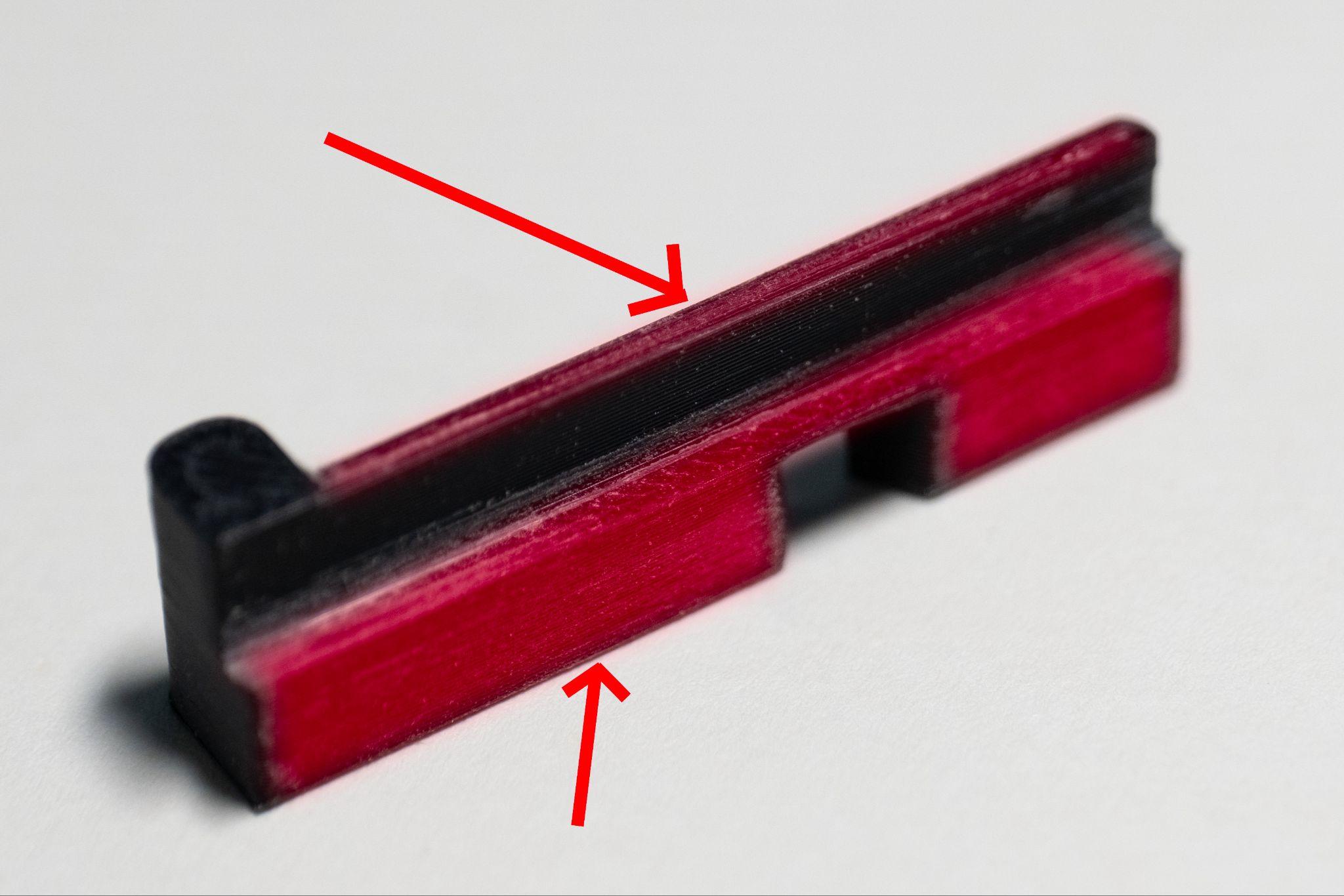


**Figure 8: File shaded area Figure 9: File shaded area**

## Frame

- Use tweezer pliers to remove supports.
- File inside window frame with tip of flat file (Figure 10) to help with polycarbonate window adhesion.
- Drill through holes with 2 mm bit (Figure 11).


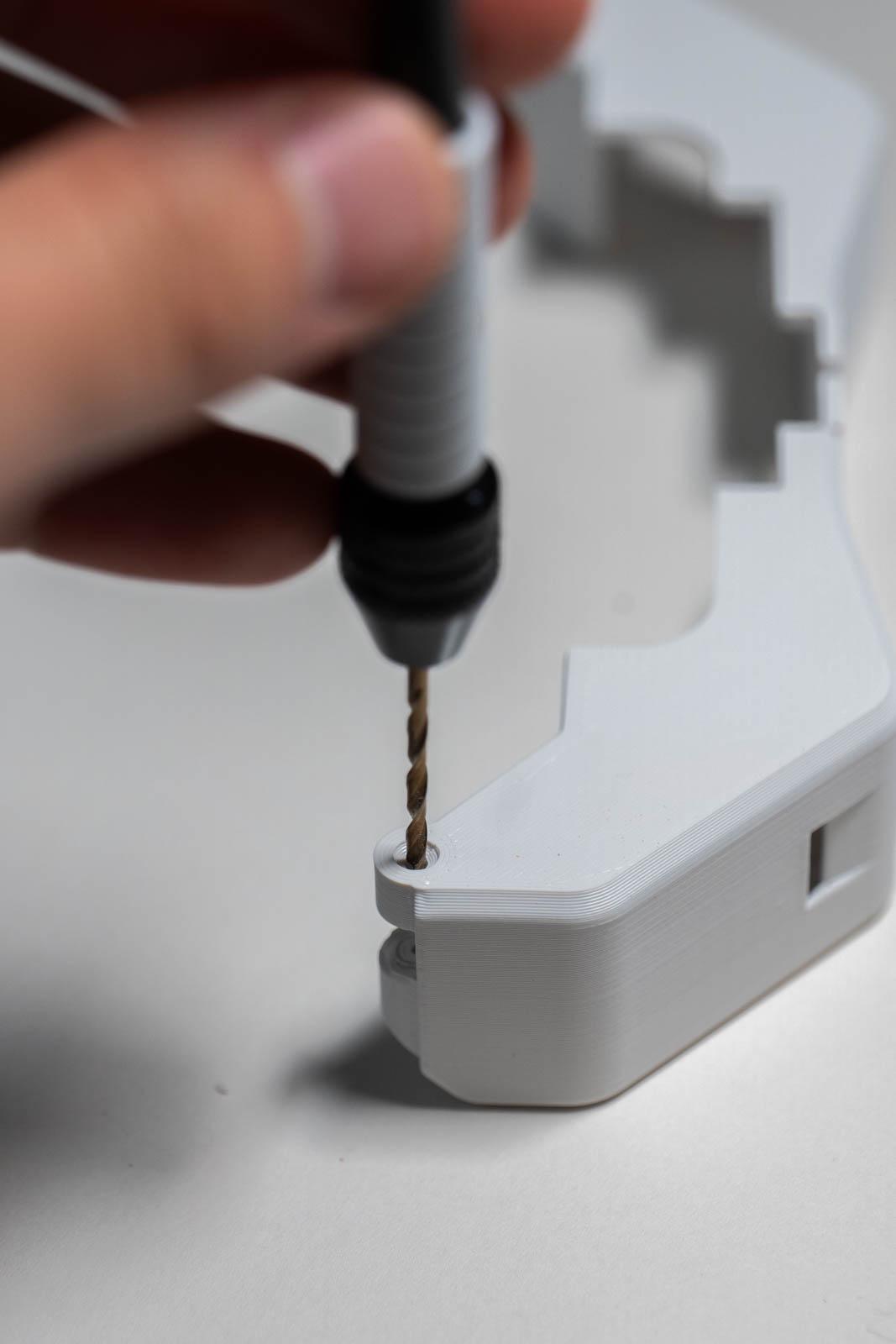

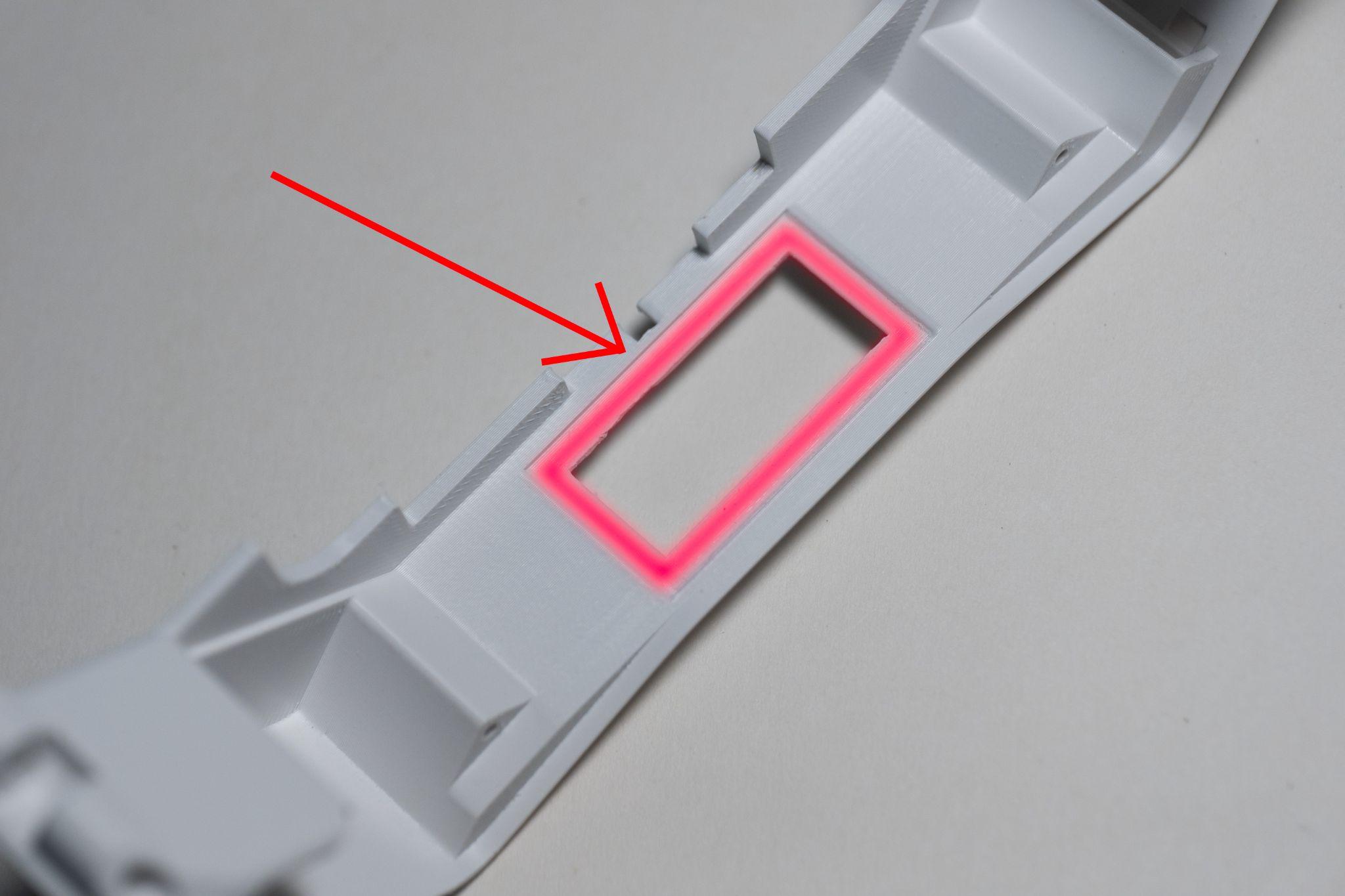
**Figure 10: File shaded area Figure 11: Drill frame**

## Temples

- Remove supports and brim.
- Lightly deburr top edge.
- File inferior edge and any other sharp edges. File hinge if necessary to fit frame.
- Drill through hinge holes with 2 mm bit.
- Optional: wet sand inferior edge.

## Base

Lightly file top of print surface to make sure it is smooth where it contacts the frame.

## Pupillary sliders

Drill through holes with 2 mm bit.

##

## Central mirrors

- File any flanged edges and surfaces that slide in core (Figures 12 and 13)
- Insert grub screw until screw is flush with top surface (Figures 12 and 13)
- Insert mirrors. Tip: using gloves can reduce smudges when installing mirrors or lenses.


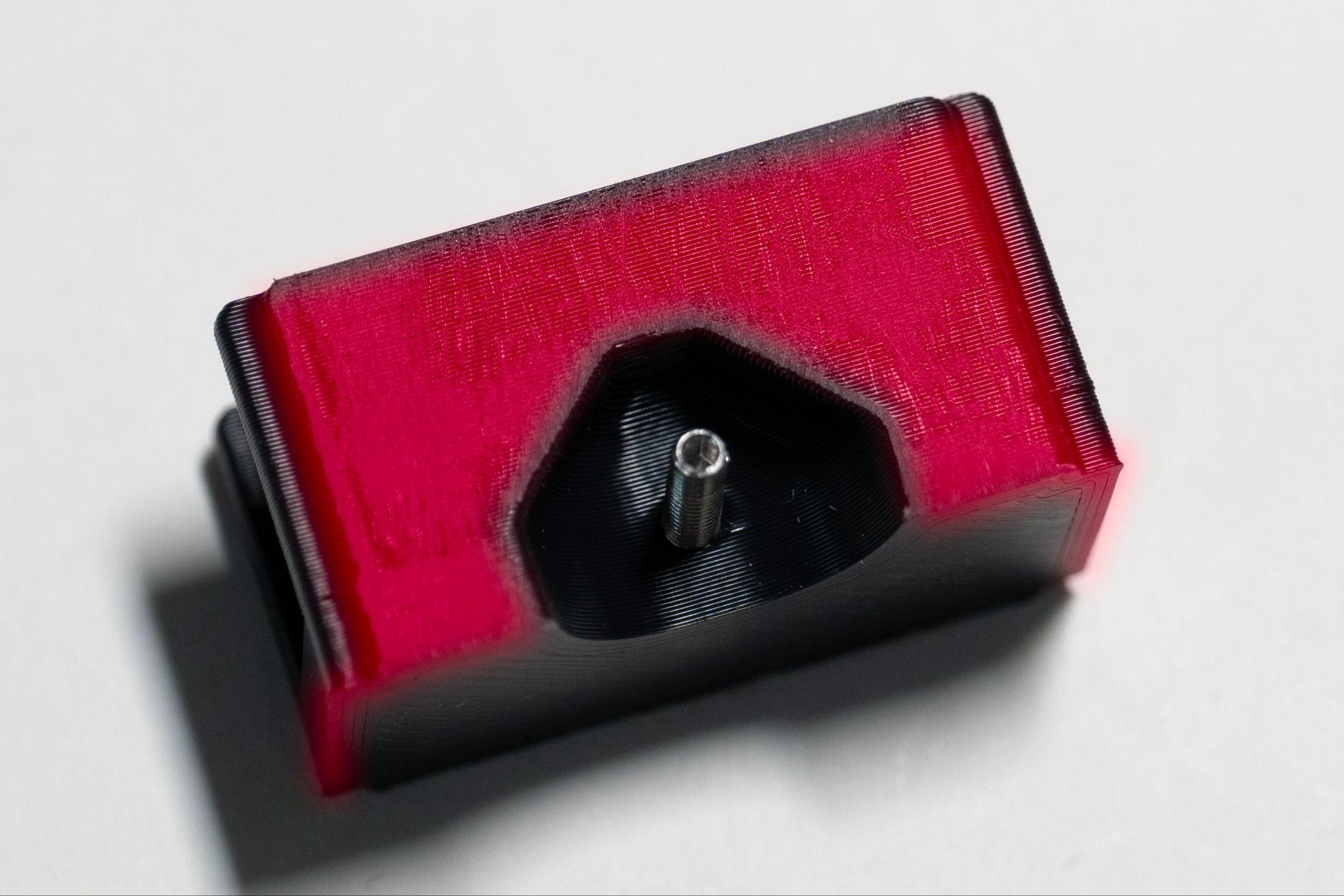

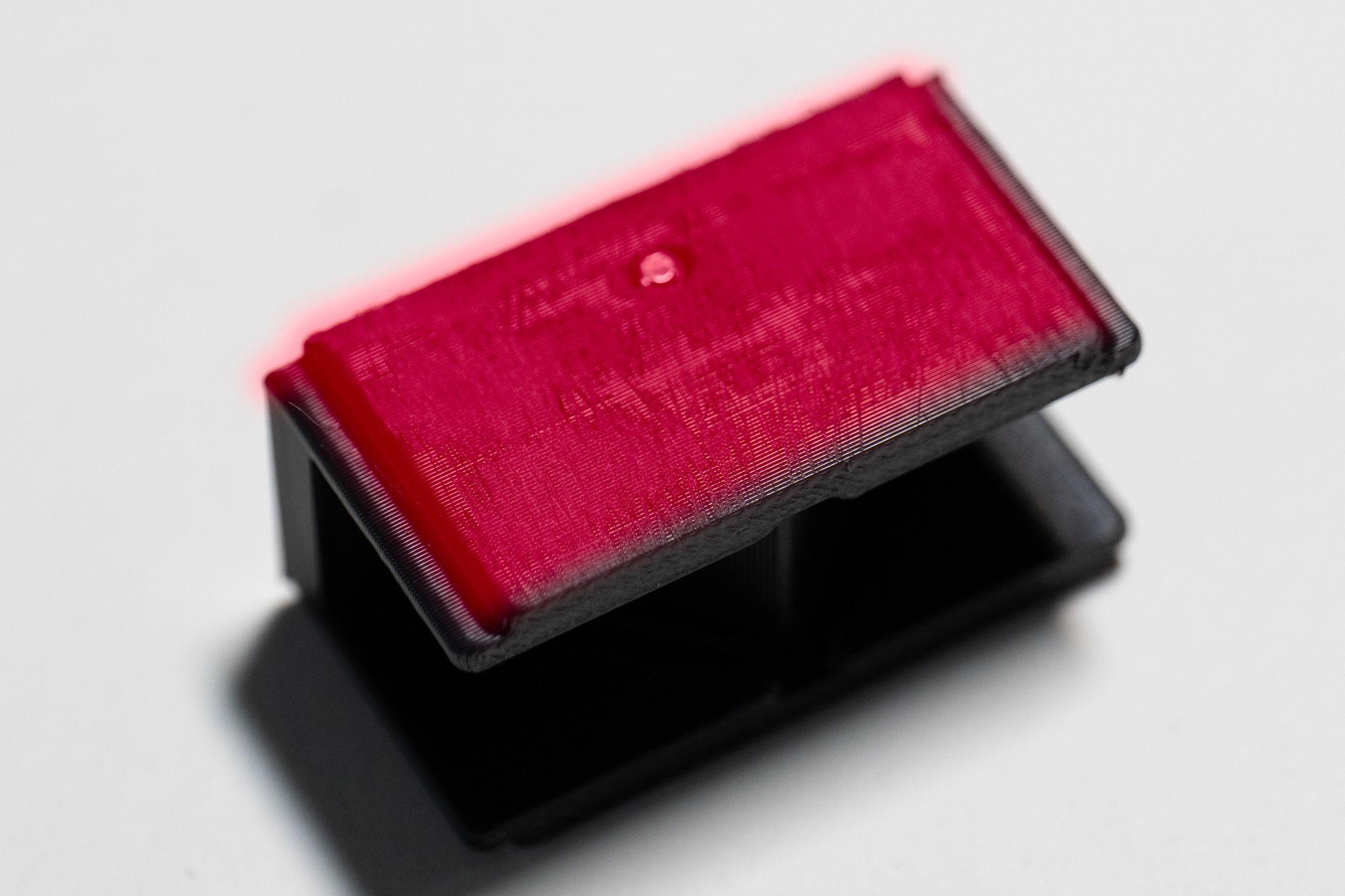


#

#

**Figure 12: File shaded area Figure 13: File shaded area**

## Side mirrors (2x)

- Use flat file to file inside edges for mirror fitment (Figure 14).
- Carefully insert mirror (Figure 15).
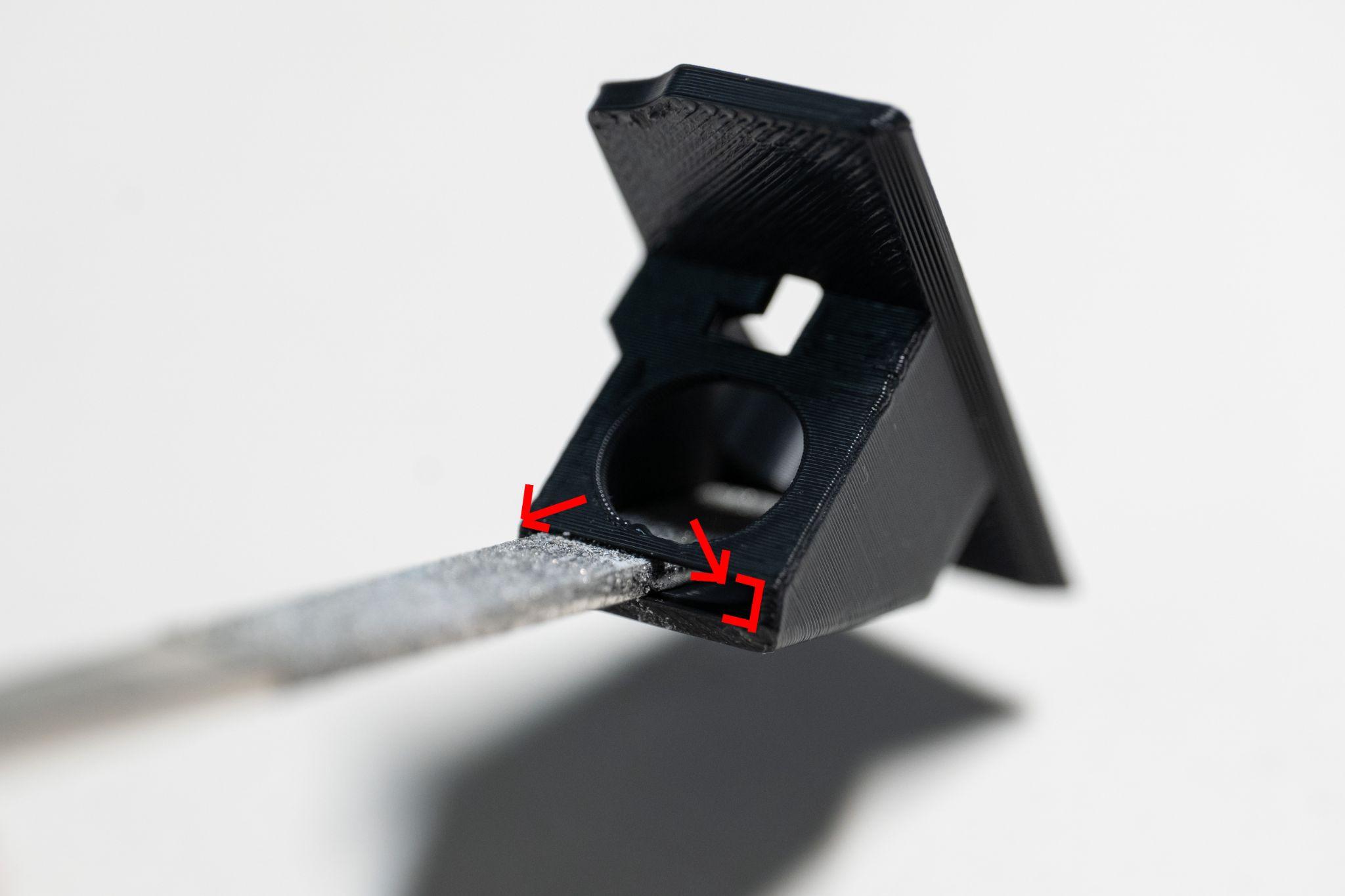

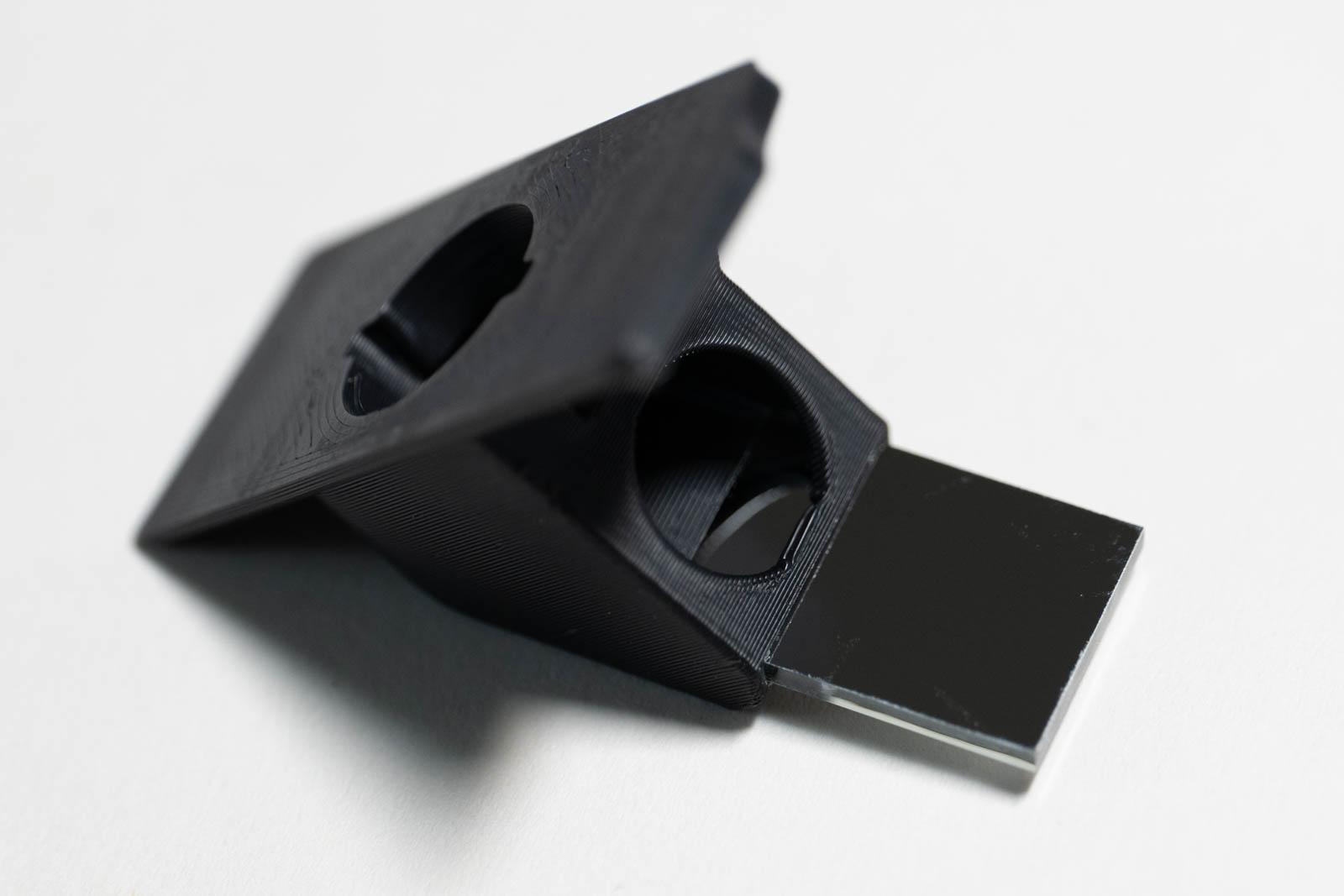


#

#

**Figure 14: File corners of recess Figure 15: Carefully insert mirror**

#

# Heat press insert installation

## Core

1x M2x4x3.5 insert on user’s left side of light assembly housing.

- Insert with M2 insert tip at 265 C if using ASA or ABS. CAUTION when insert is almost flush, it has a tendency to push through too far. Make sure heat set insert is completely flush to user’s left side (Figure 16).
- Cut excess plastic inside core.
- Thread M2 bolt from outside to inside of insert to clear any residual plastic.
- Trim or file any remaining plastic inside core around insert.


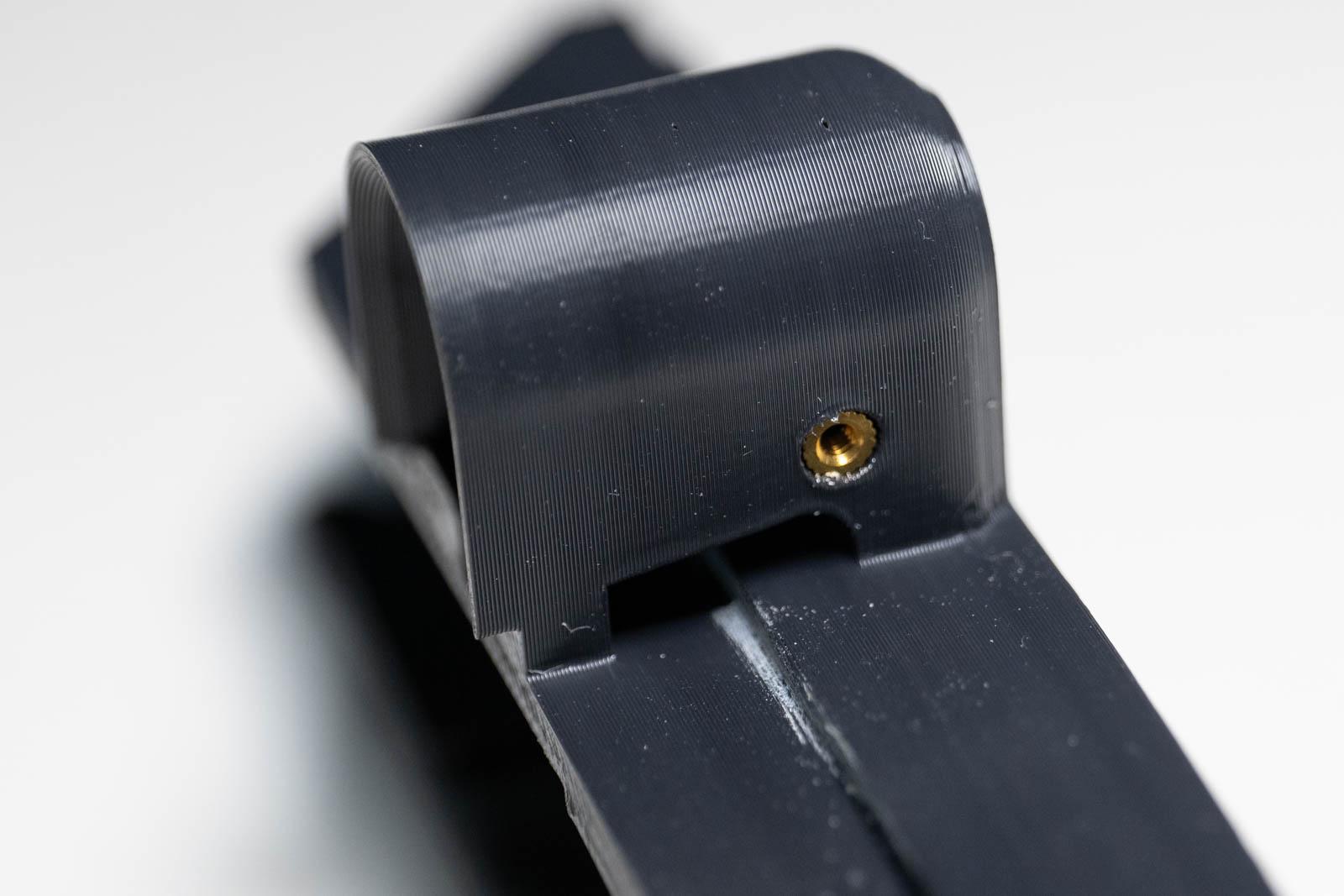


**Figure 16: Core heat pressed insert**

## Frame

2x M2x4x3.5 inserts, one for each temple hinge.

- Press until flange of solder iron almost contacts the frame as shown (Figure 17).
- Thread M2 through insert to clear any residual plastic.


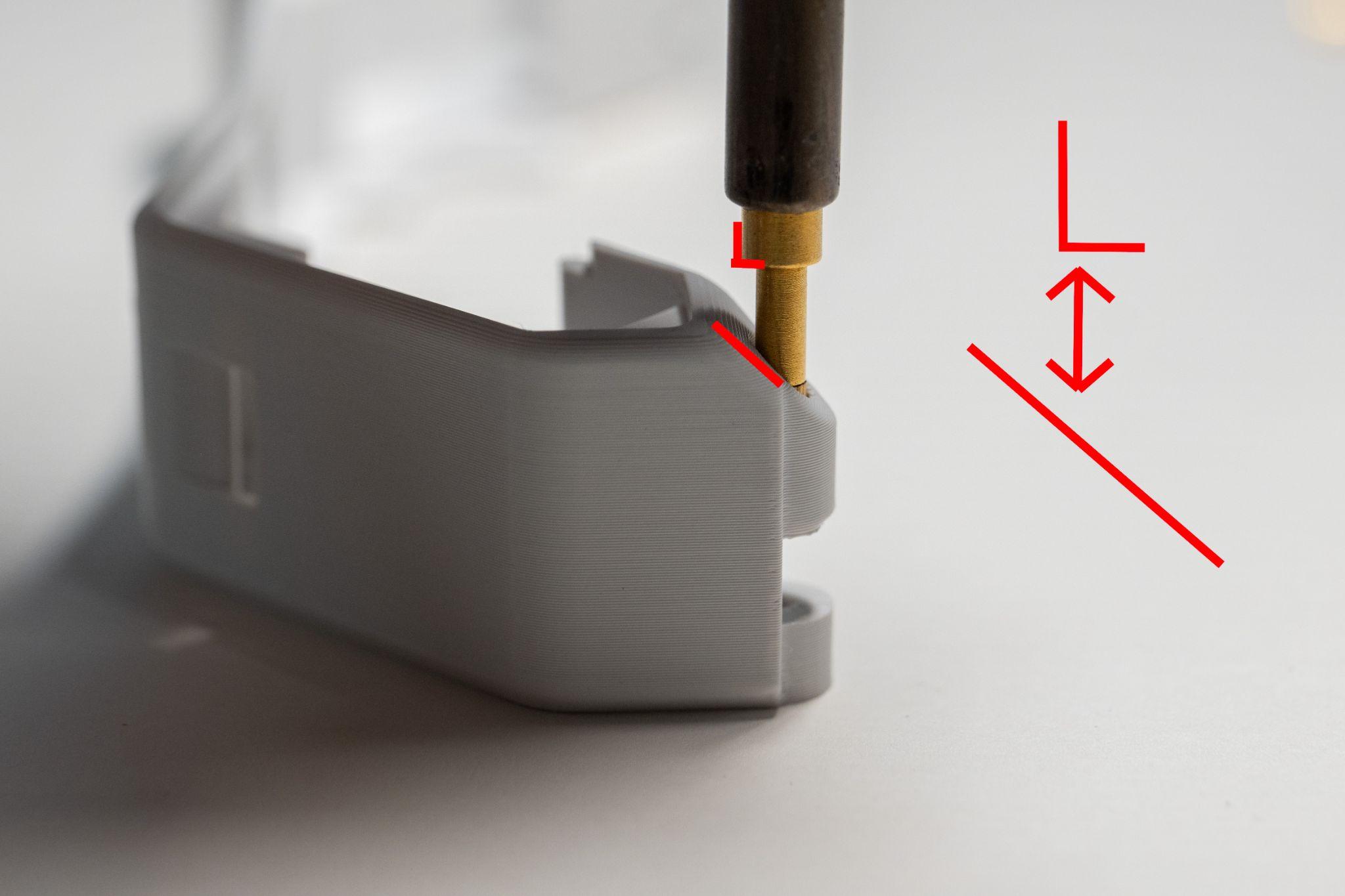


**Figure 17: Frame insert depth alignment**

## Side mirrors

2x M2x4x3.5 inserts, one for each component.

- Insert into existing hole and press until flush with surface.

# Core electronics

## Install wires in core

Thread wires through channels from right to left as seen in Figure 18 with attention to wire color order. Leave excess wire length for installing electronics.


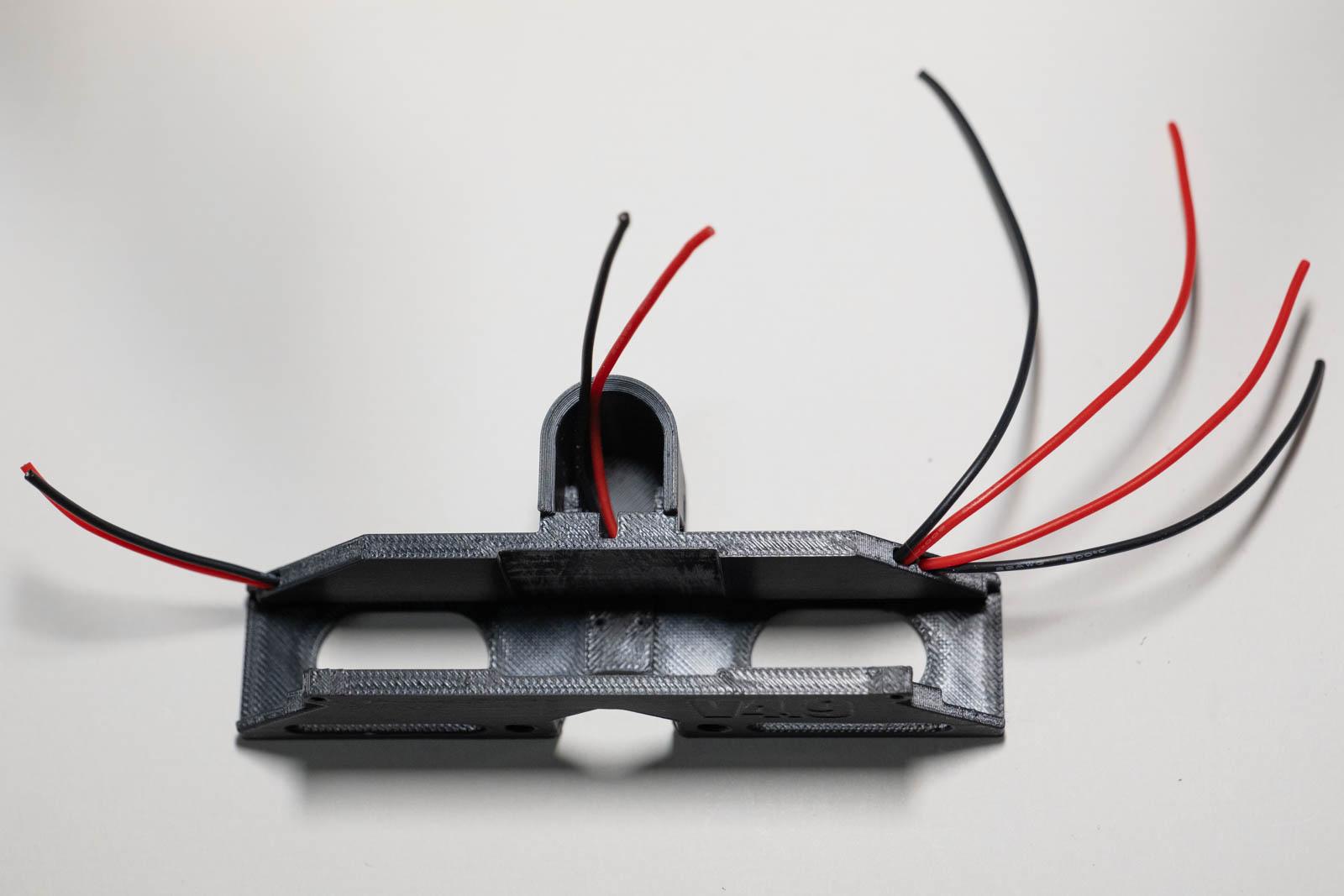


**Figure 18: Core wire installation**

## Solder 5 mm LED

- Strip wire ends in the LED housing 3 mm (Figure 19).
- Tin wire ends. Optional: applying flux can help solder adhere better.
- Cut 4 mm (outer diameter) heat shrink to 8 mm length and slide over wires in LED housing.
- Cut LED leads to 6 mm and 7 mm to indicate polarity with the longer wire positive. You can use the flat region of the LED wire as a landmark and cut 1 mm and 2 mm distal.
- Solder LED to wires at 370° C leaving at least 2 mm between the wires and base of LED. QuadHands may be helpful! Make sure there is good overlap between the wire and LED lead as this is a moving part due to the LED hinge.
- Slide heat shrink over solder joint and shrink with lighter being careful not to damage LED (Figure 20).

##
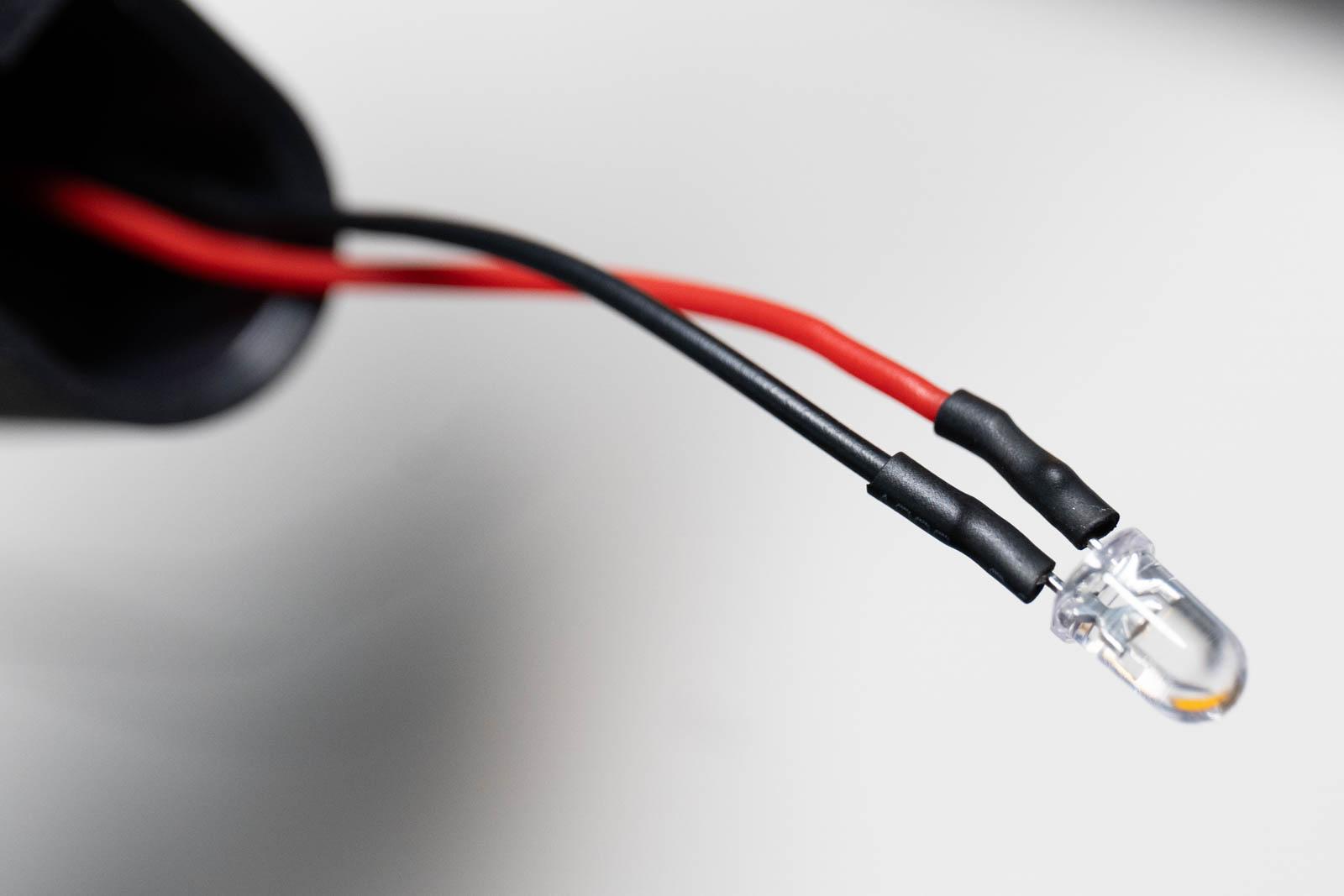

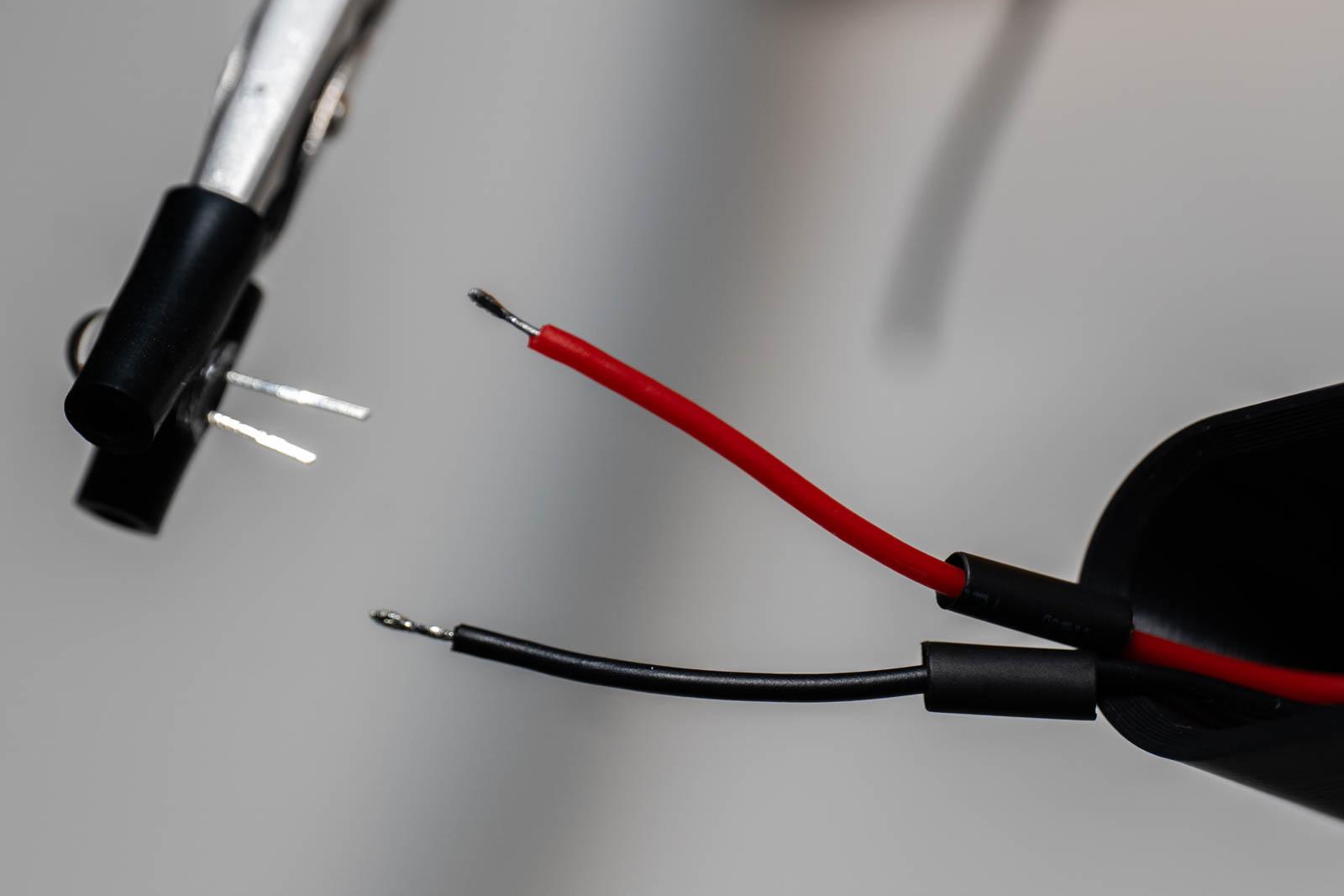


##

##

##

**Figure 19: LED solder preparation Figure 20: LED soldering complete**

## Solder charge board

- Identify the two wires exiting on the left when core is facing you.
- Strip wire ends 5-8 mm
- Optional: apply flux to stripped wire ends and charge board “GND” and “BAT” terminals.
- Hold charge board with QuadHands and insert black wire partially into “GND,” apply solder, and press the rest of the way through (Figure 21).
- Repeat with red wire going into “BAT.”
- Apply solder to opposing side of board (Figure 22) then trim wire ends flush with board.


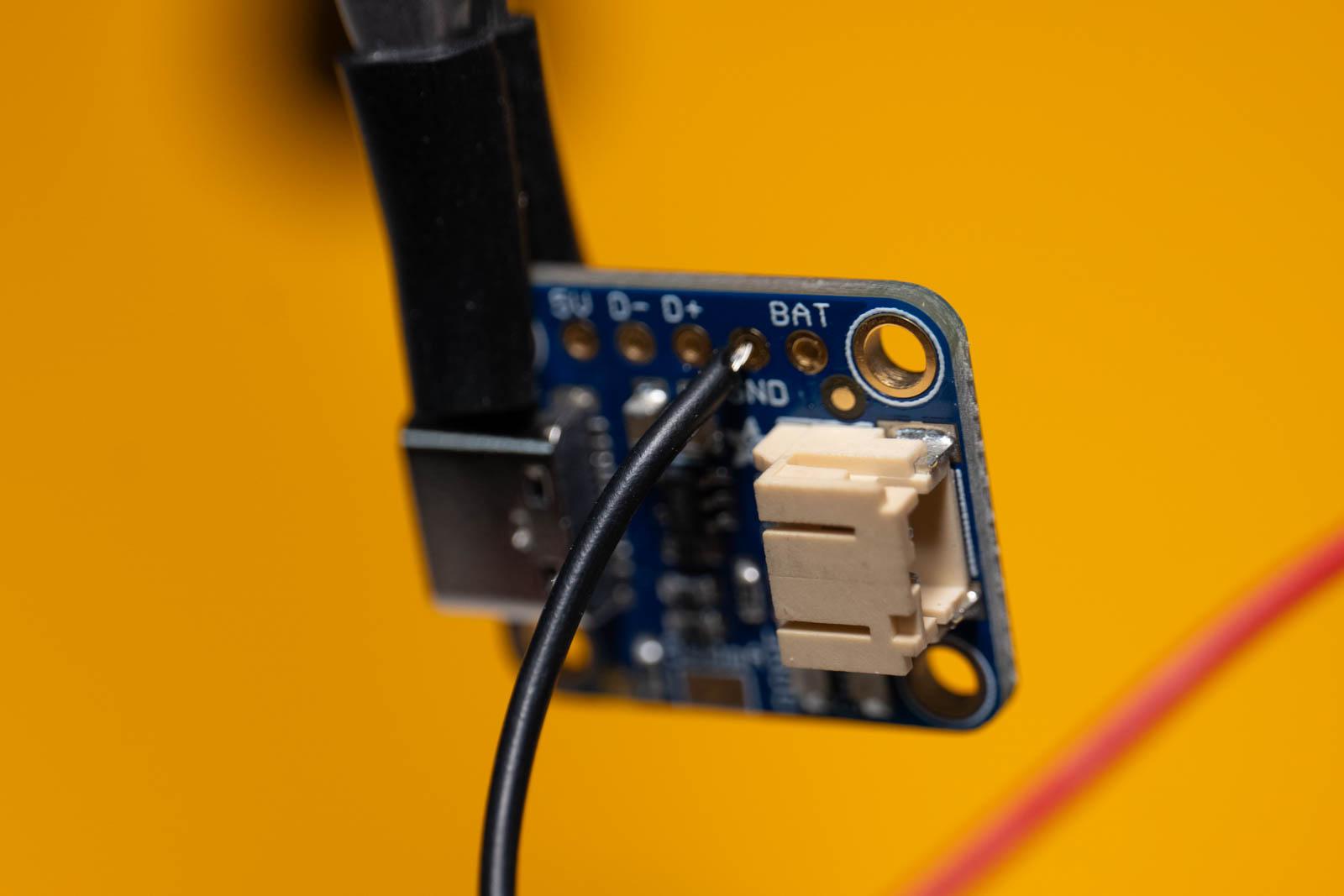

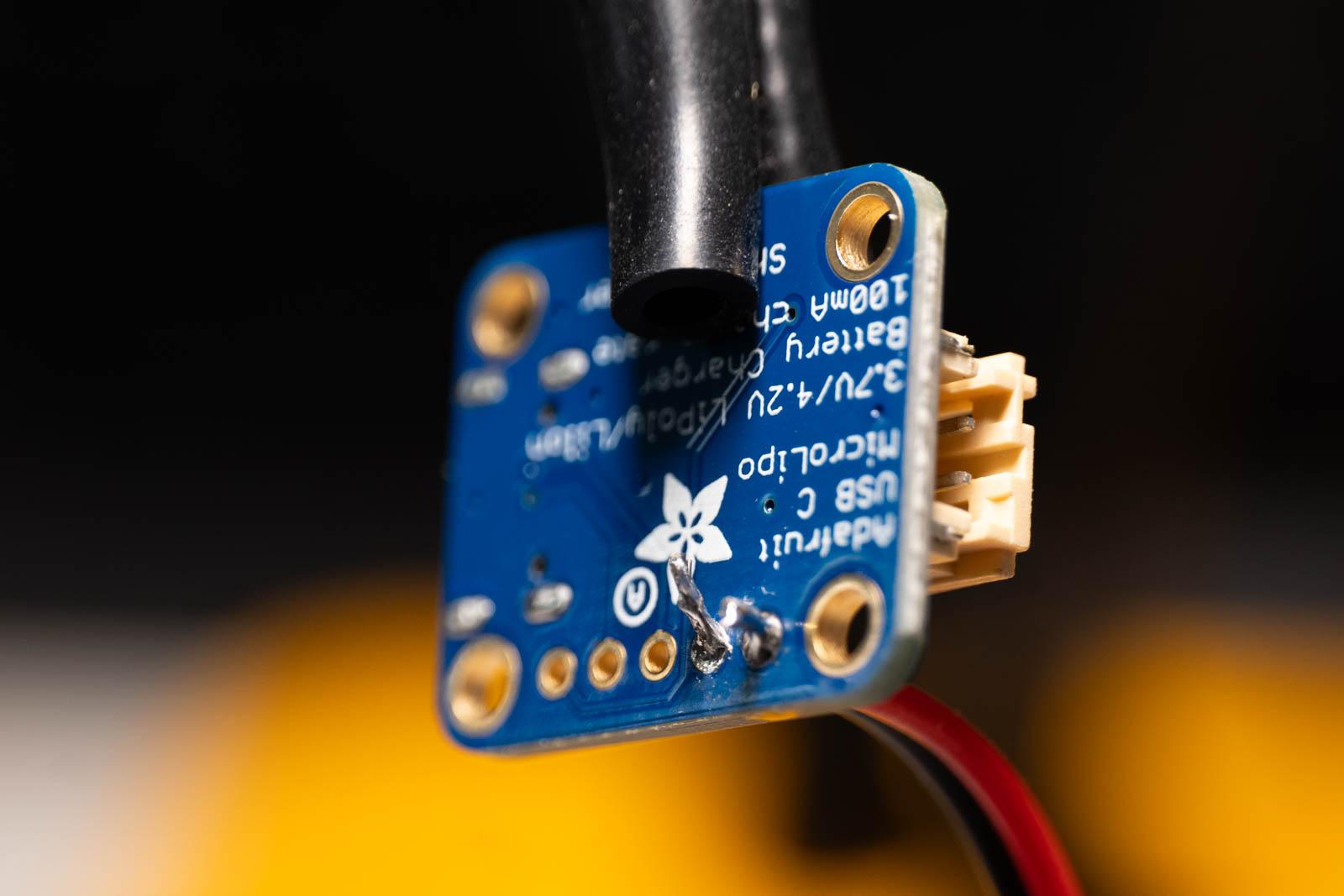


##

##

##

**Figure 21: Charge board soldering Figure 22: Completed charge board**

##

## Solder PWM board

- Pull loose ends of LED wires until the LED is just outside of the housing.
- Pull loose ends of charge board wires until the charge board is 20 mm from the core piece.
- Trim the 4 loose wires on the right to 30 mm (Figure 23).
- Cut a separate piece of red wire to 80 mm length (Figure 24).
- Strip the 4 wire ends 4 mm as well as both ends of the red 80 mm wire.
- Tin wire ends including 80 mm piece.


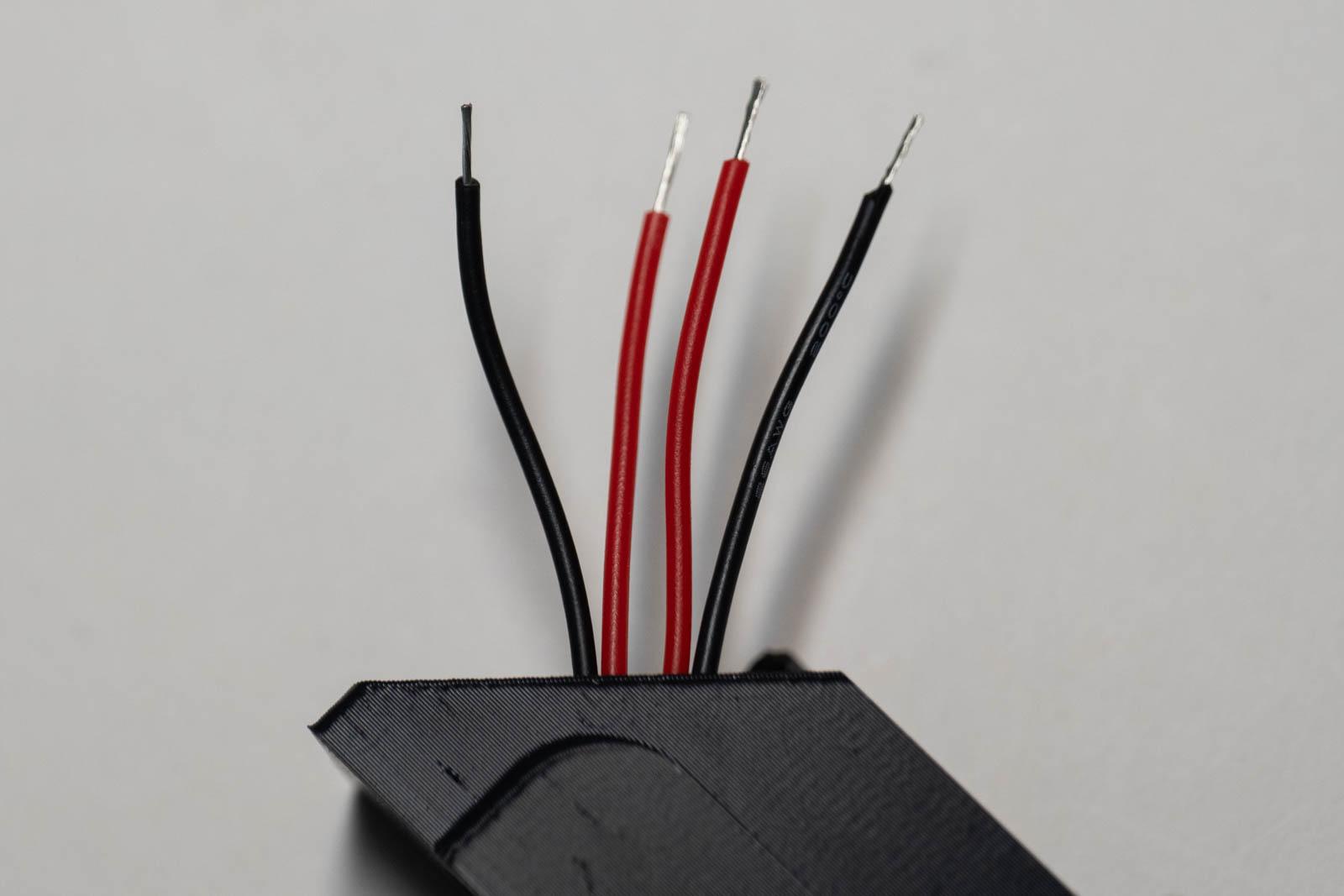

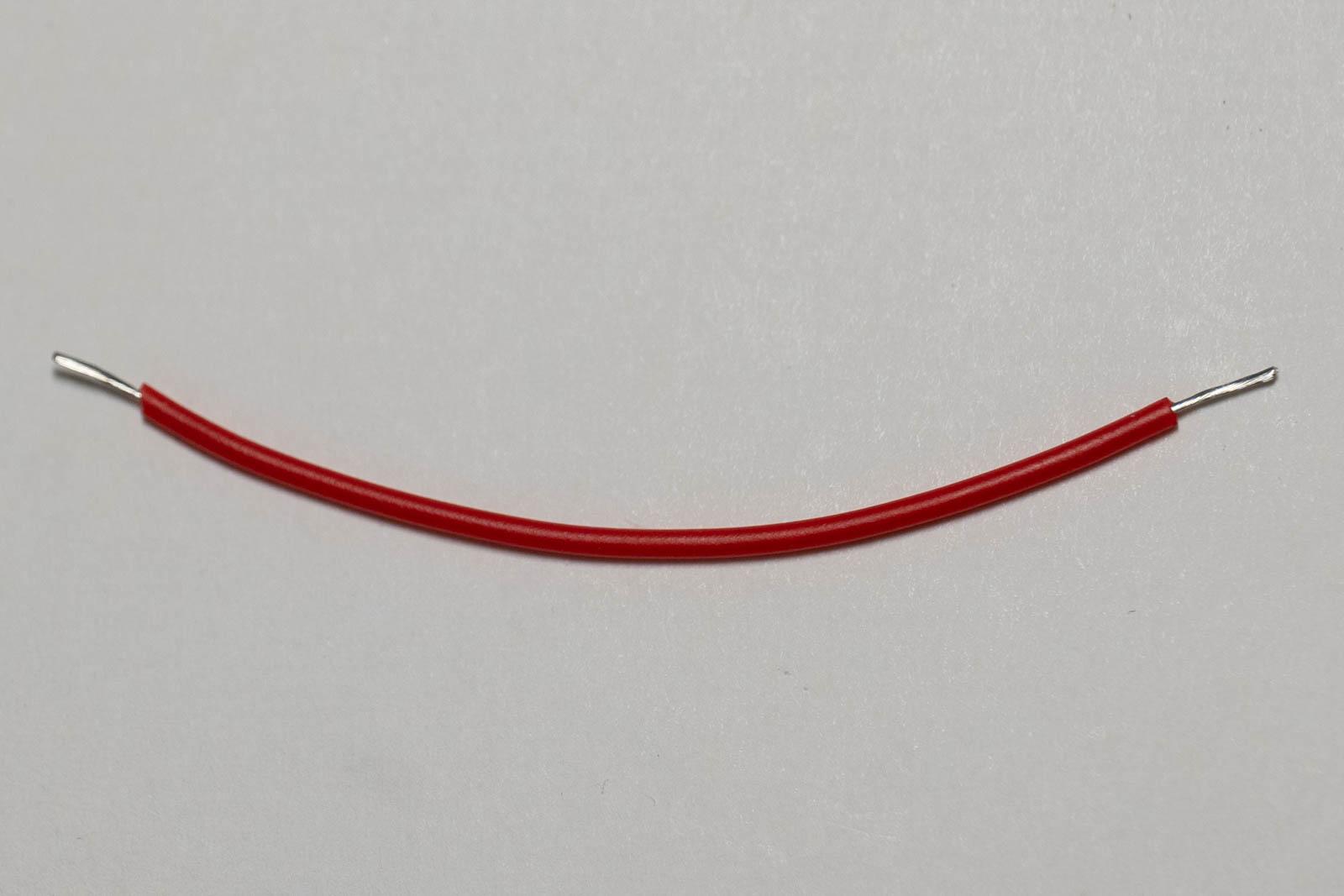


**Figure 23 Figure 24: 80 mm wire**

- Solder bottom red wire and 80 mm piece to resistor in a “Z” arrangement as close to the resistor as possible (Figure 25).
- Trim excess resistor wire.
- Bend 80 mm wire as shown in Figure 26.
- Cut 5 mm outer diameter heat shrink to 15 mm length and slide over resistor.


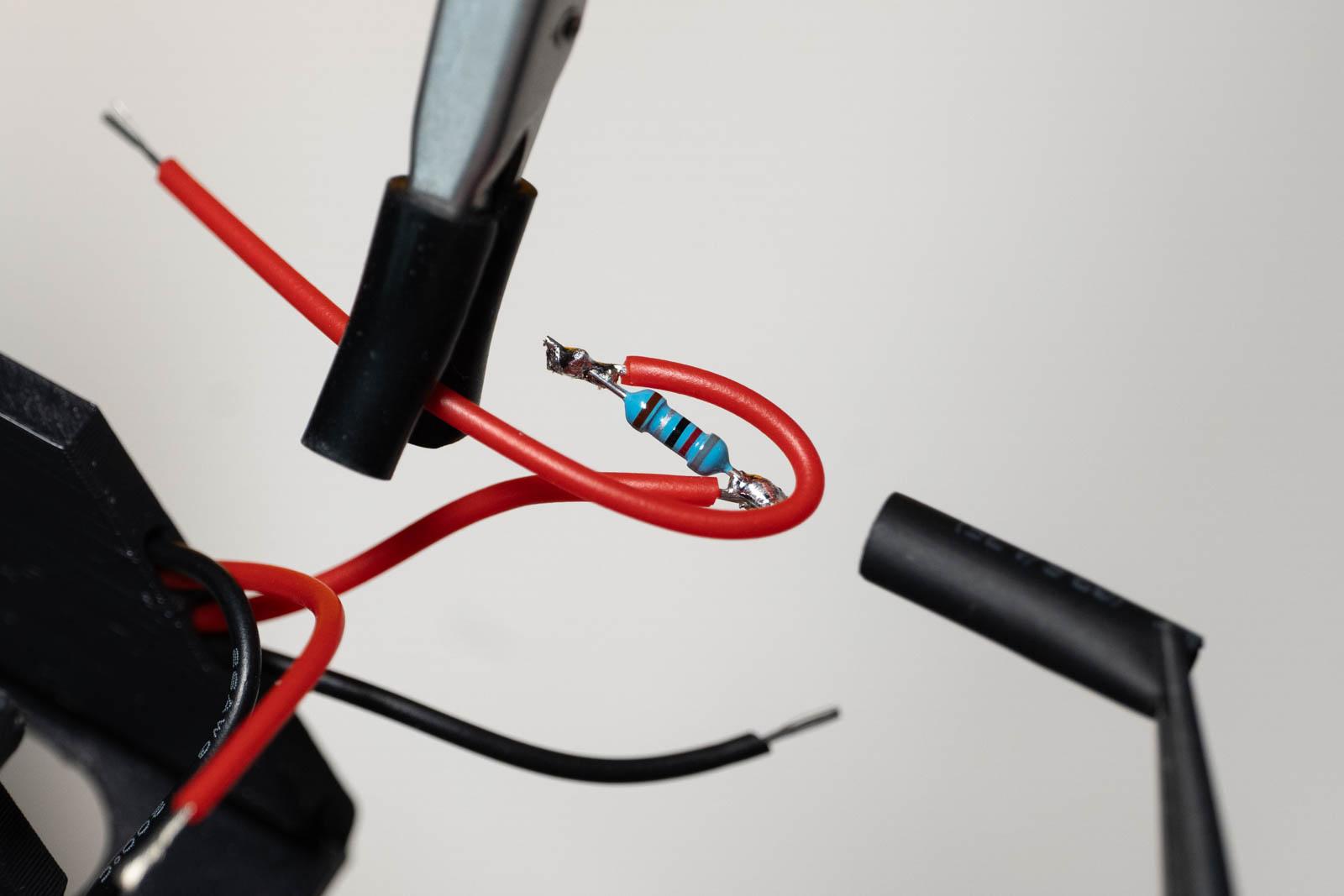

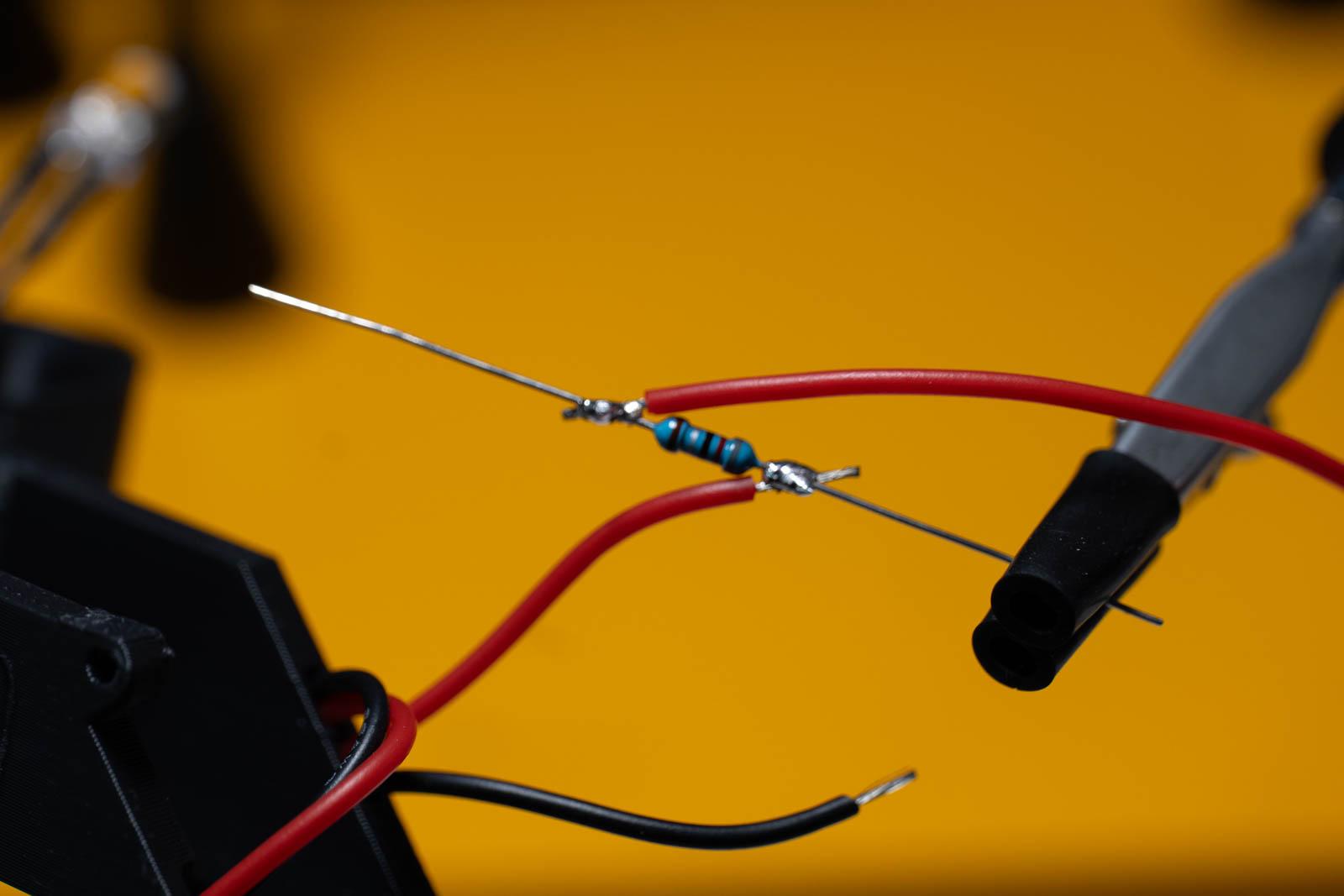


**Figure 25: Soldering resistor Figure 26: Heat shrink wrapping resistor**

- Tin the four PWM terminals (Figure 27).
- Solder wires to PWM by heating PWM solder and sliding wire through hole (Figure 28).
  - Far right black to “LED-”
  - Resistor to “LED+”
  - Remaining red to “V+”
  - Remaining black to “V-”


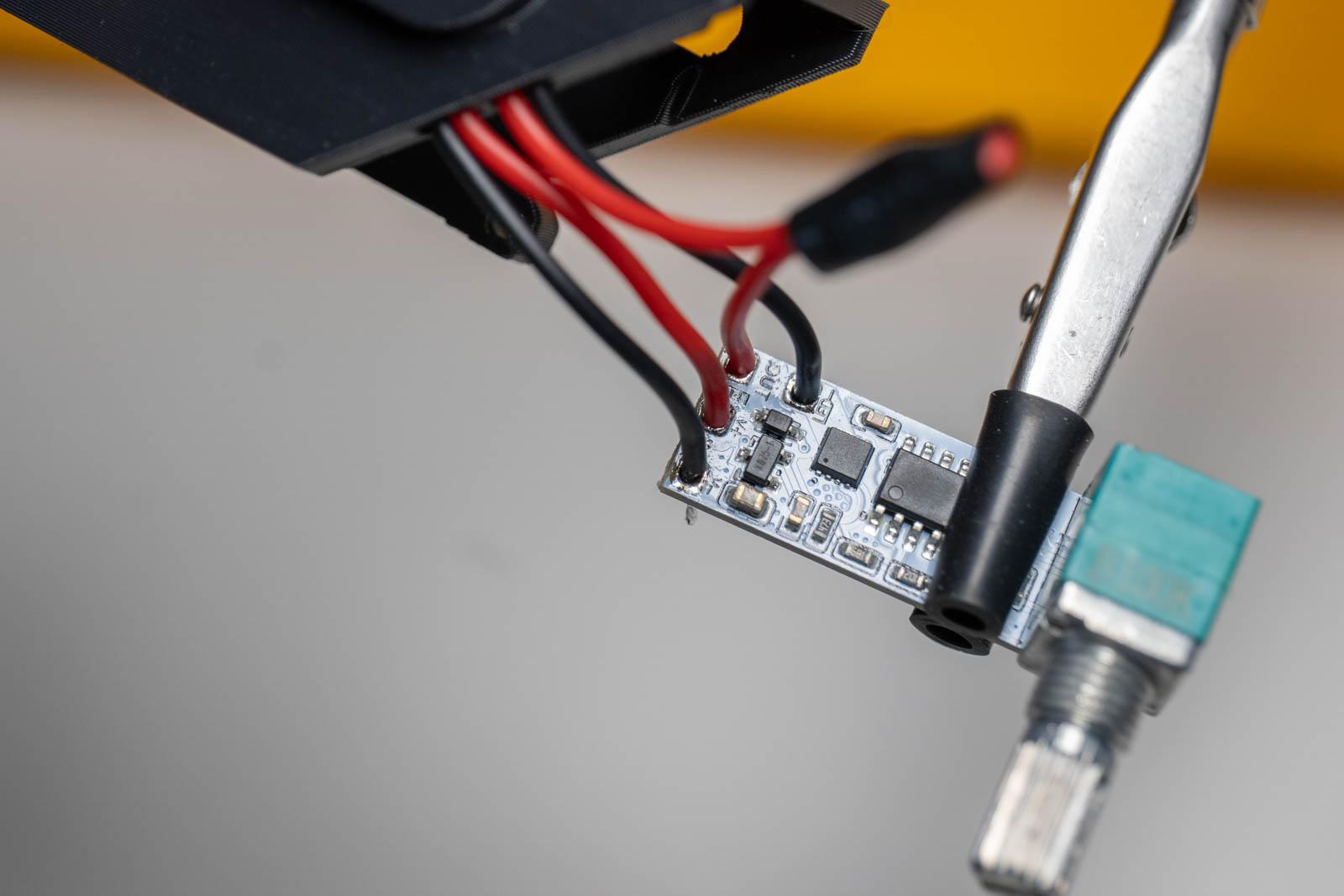

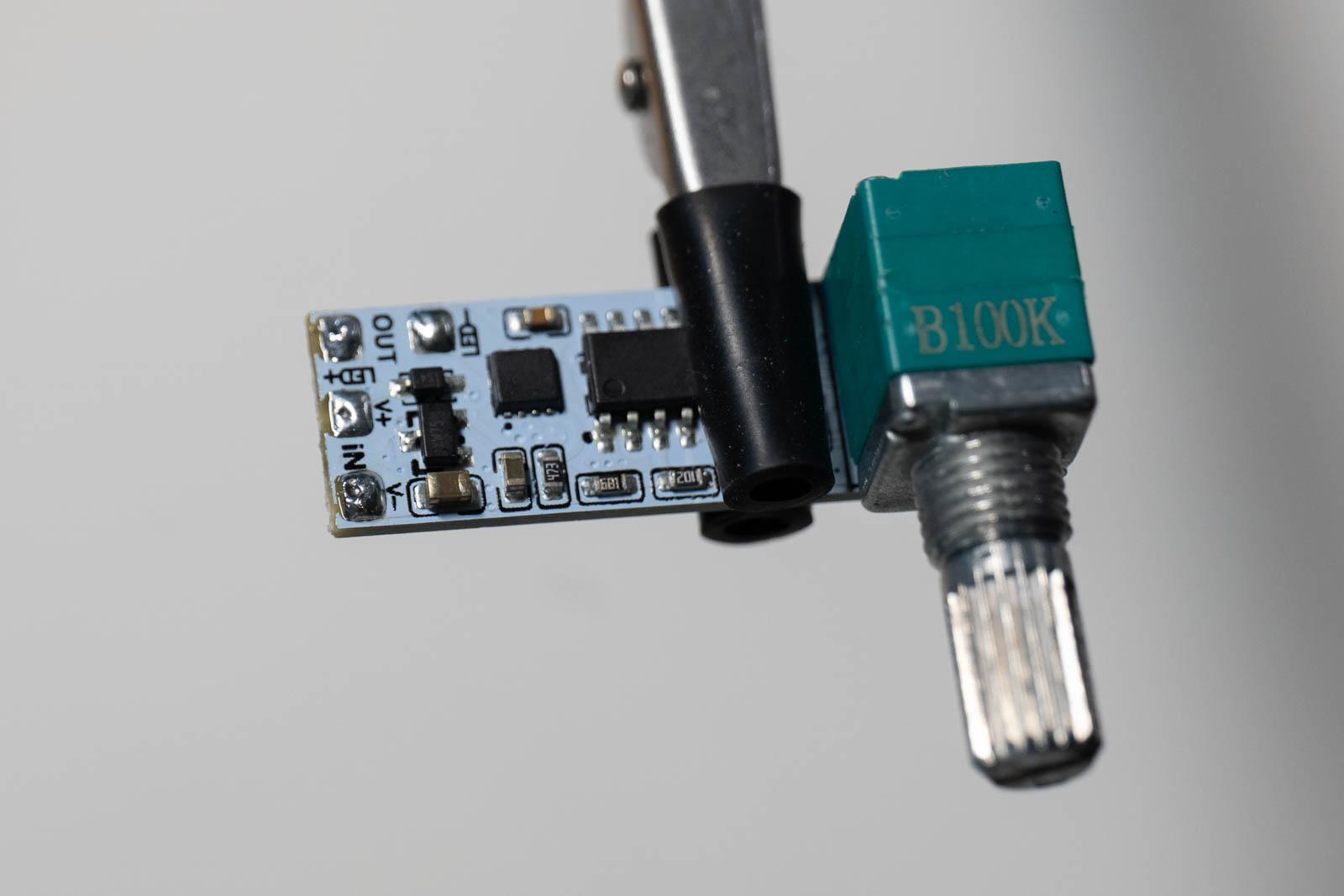


**Figure 27: PWM tinned Figure 28: PWM soldered**

- Plug in battery to JST port and check circuit works then remove battery (Figure 29).
- Trim excess wire on back of PWM (Figure 30). It is ok to leave small tails as there is space.
- Optional: apply silicone paste around the 4 solder points on the PWM and 2 solder points on the charge board.


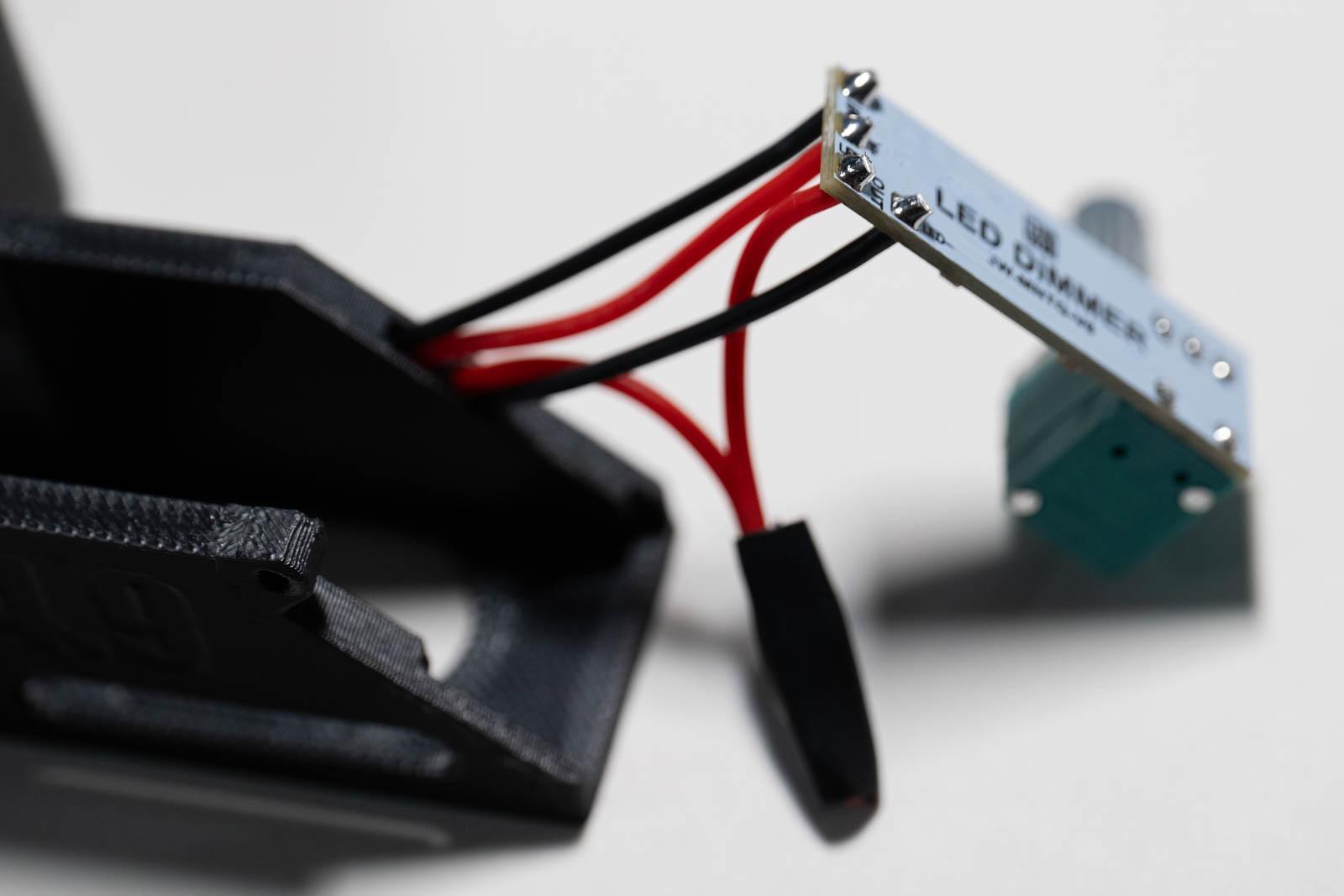

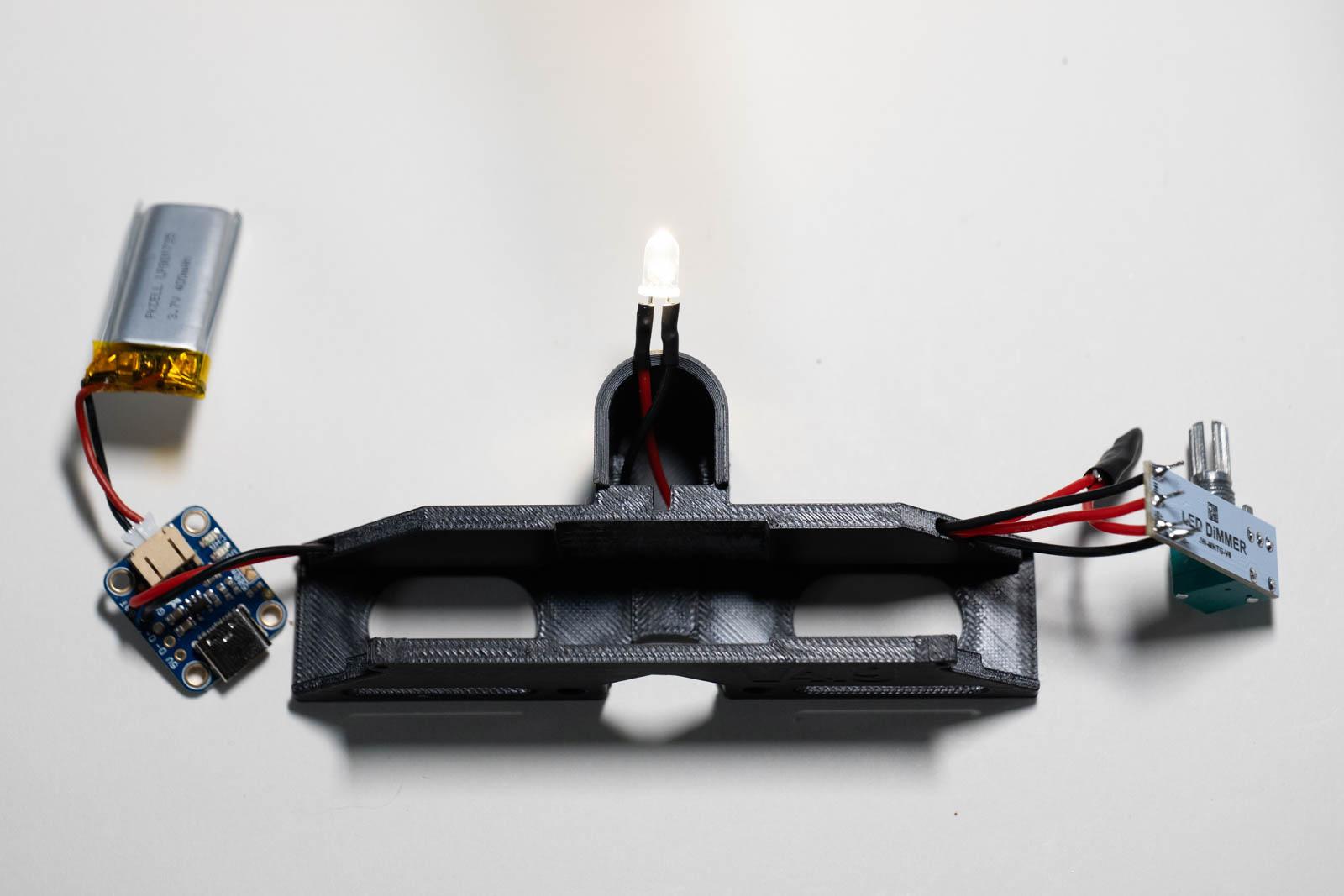


##

##

##

**Figure 29: Circuit test Figure 30: PWM wires trimmed**

##

## LED holder assembly

- Use a lighter to remove any stringing inside the TPU LED piece
- Install SK6 diaphragm with the blade side towards LED/posterior. Aim for the sliding range to be towards the left (Figure 31). Use the handle of a small screwdriver or similar object to press it into place.
- Use a small flathead screwdriver to move the screw hole for the lever into view (Figure 32).
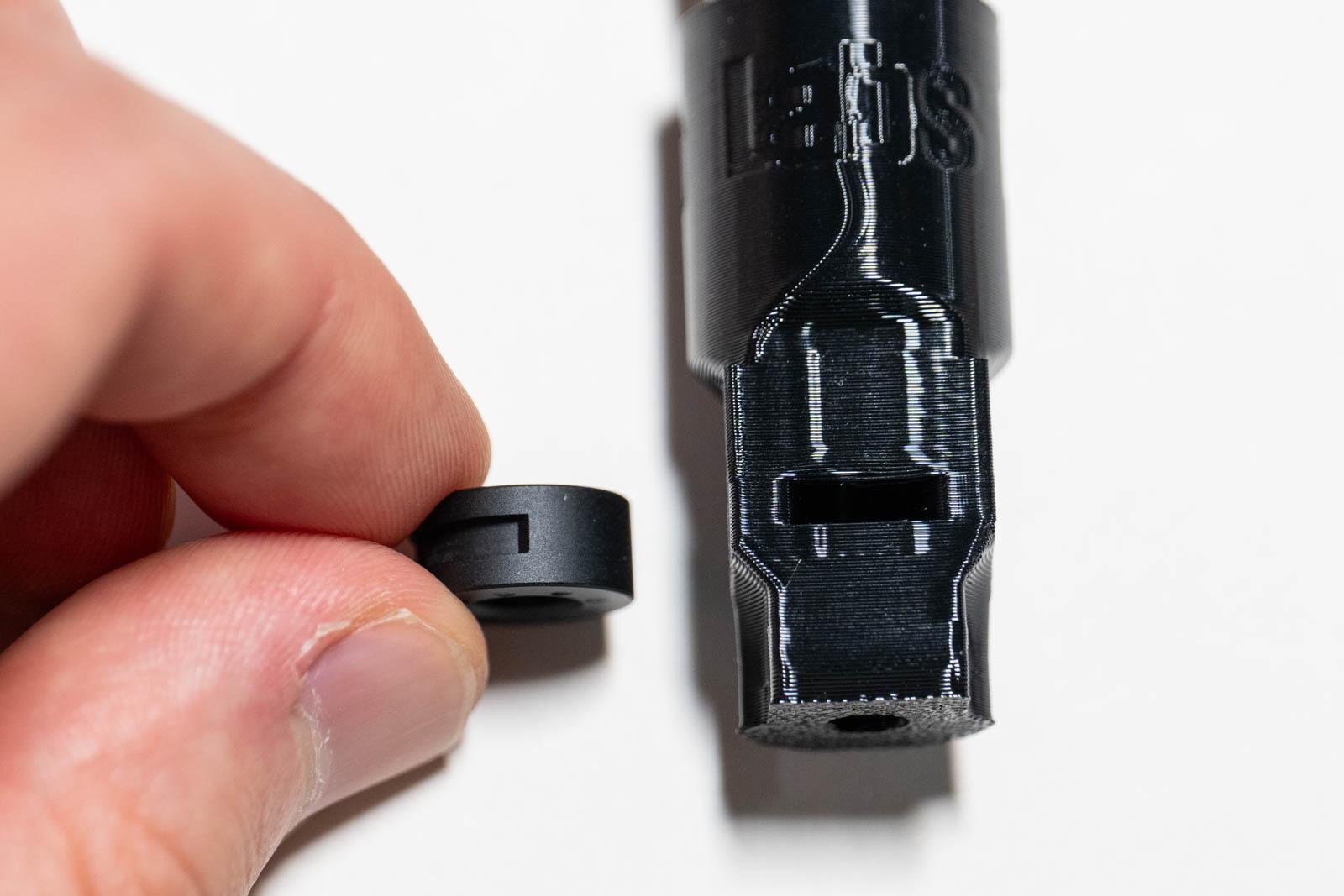

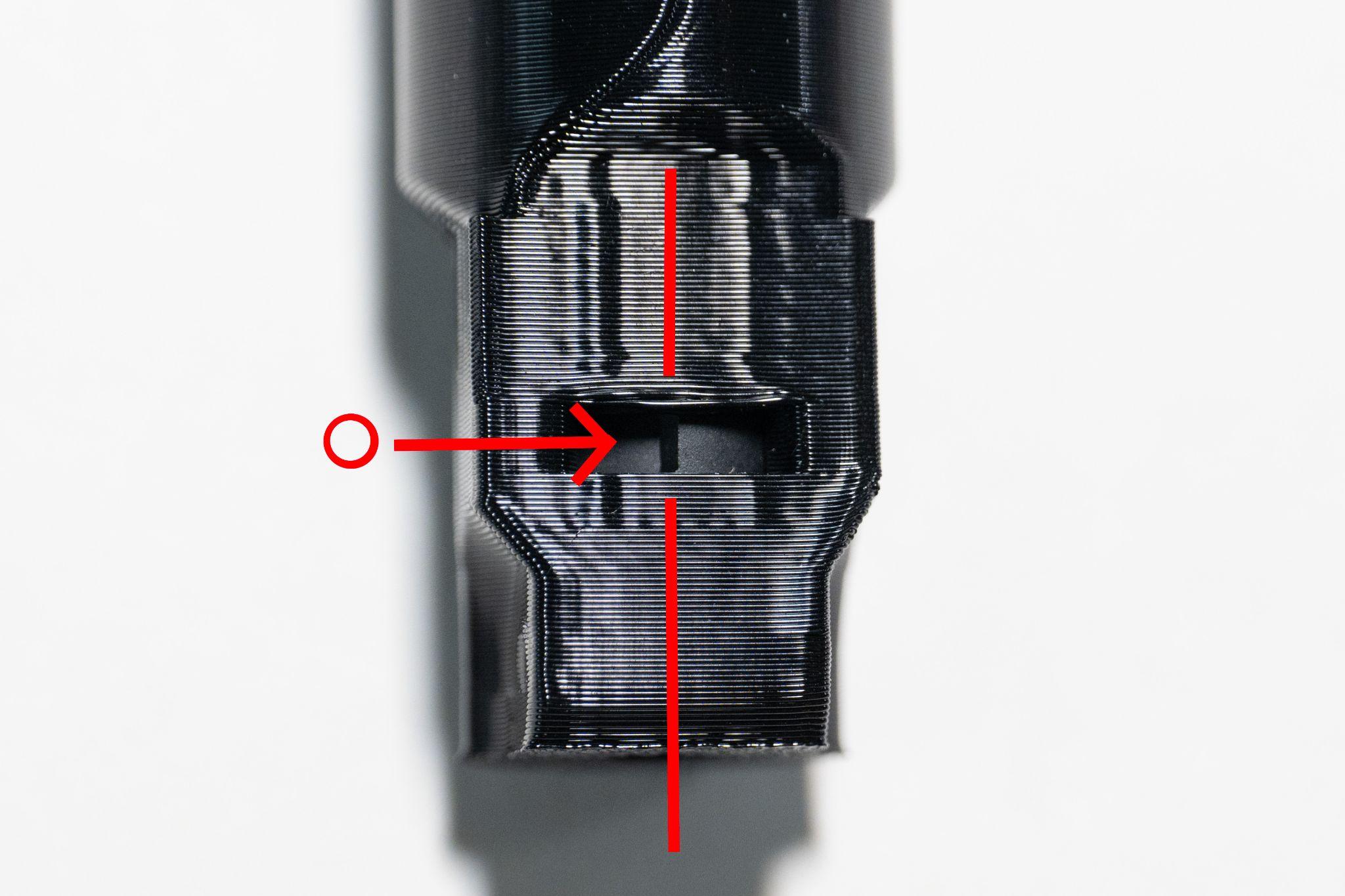


**Figure 31: Diaphragm alignment Figure 32: Screw hole alignment**

- Install the diaphragm lever (Figure 33). Make sure the lever is 7 mm as some come with a 3.5 mm lever that is not long enough.
- Slide the diaphragm lever to the right past the end of its range as far as possible so it rotates the entire diaphragm within the TPU piece (Figure 34). This step is critical as it sets the range of the spotlight size.
- Press plano-convex lens into LED assembly with the convex surface facing out (Figure 35).

**Figure 33: Lever installed Figure 34: Repositioning Figure 35: Lens installed**
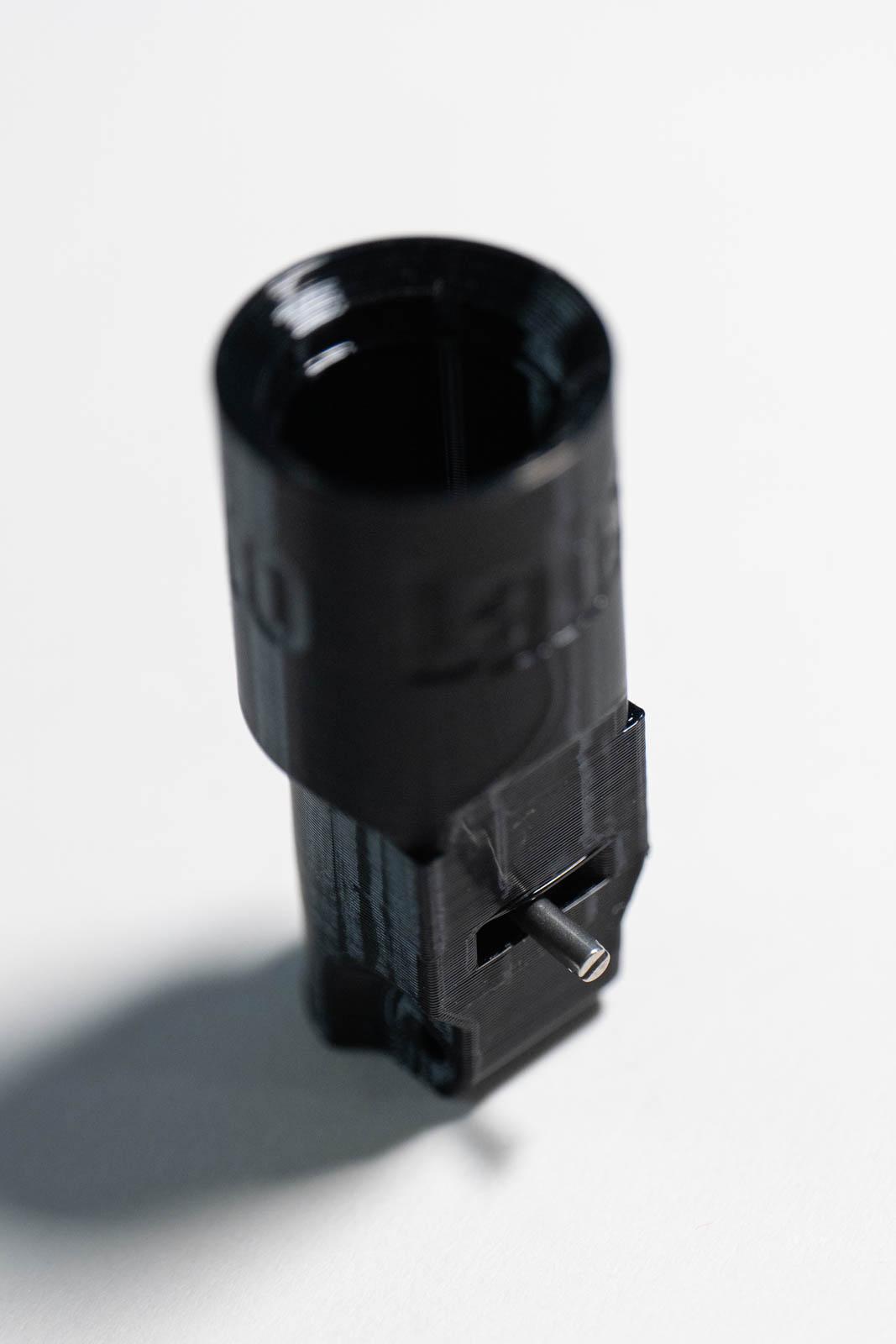

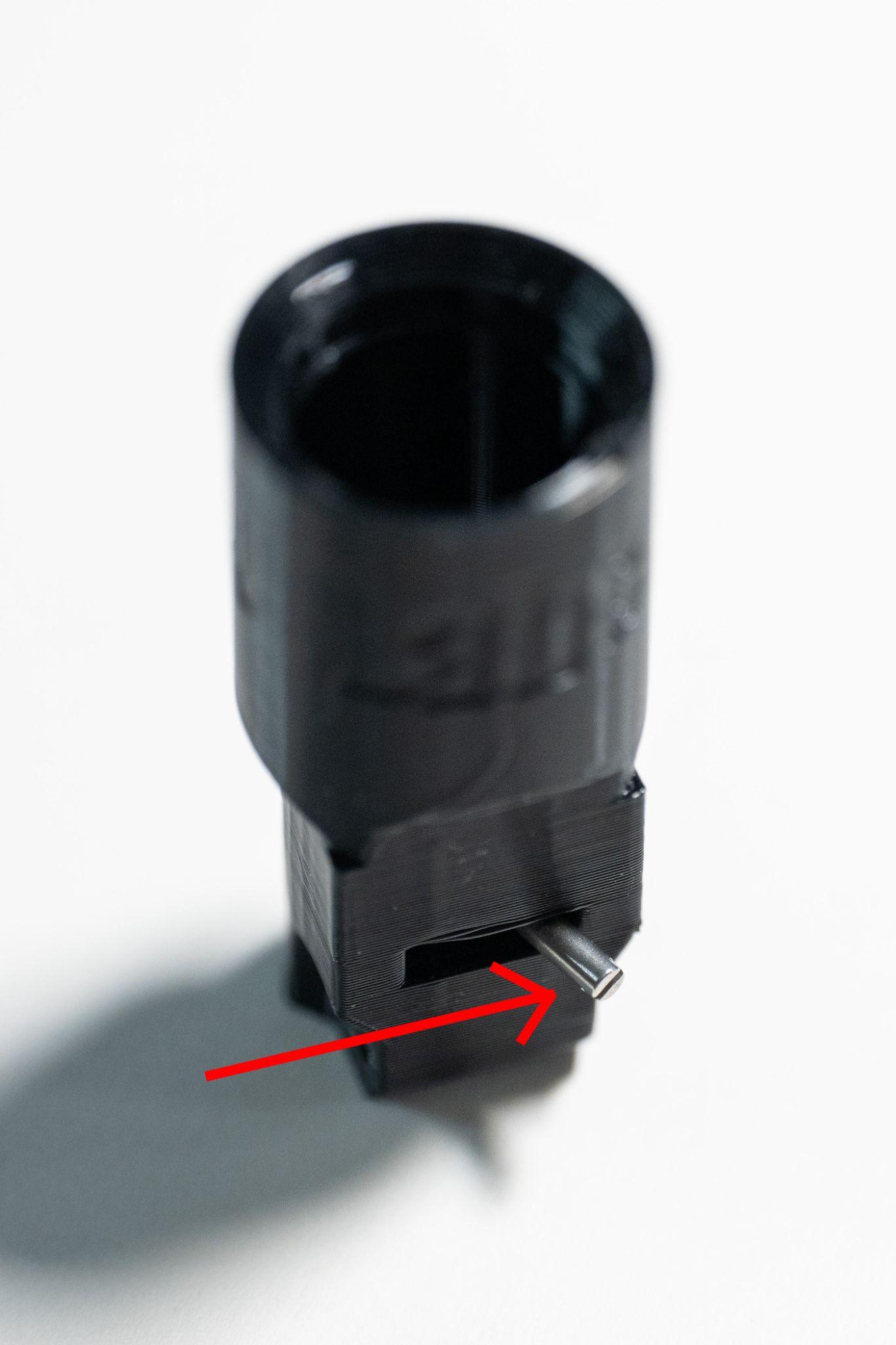

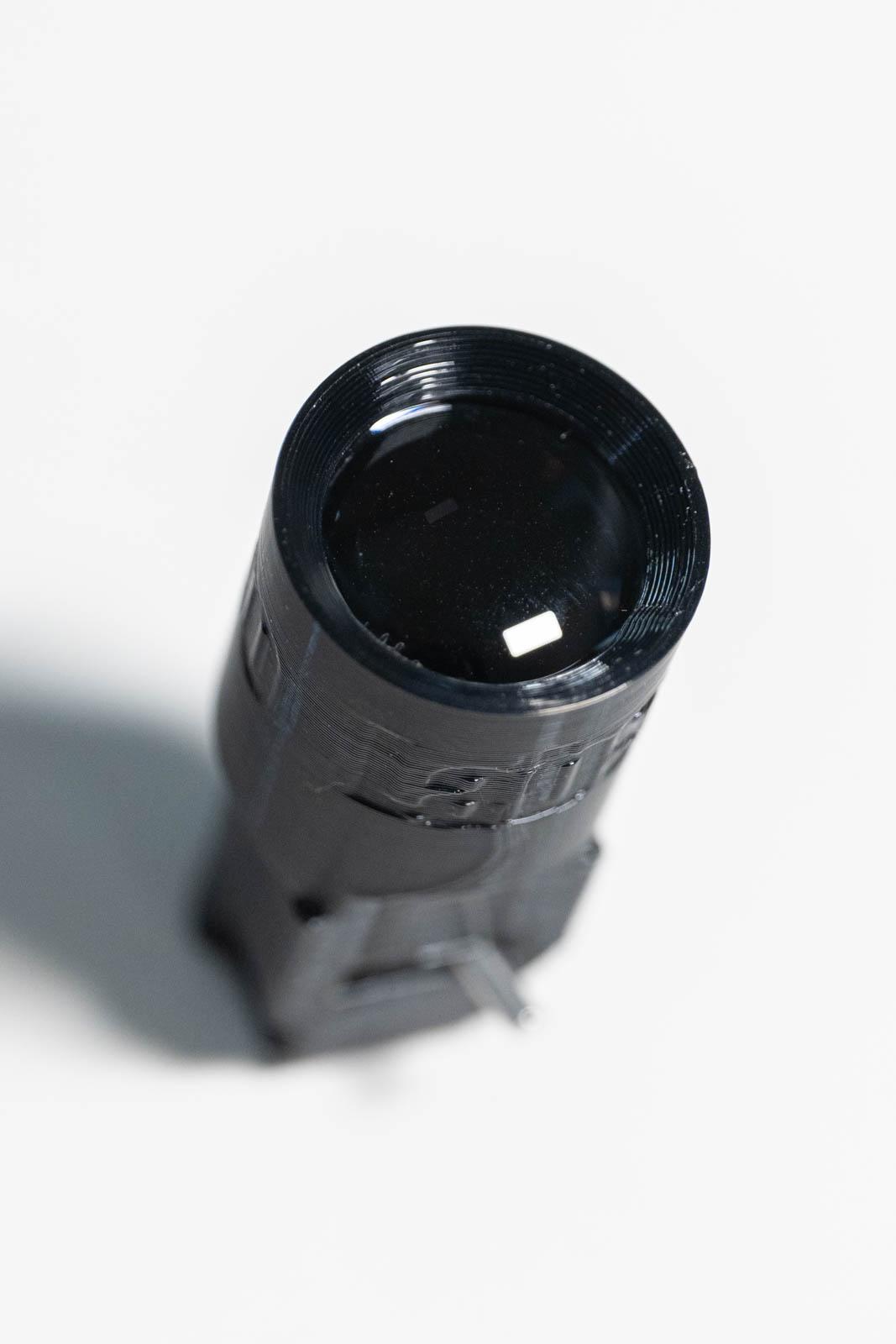


- Press LED into TPU piece and bend LED leads slightly as shown in Figure 36.
- Install printed diaphragm slider into core with the grooves in each aligned together. Optional: adding a small amount of silicone grease to the inferior surface of the diaphragm lever can provide smoother lever operation.
- Press TPU piece into core. Make sure the diaphragm lever is centered and install through the grooves in the core and diaphragm slider (Figure 37). Simultaneously pulling the LED wires on the PWM side helps prevent wires from bunching up inside the LED housing.


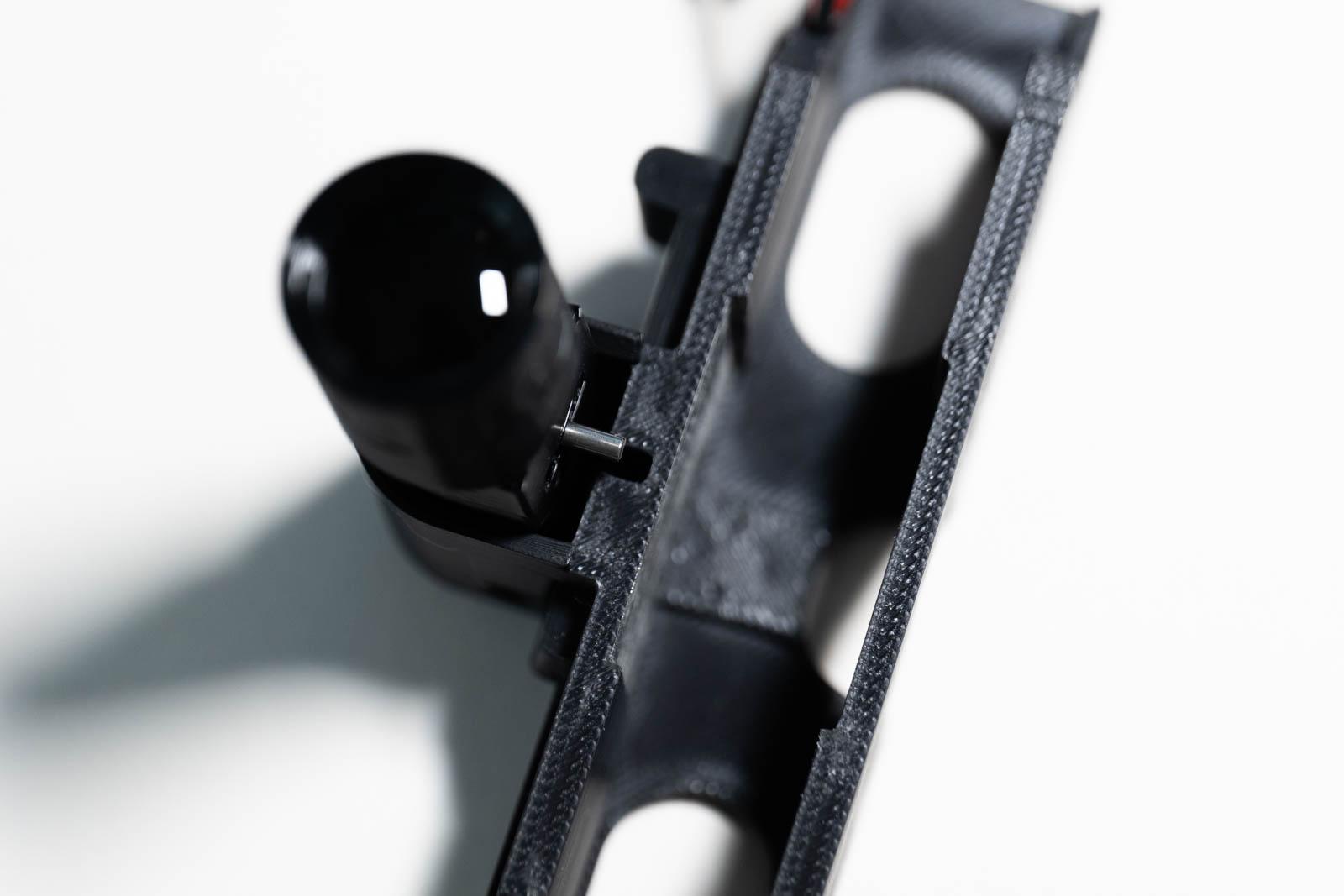

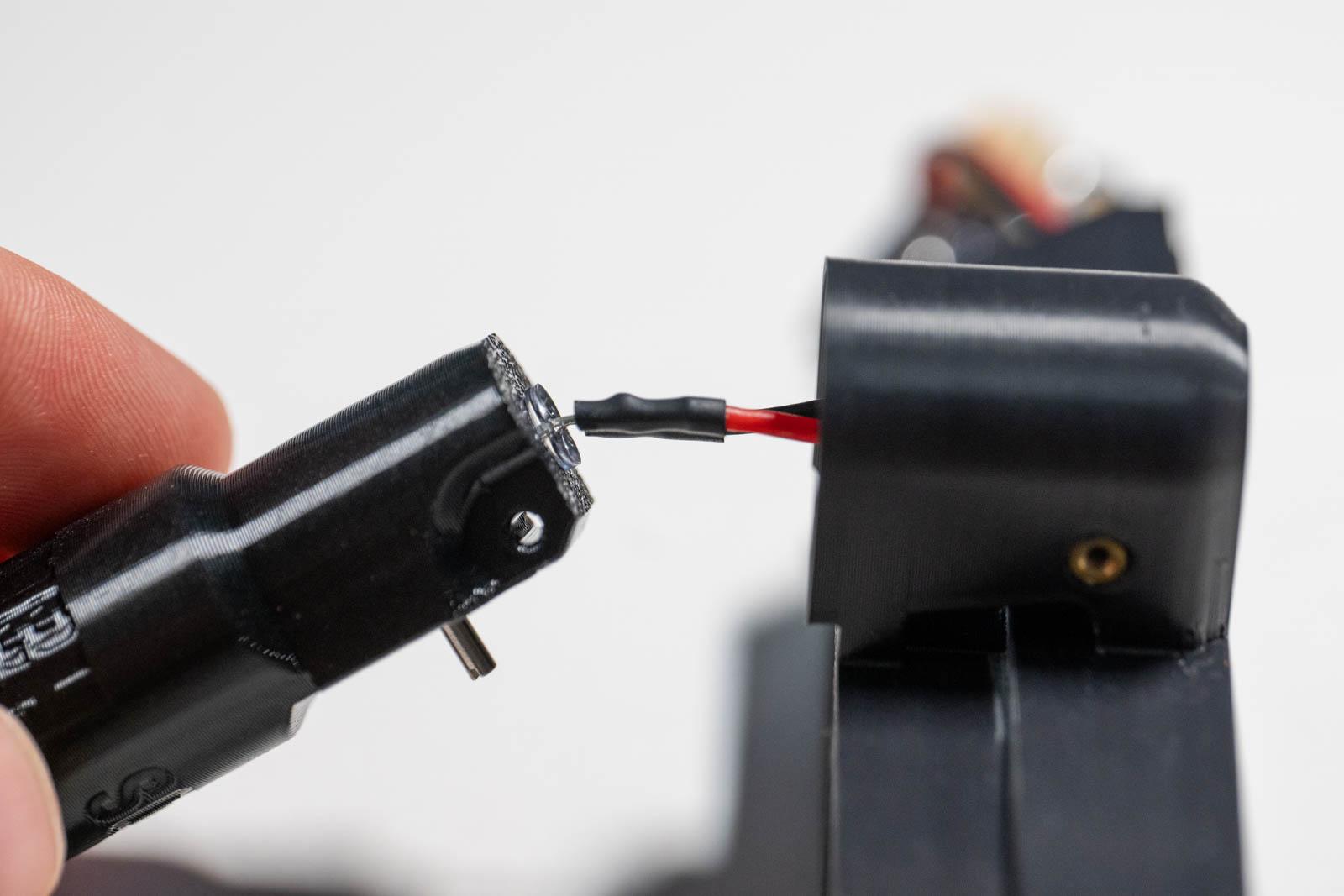


**Figure 36: Bend LED leads as shown Figure 37: LED unit install**

- Screw M2 18 mm screw through core and LED unit (Figure 38).
- Install center mirrors and side mirrors (Figure 39).


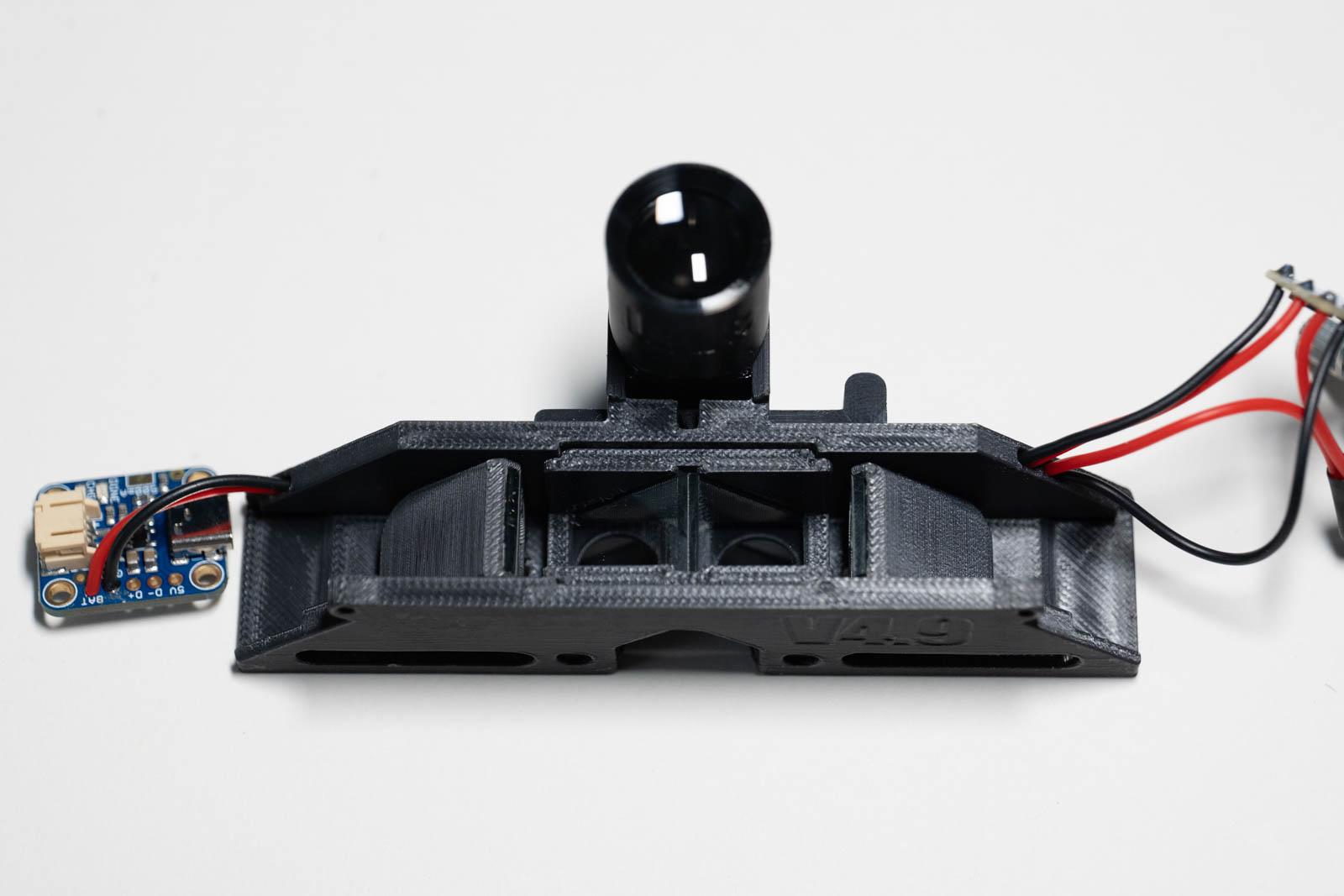

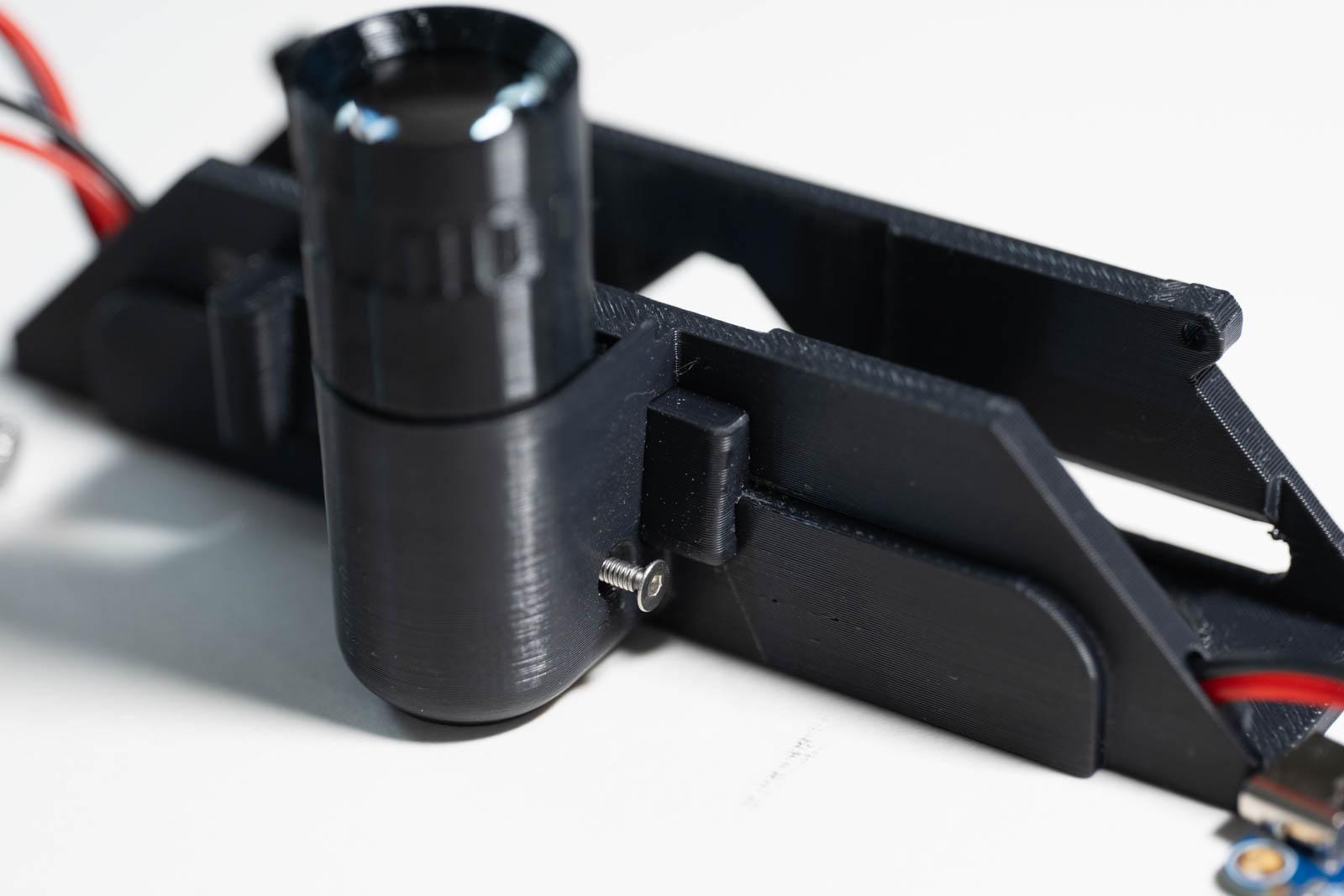


#

#

**Figure 38: LED hinge screw install Figure 39: Mirrors installed in core**

# Frame details

## Polycarbonate window

- Mark polycarbonate sheet using printed template.
- Use Lexan scissors or X-ACTO knife to cut out polycarbonate.
- Remove protective film from polycarbonate.
- Apply a conservative amount of cyanoacrylate glue to recess in frame and carefully install polycarbonate piece exposed side down as shown in Figure 40. The edge of the previously used template can be used to press the polycarbonate into place.
- Once dry, remove protective film from the remaining side.


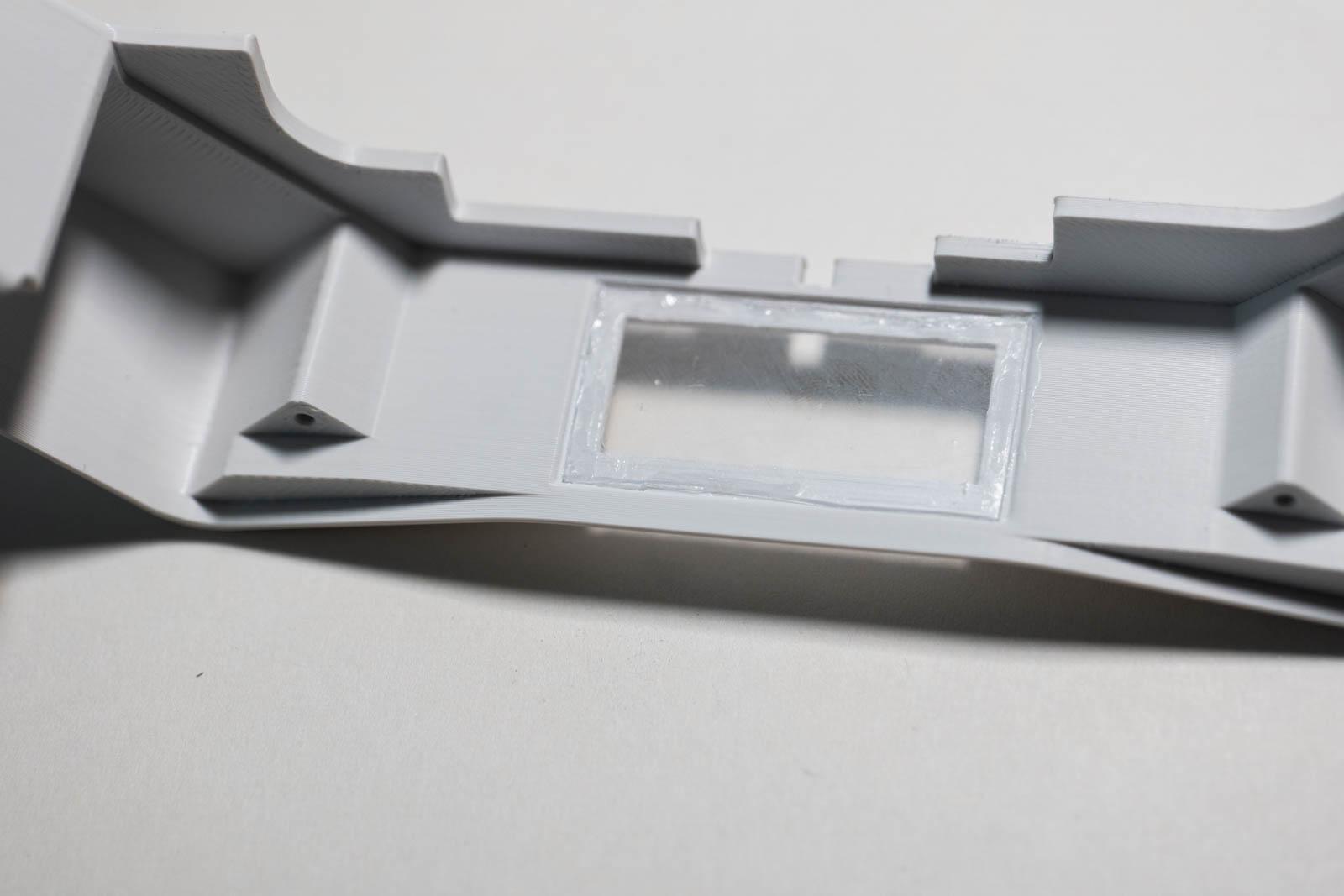


**Figure 40: Polycarbonate window placement**

##

## Charge plug

- Remove supports from TPU charge plug
- Install in frame as shown in Figure 41.

## Temple install (2x)

- Use an M2 18 mm screw to attach the temple piece to the frame as shown in Figure 42.
- Repeat on the other side.


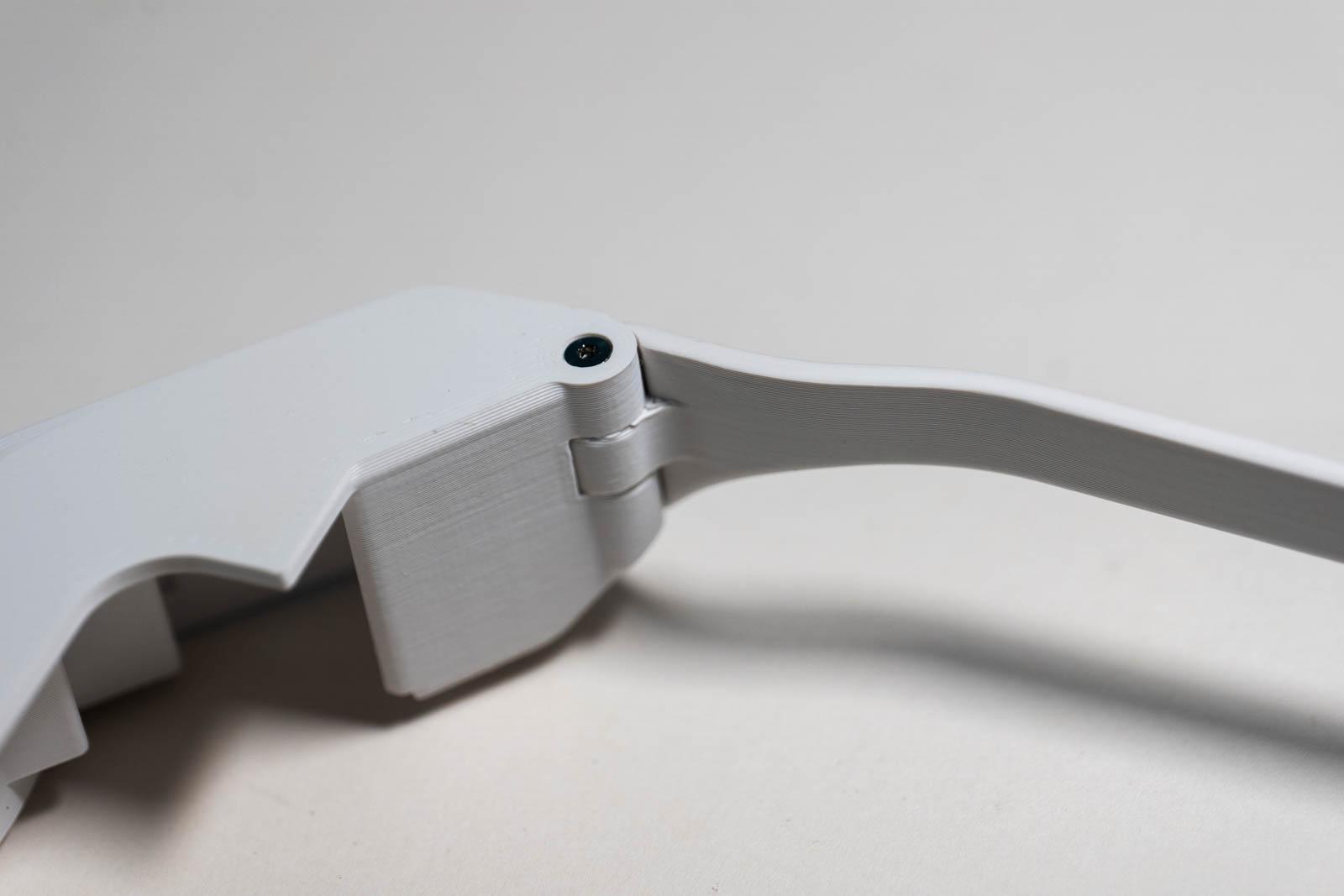

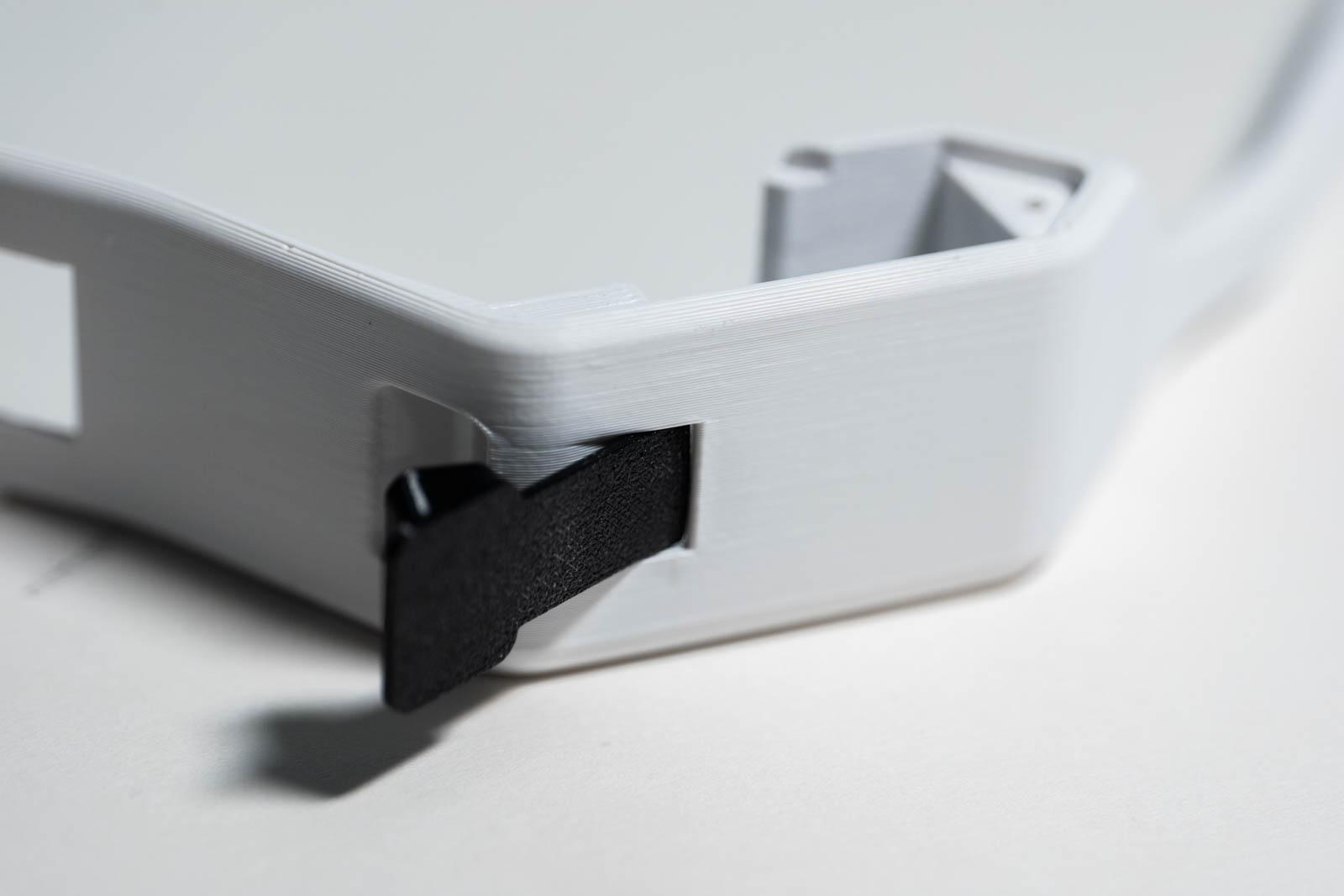


**Figure 41: Charge plug installed** **Figure 42: Right temple installed**

# Back plate charge indicator

- Apply cyanoacrylate glue to “U-shaped” recess in back plate.
- Press or slide transparent PETG charge indicator piece into place (Figure 43). Note: It is ok to wipe off excess glue from the rough side as it will be hidden inside.


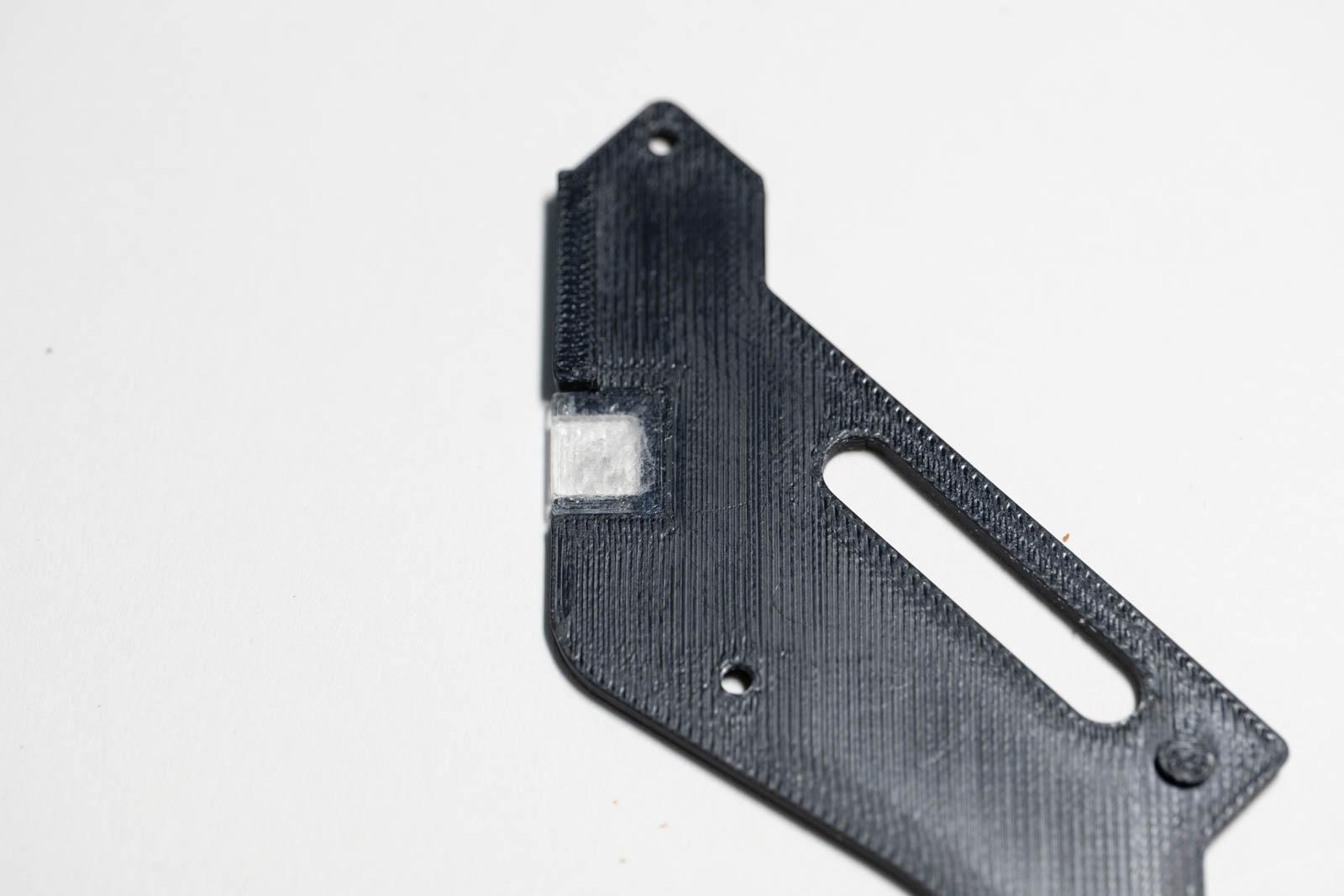


**Figure 43: Charge indicator installed**

#

# Final assembly

- Place core assembly into frame (Figures 44 and 45). Ensure wires are not pinched between core and frame.


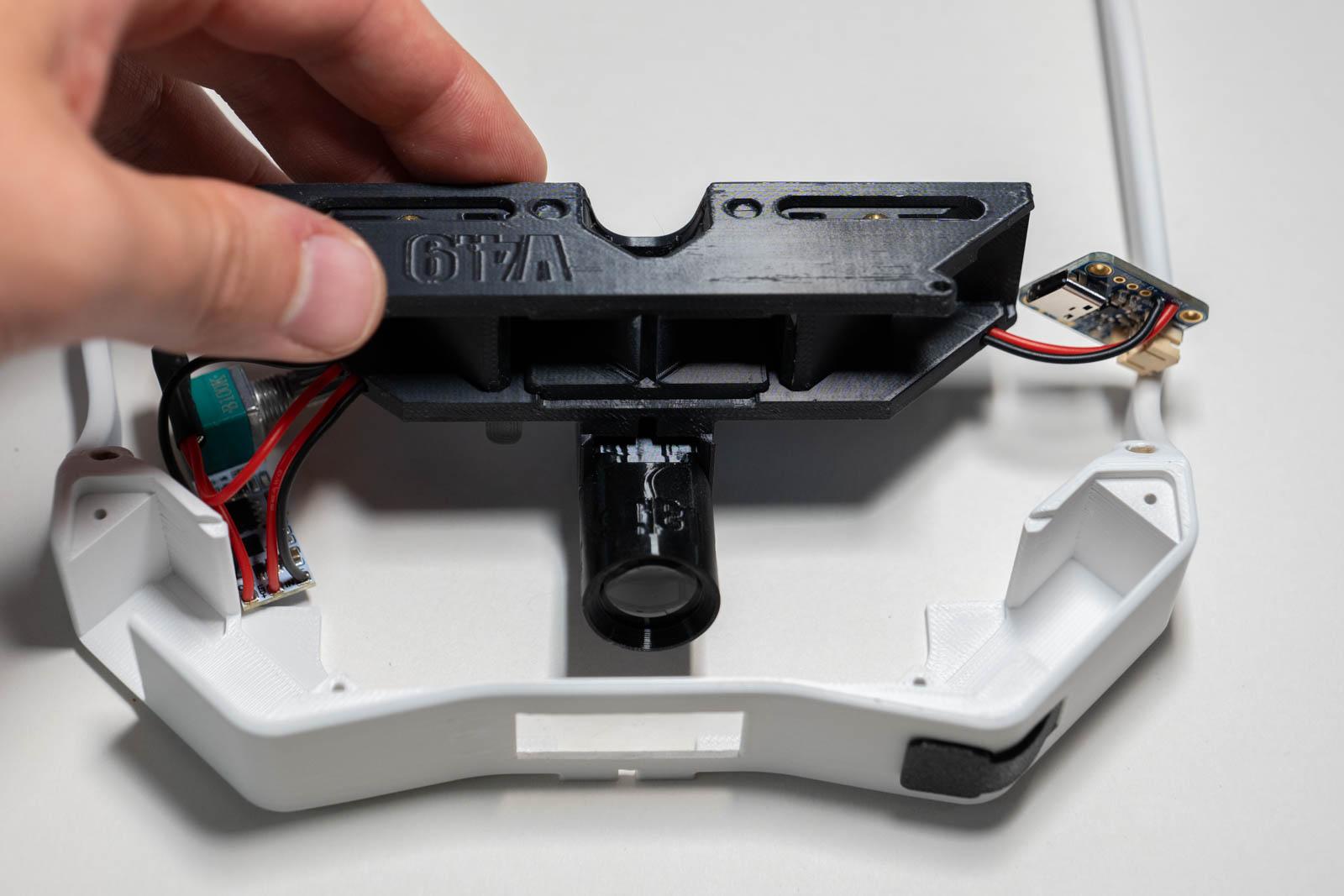

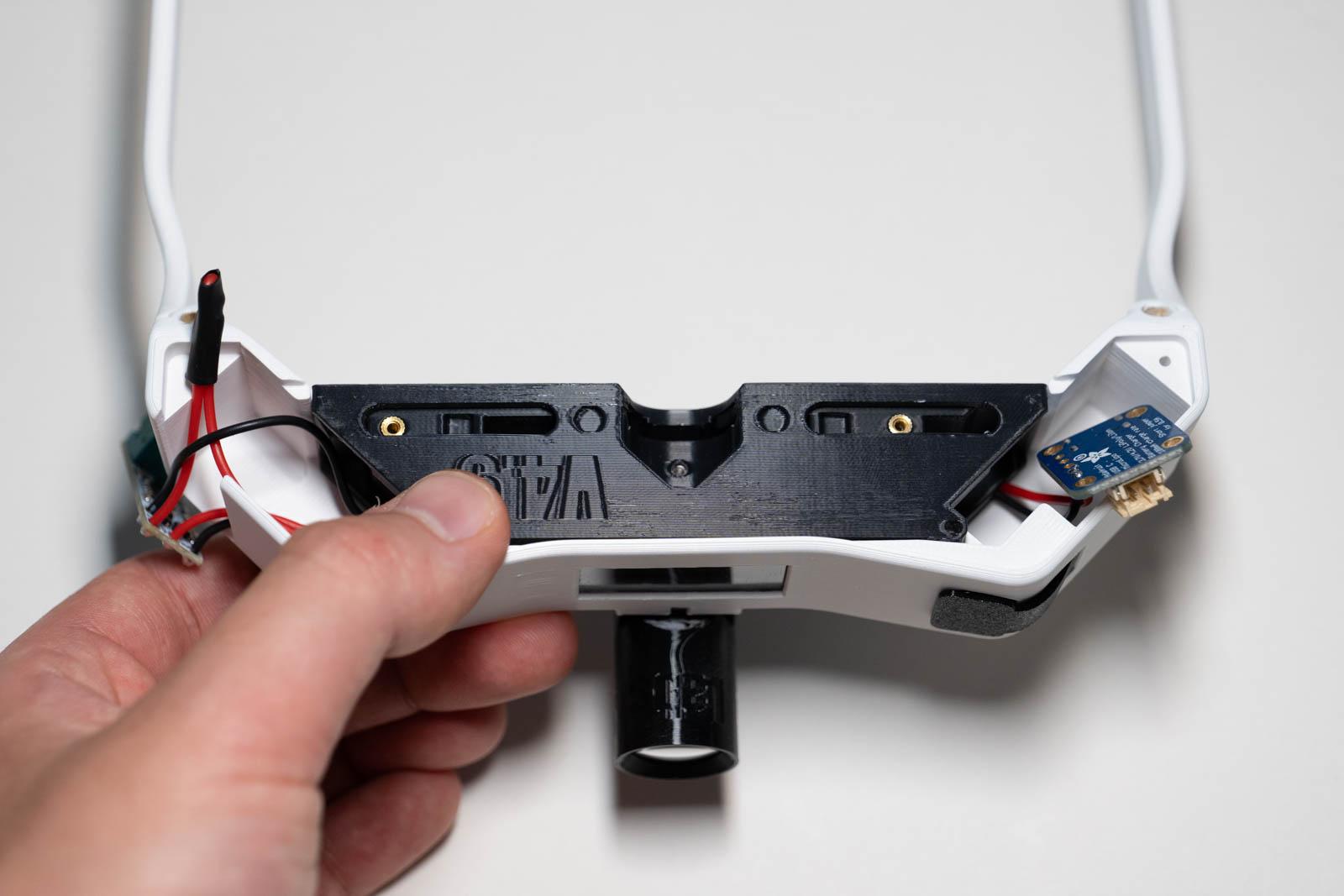


**Figure 44: Core installing into frame Figure 45: Core positioned in frame**

## Charge board install

- Use tweezer pliers to install charge board into frame (Figures 46 and 47). Make sure the solder joints on the charge board are trimmed flush, and make sure the TPU charge cover is closed when installing. Hold the charge board with tweezer nose pliers at the gold hole near the JST connector.


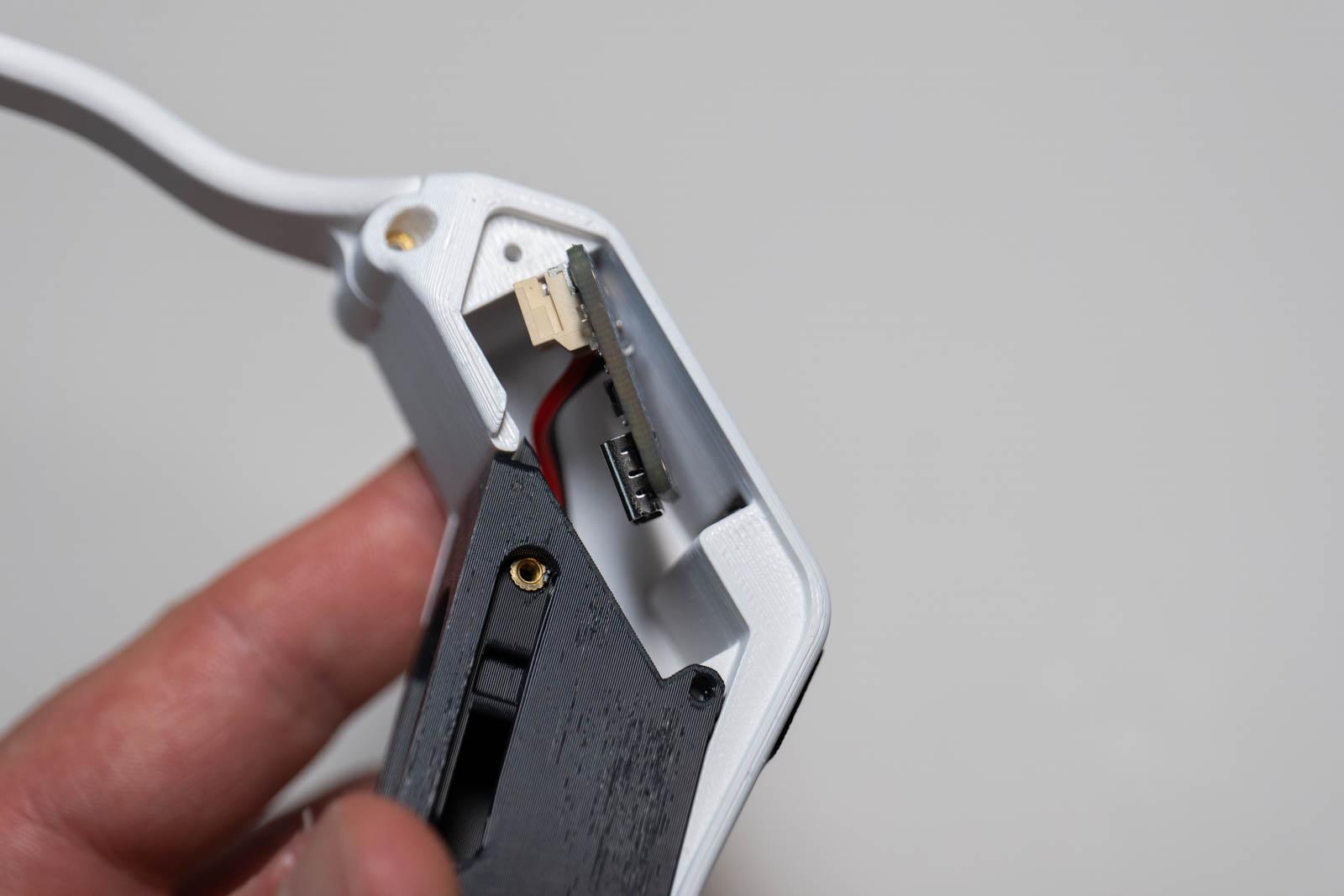

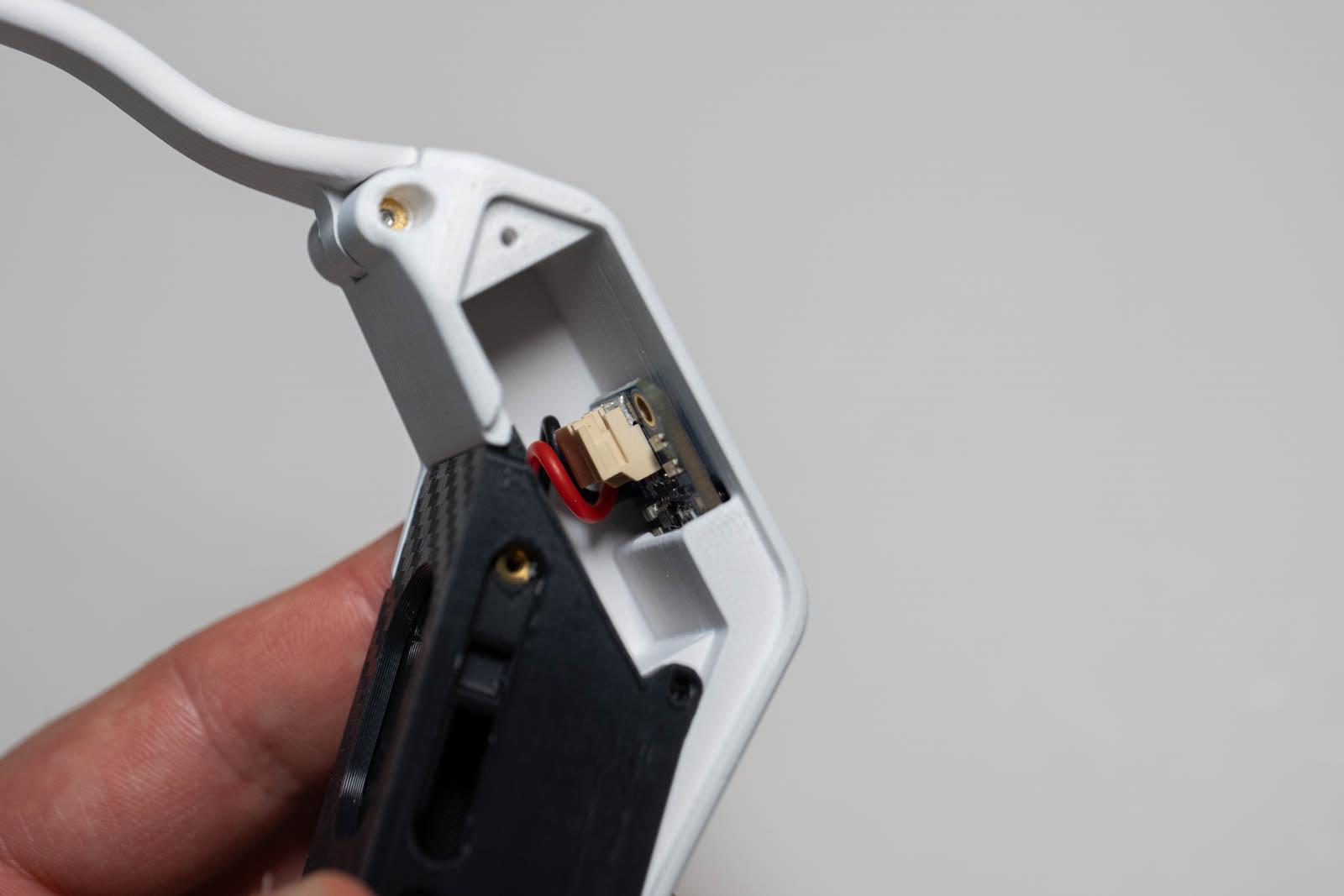


**Figure 46: Charge board install Figure 47: Charge board installed**

- Make sure USB-C port is flush with frame as seen in Figure 48.
- To remove the wire slack between the charge board and core (refer to Figure 47), use tweezer pliers to pull the two PWM wires shown in figure 49.


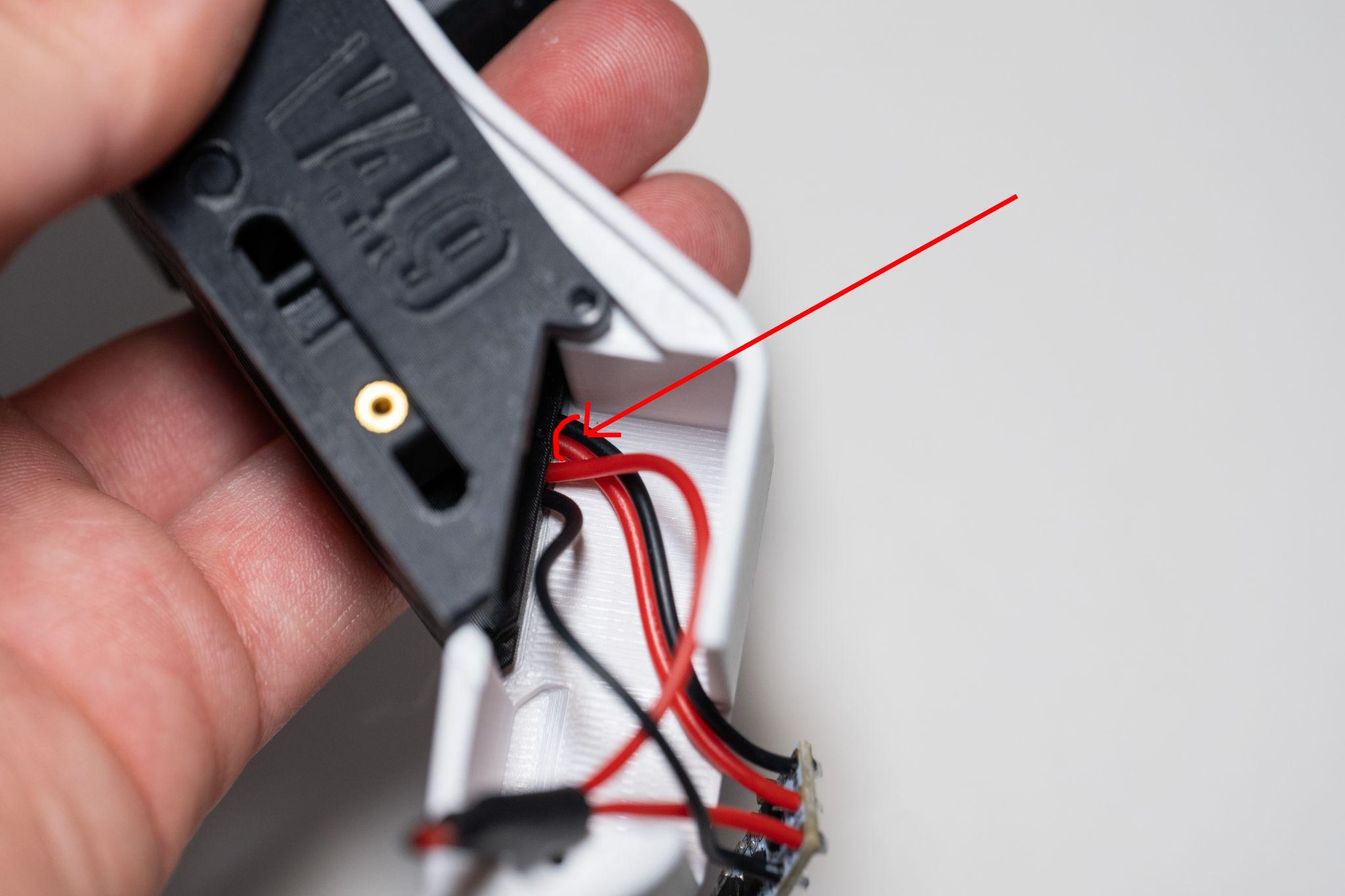

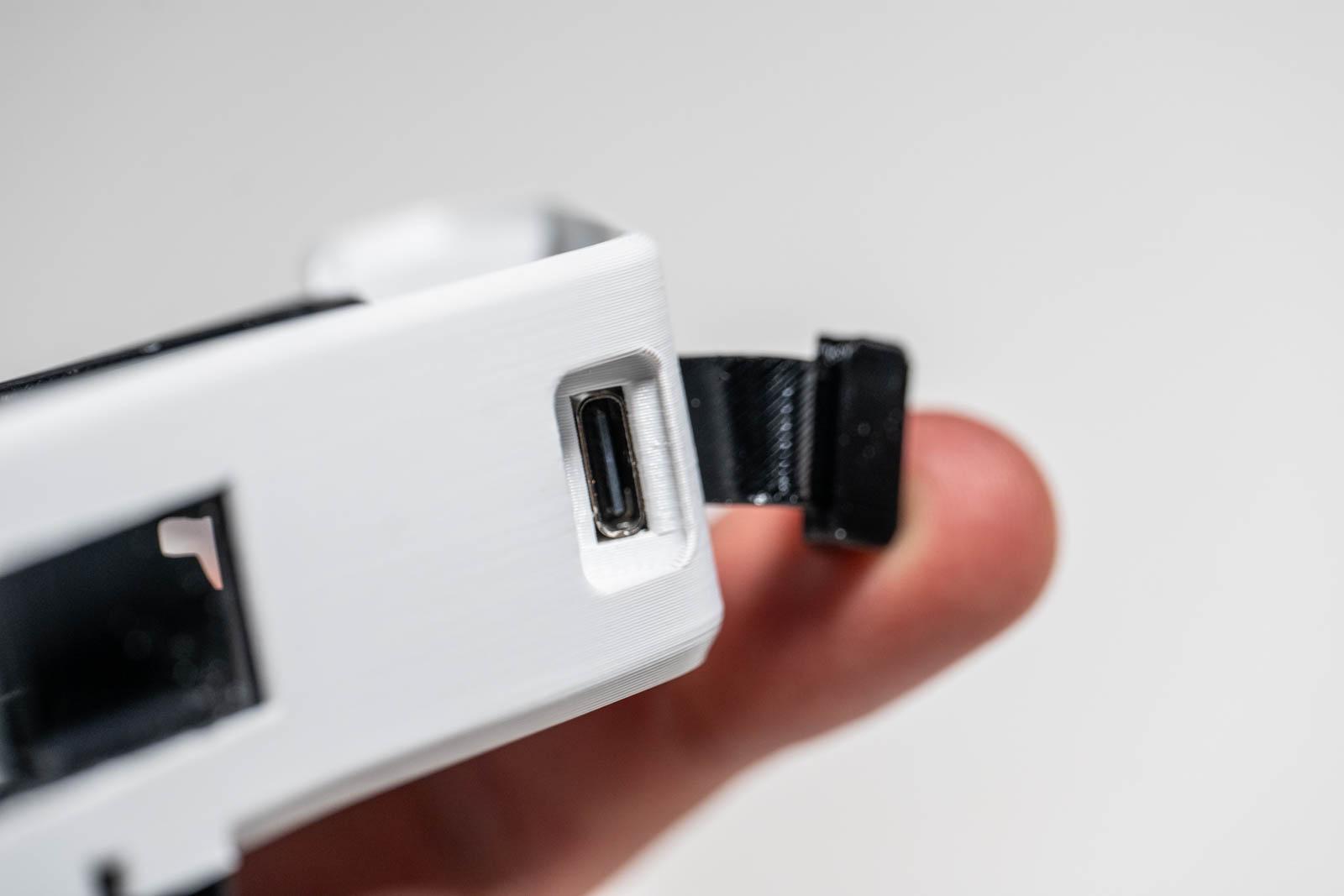


**Figure 48: USB-C flush with frame Figure 49: Pull these two wires**

- Install L bracket as shown in Figure 50. Note the reduced slack between the charge board and core.


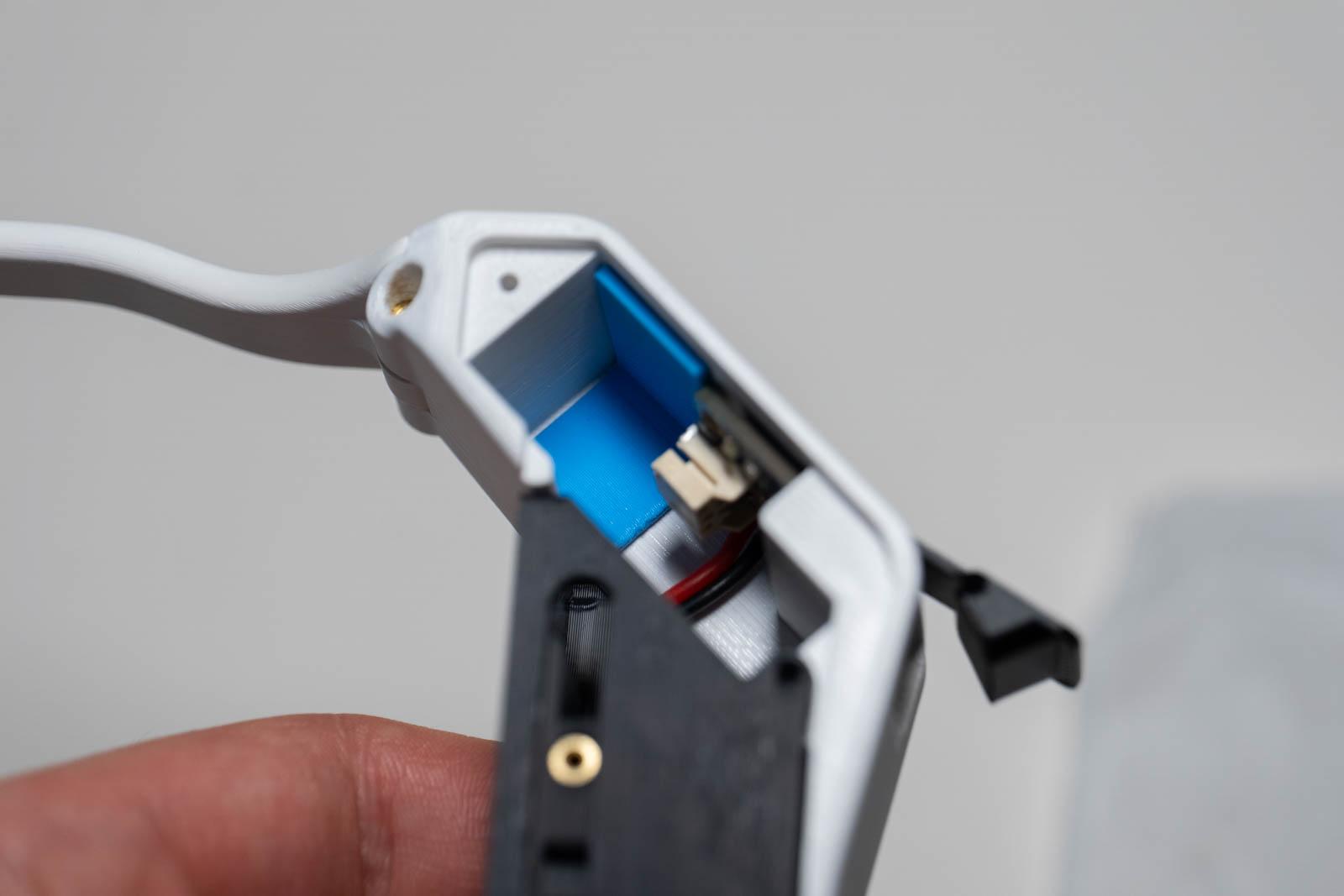


**Figure 50: L bracket position**

##

## Battery install

- Use tweezer pliers to connect the battery to the charge board (Figure 51).
- Rotate battery into place as shown in Figure 52.


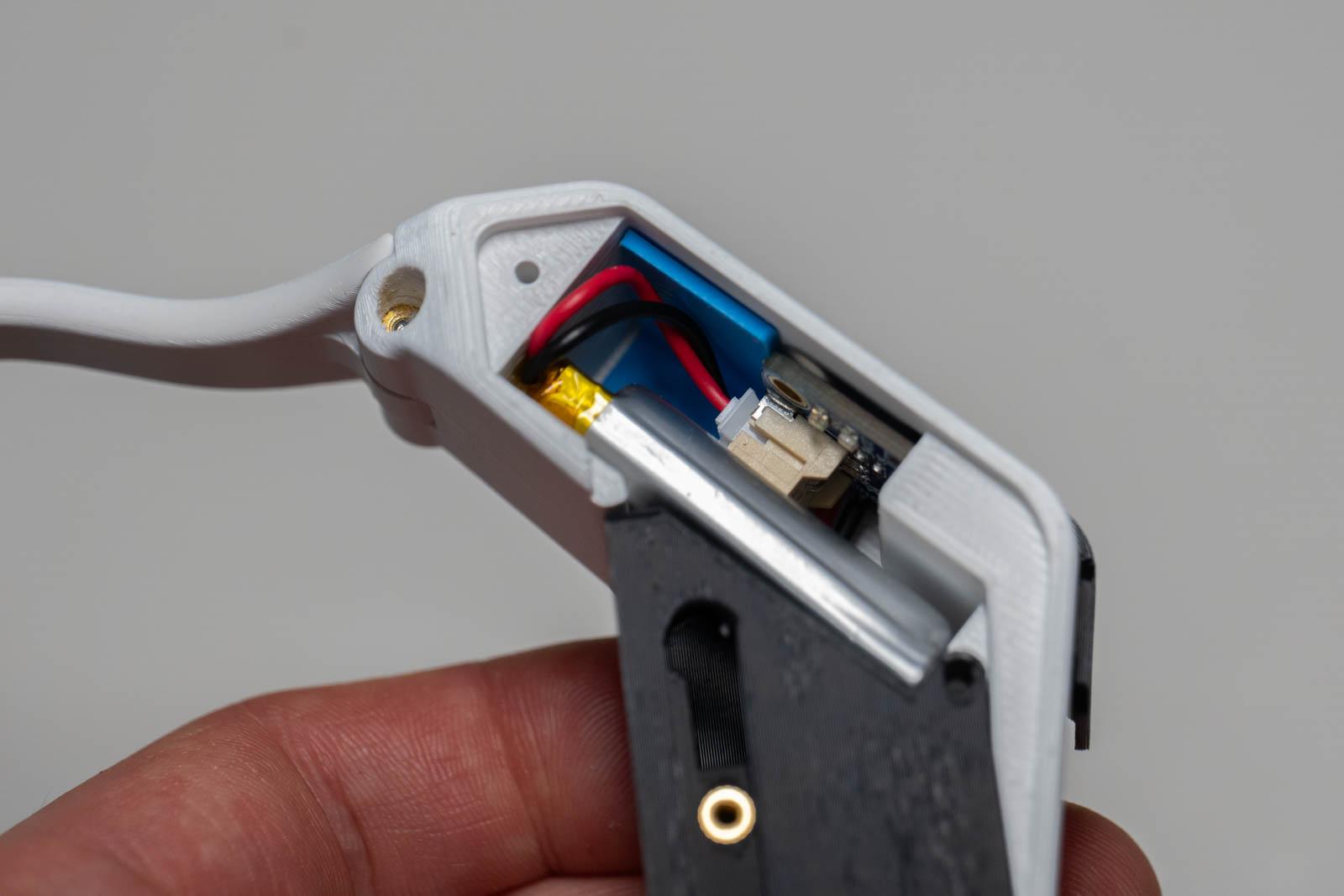

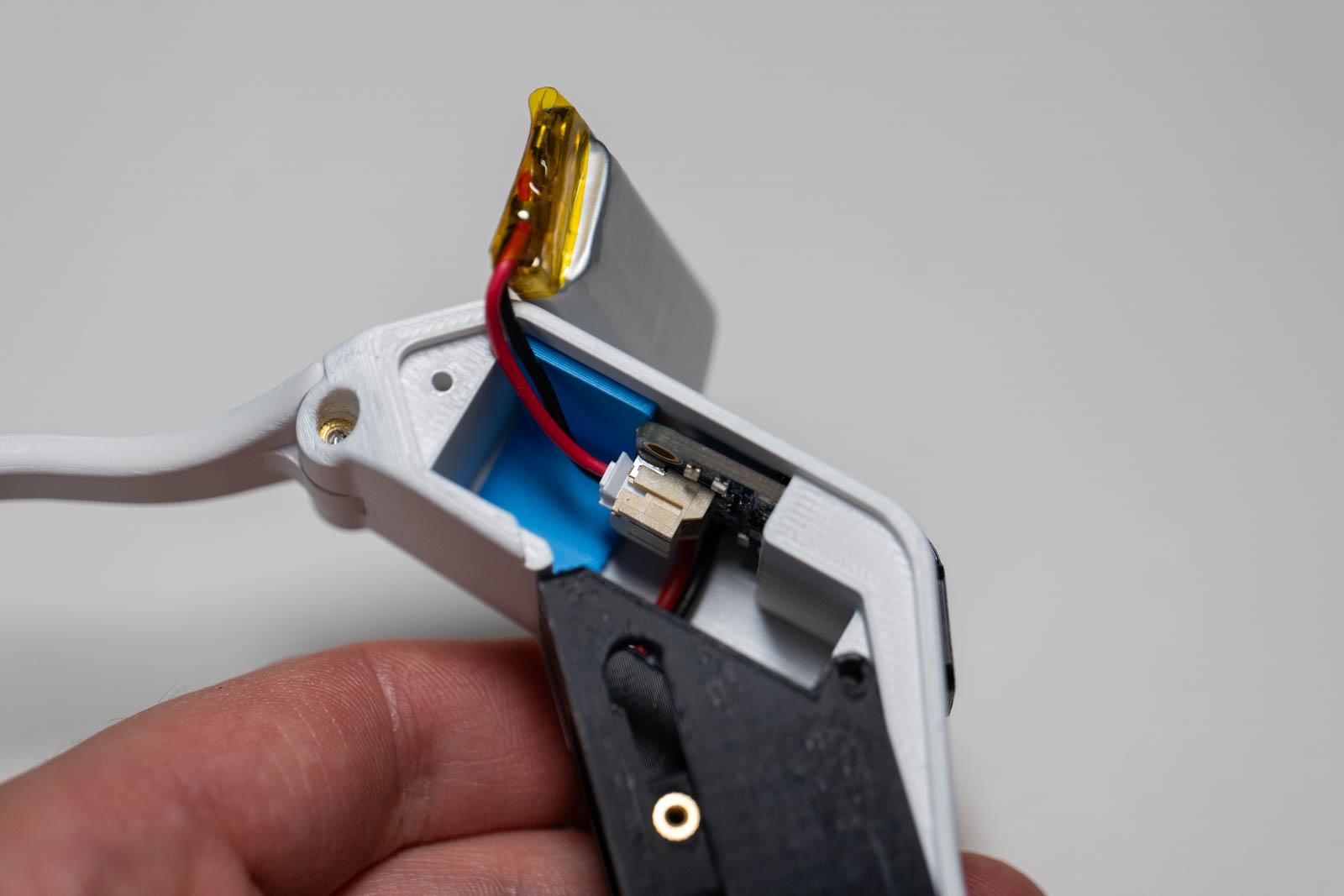


**Figure 51: Connect battery Figure 52: Battery installed**

## PWM install

- Install PWM into printed PWM piece. Fit heatshrink covered resistor between PWM and printed piece as shown in Figure 53.
- On opposite side of printed piece, install washer shiny side facing in, then screw on nut.
- Place PWM piece into frame (Figure 54). Make sure no wires are pinched between printed pieces.


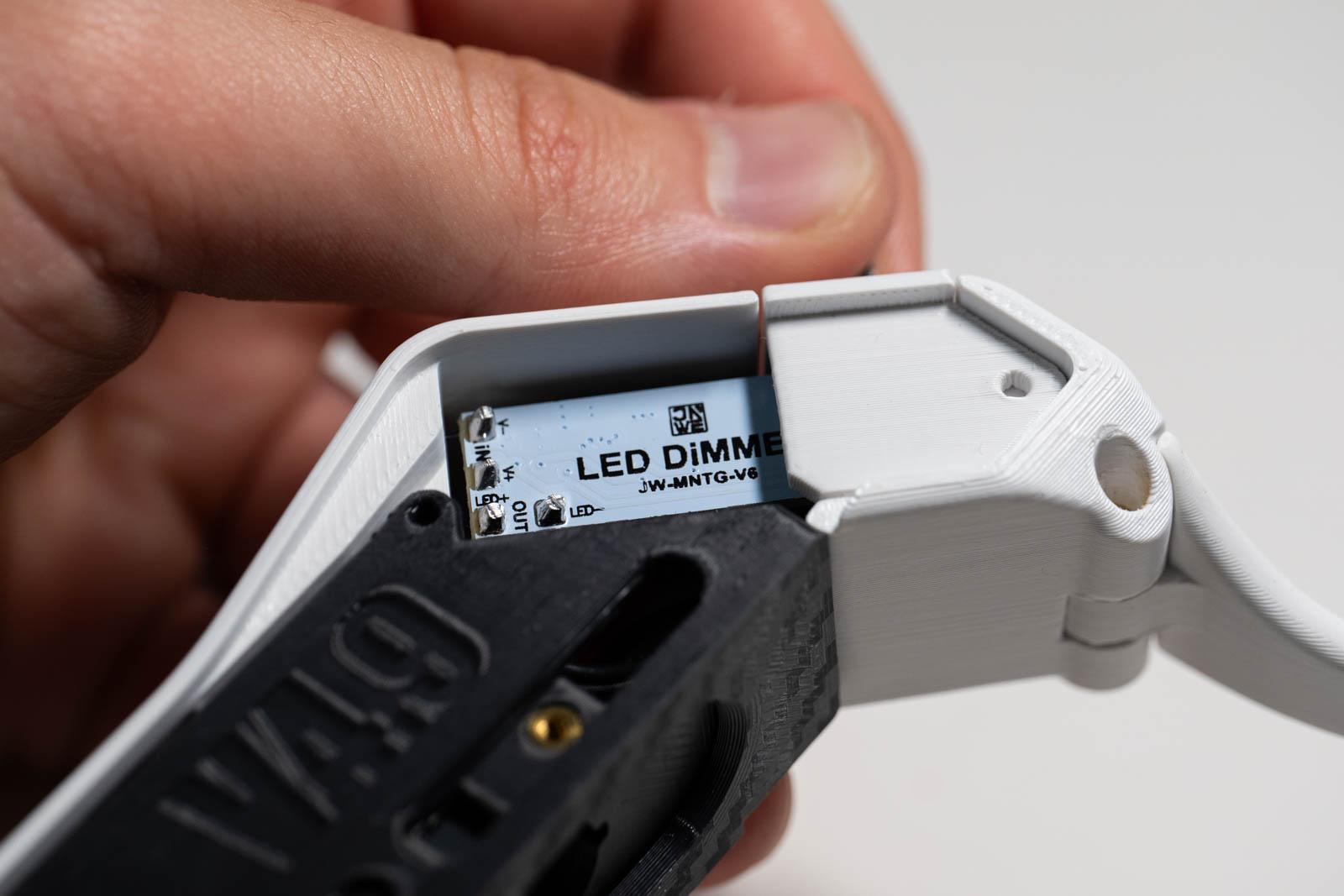

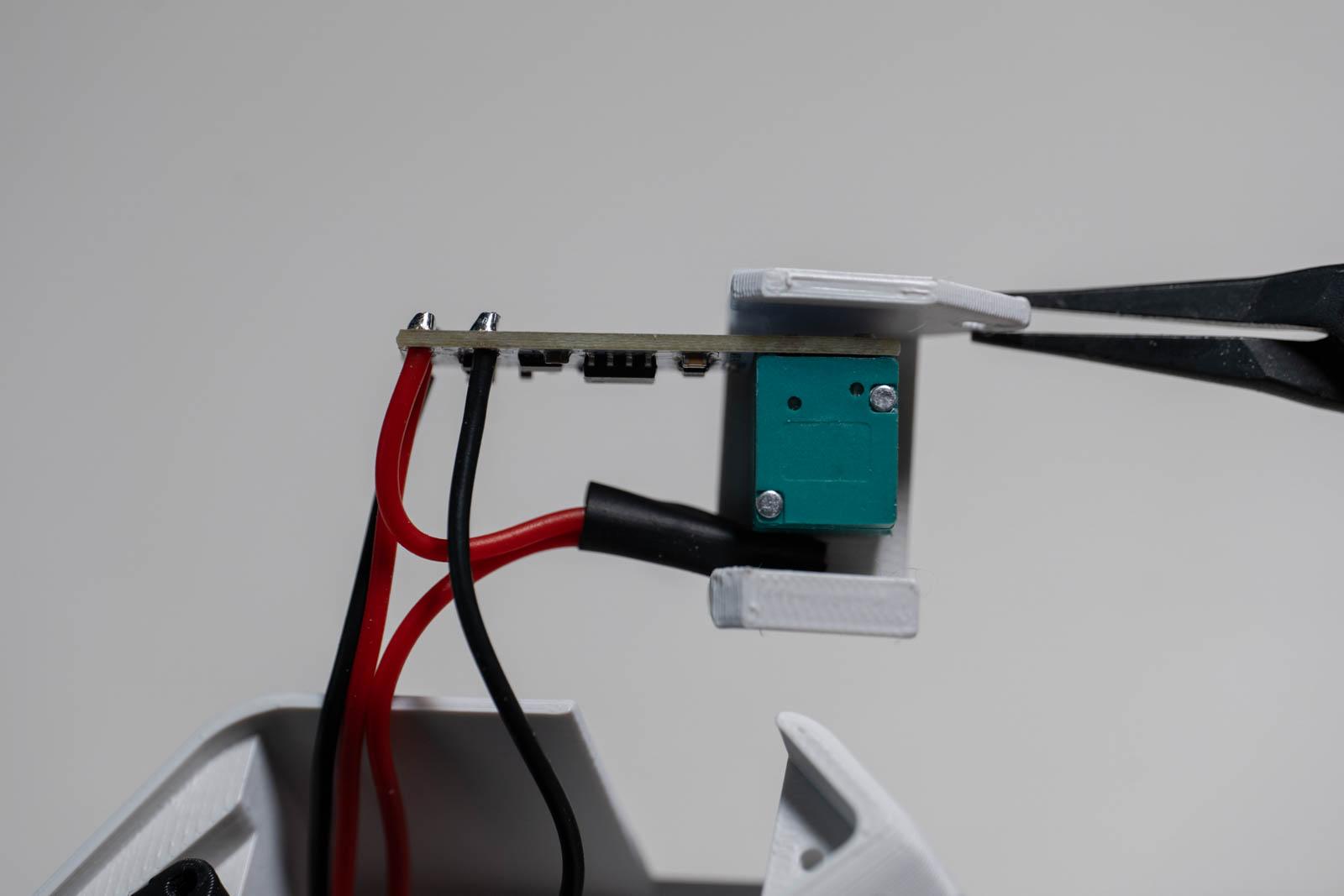


**Figure 53: PWM attached to printed piece Figure 54: PWM installed in frame**

##

## Base install

- Install base piece onto core and frame with 4x M2 10 mm screws (Figure 56). Instead of using 4x M2 10 mm screws, it is also possible to use 2x M2 10 mm screws anterior, and an M2 6 mm screw for the battery side hole and an M2 8 mm screw for the PWM side hole.


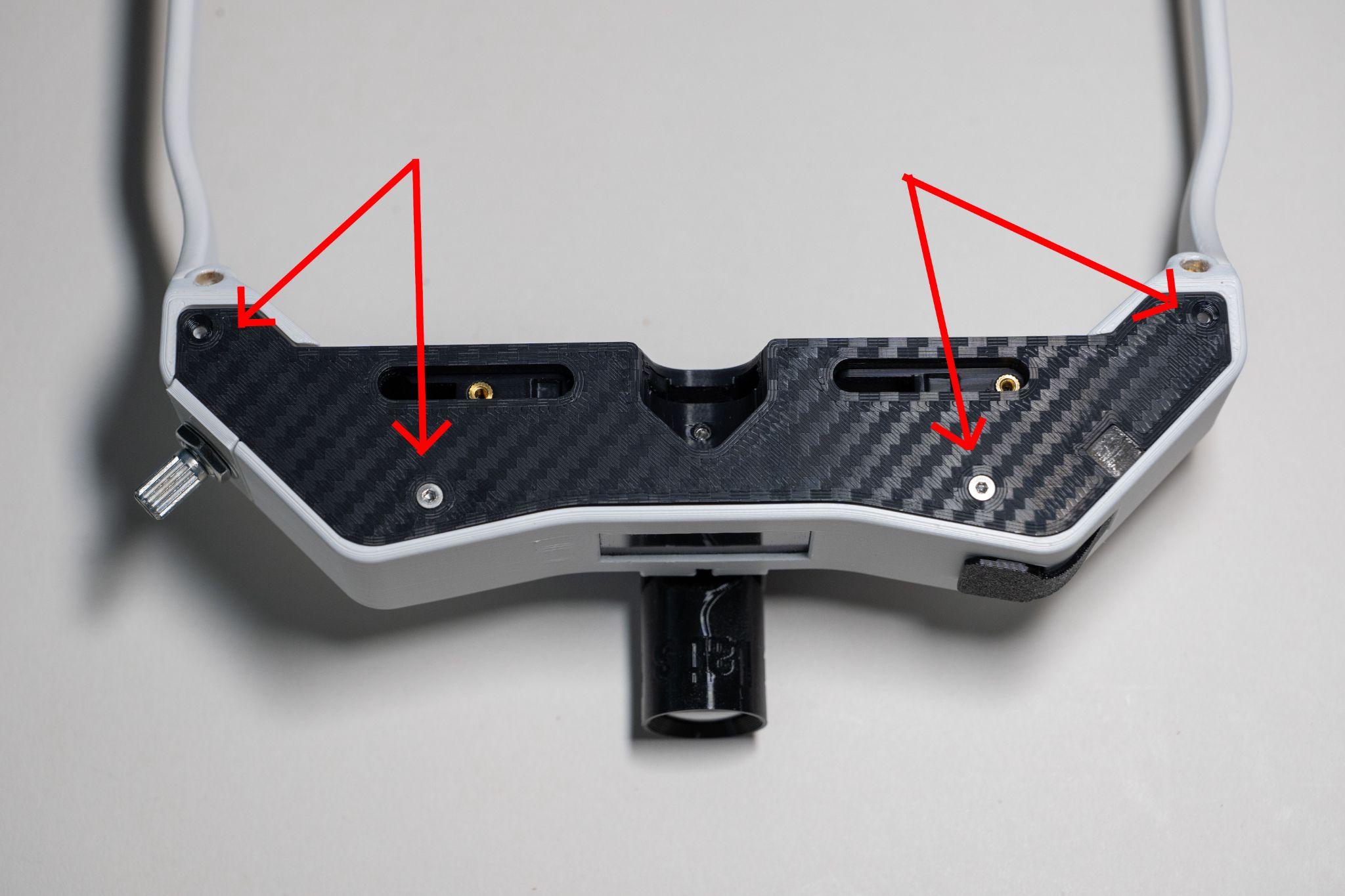

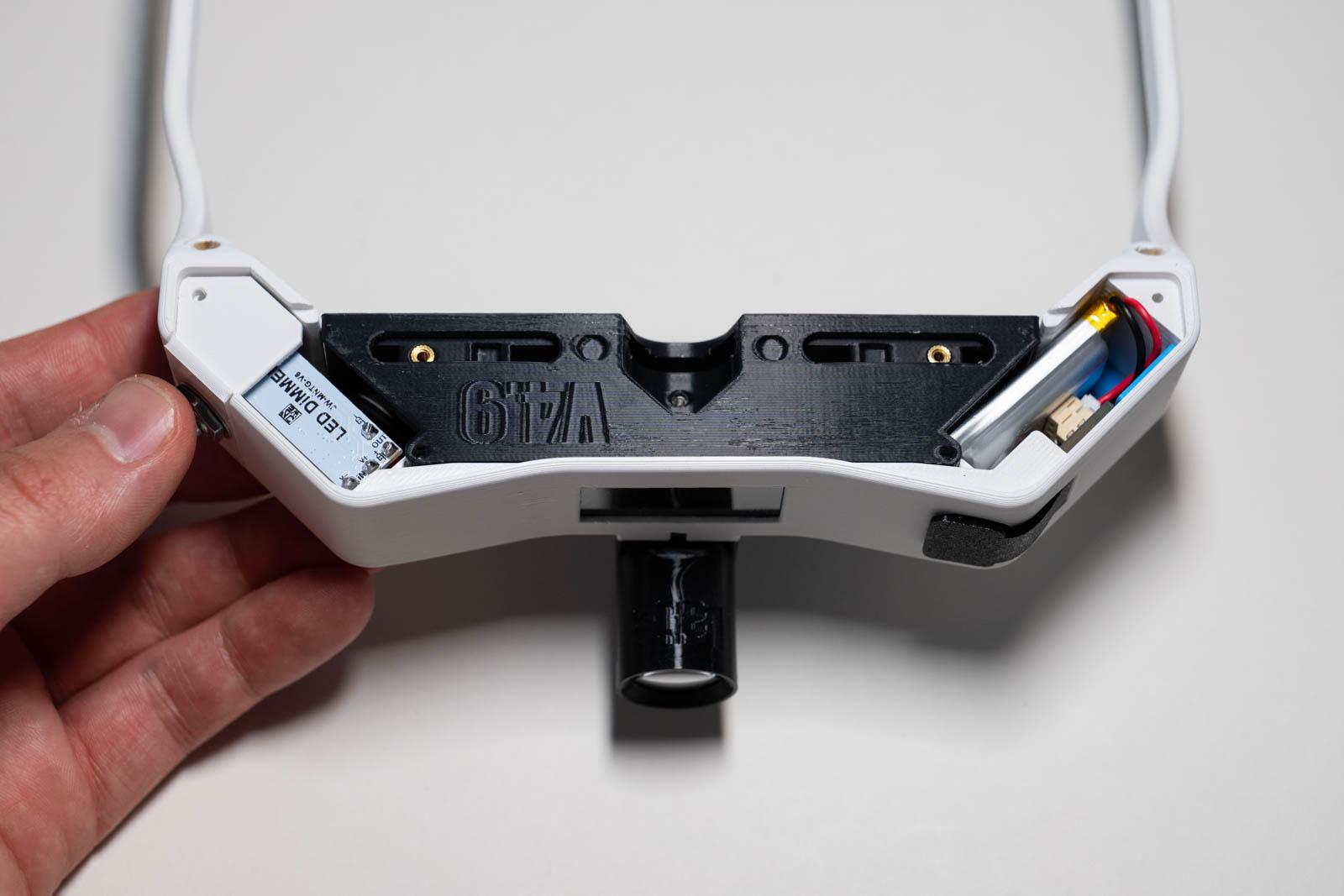


**Figure 55: Frame before base install Figure 56: Base screw points**

- Install PD sliders with 2x M2 10 mm screws one for each slider (Figure 57).
- Install PWM knob with line at 6:30 or 7 o’clock position when device is off (Figure 58).


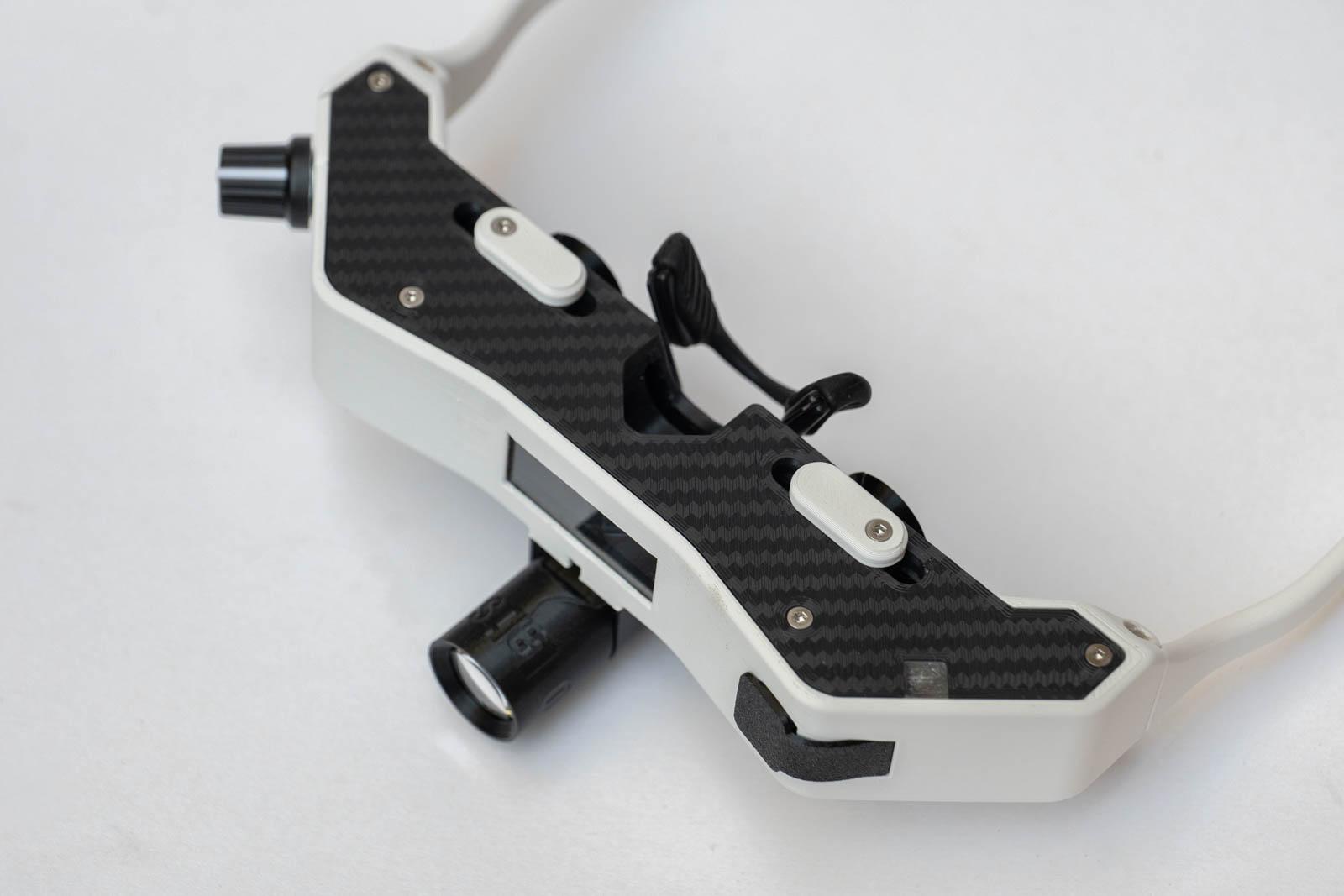

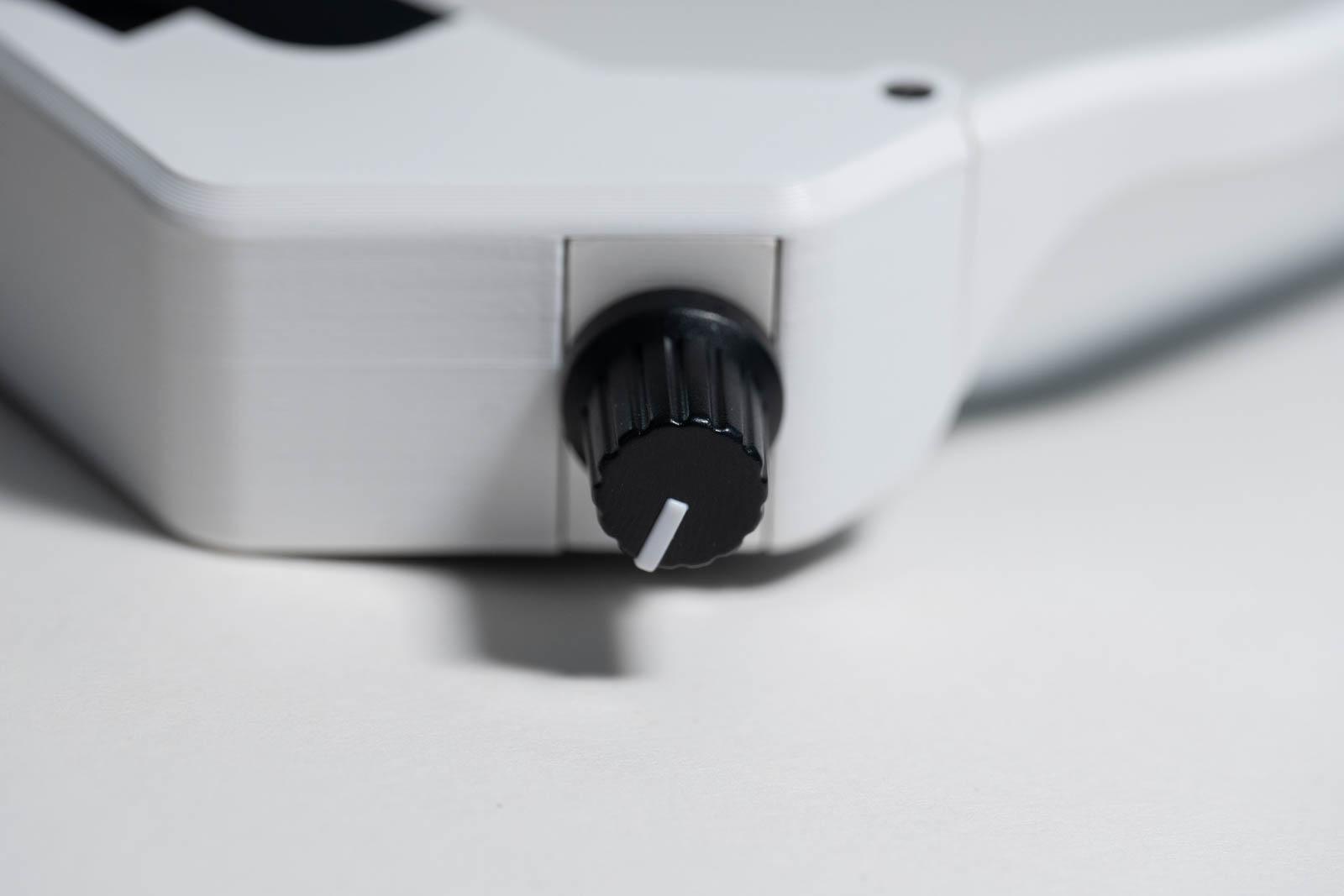


**Figure 57: PD Sliders installed Figure 58: PWM knob position when off**

##

## Nosepiece install

Screw in nosepiece using included screws. Note order: screws, nosepiece, TPU washer, core (Figure 59). Do not overtighten these screws, as they are self-tapping.


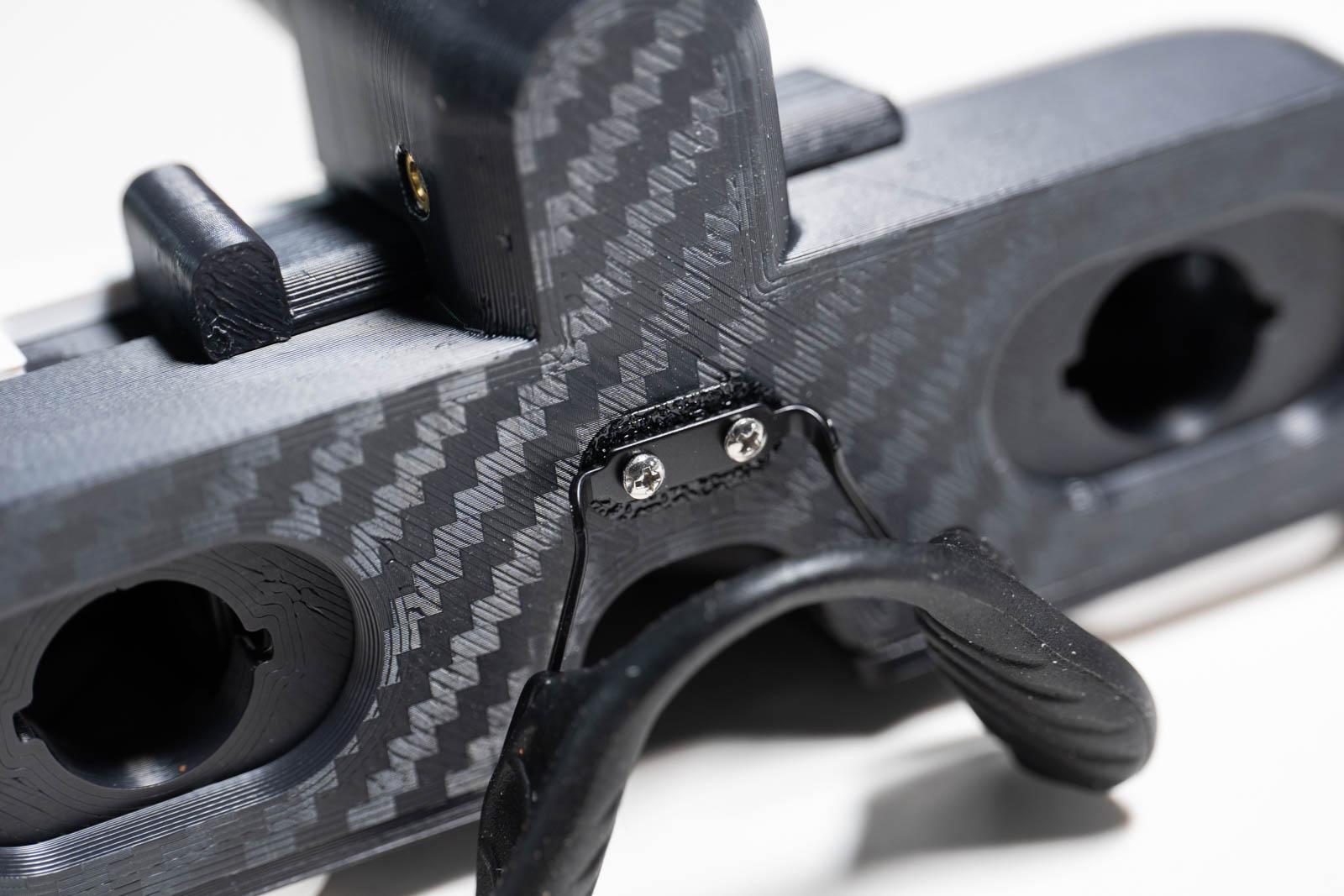


**Figure 59: Nosepiece installed**

## Eyepiece install (2x)

- Insert 12.7 mm lens into each TPU eyepiece. A biconvex lens with a focal length of 500 mm (+2 D) will provide the correct power for an emmetropic user holding a condensing lens at arm’s length. A different power lens can be used based on the user’s refractive error.
- Install TPU eyepiece into indirect by lining up tabs, pressing in, then turning clockwise about 1 clock hour to lock in the eyepiece (Figures 60 and 61). Eyepieces can be easily removed by reversing the previous steps.


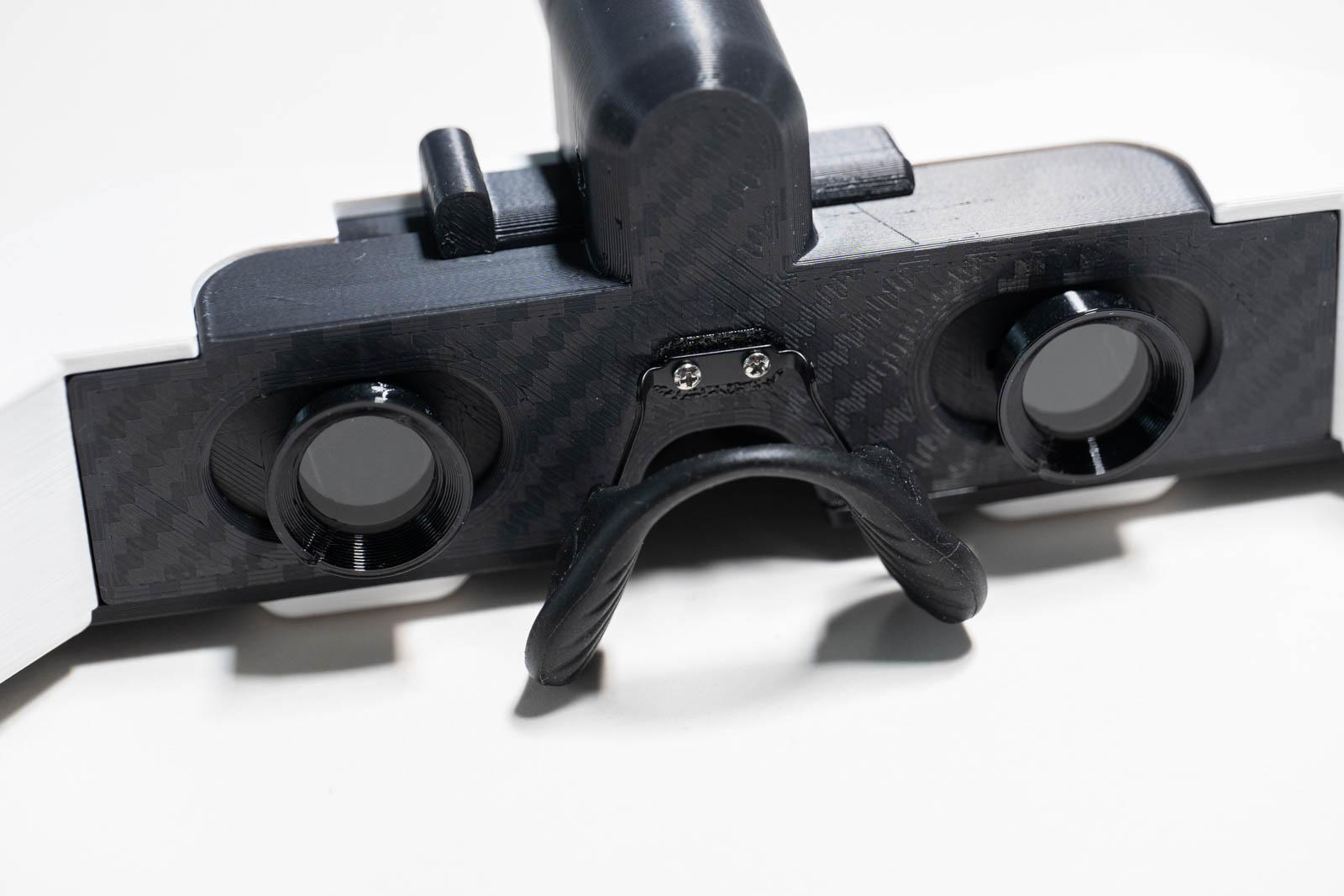

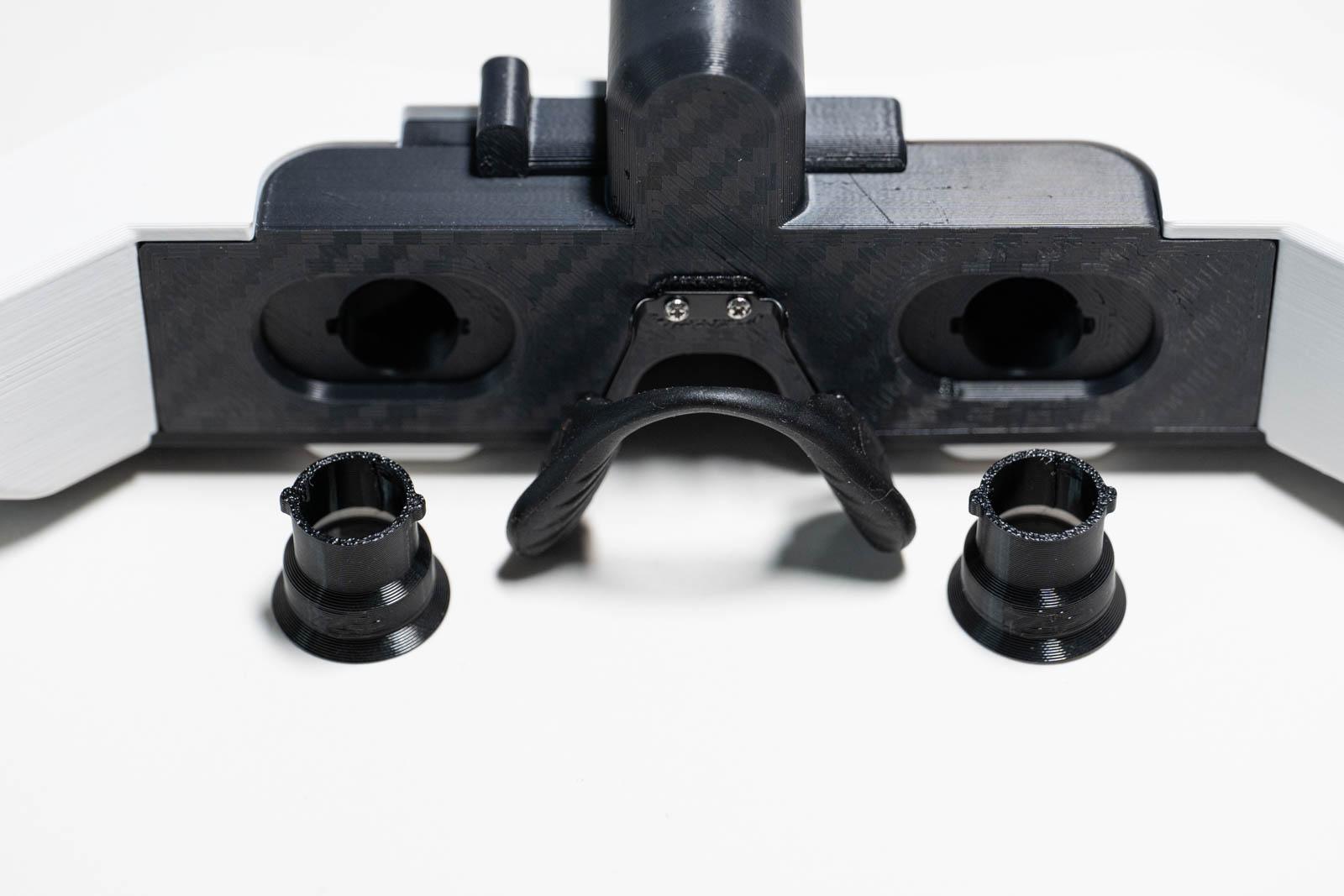


**Figure 60: Eyepieces with tabs aligned Figure 61: Eyepieces installed**

## Glasses retainer

Attach glasses retainer to temples and you are finished!

# Congratulations!

You have just completed building your own compact binocular indirect ophthalmoscope!


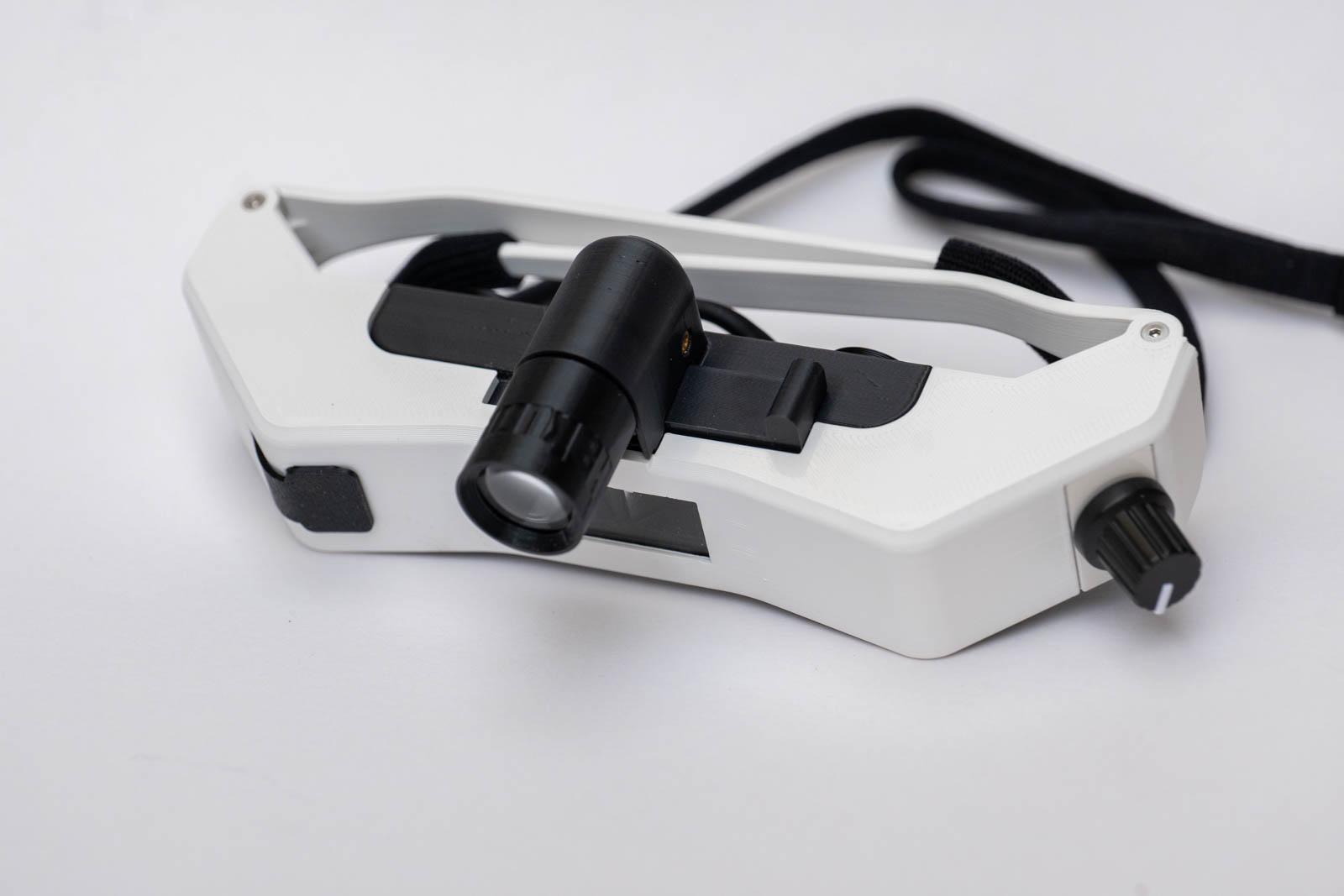


# Accessories

## Blue light filter

- Mark blue gel filter sheet using printed template.
- Use Lexan scissors to cut out gel filter.
- Remove protective film from both sides of gel filter.
- Press gel filter into TPU blue light filter piece.

Troubleshooting

Vertical diplopia

If there is vertical diplopia when using the BIO, it is most likely due to misalignment of the left and right side mirrors and the central mirror. One can test the vertical alignment of the mirrors by turning the indirect around and looking at a straight horizontal line off in the distance through the central mirrors, or (more objectively) by using two lasers as seen in Figure 62.


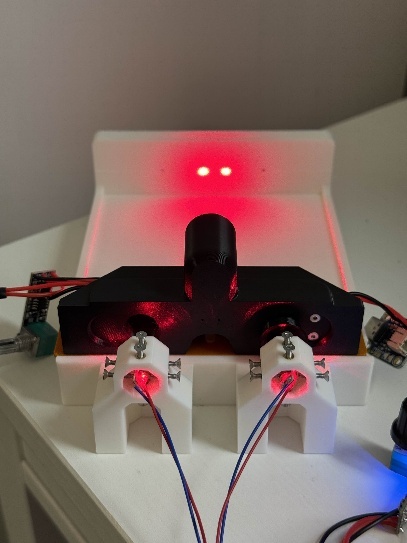


**Figure 62: Testing alignment of the mirrors with two lasers**

If there is significant vertical diplopia, one can print multiple side mirrors and, through trial-and-error, find two side mirrors that match each other so there is minimal vertical misalignment. Alternatively, one can print a single adjustable side mirror, which consists of a compressible TPU gasket sandwiched between two rigid ASA parts (Figure 63 and 64). The ASA part that holds the actual mirror has two heat press inserts temporally, and the ASA part that slides in the core has one heat press insert nasally. The sandwich is held together by an M2 6 mm screw for the nasal heat press insert and two M2 8 mm screws for the temporal heat press inserts (Figure 65). The alignment of the side mirror can then be adjusted by tightening or loosening the three screws, two of which are accessible after assembly of the BIO (Figure 66). The Fusion 360 files for the adjustable side mirror are available as supplemental material.


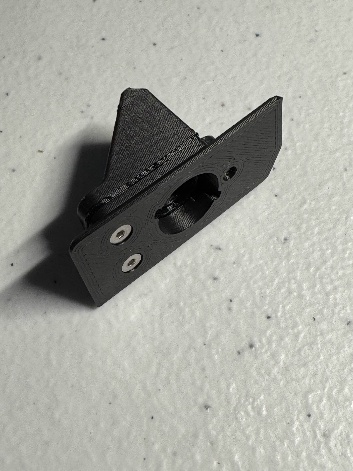

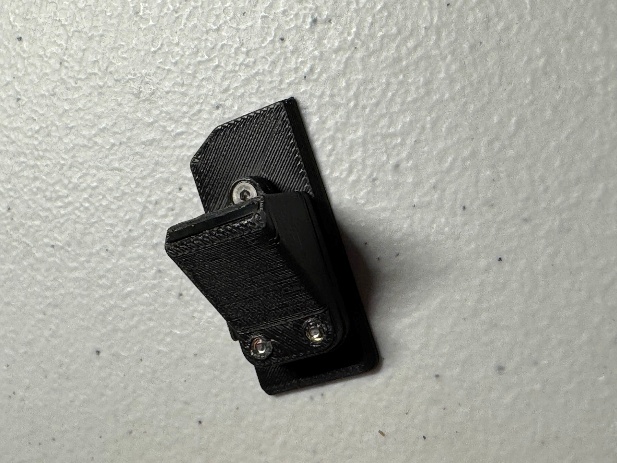


**Figure 63: Adjustable side mirror Figure 64: Adjustable side mirror**

**
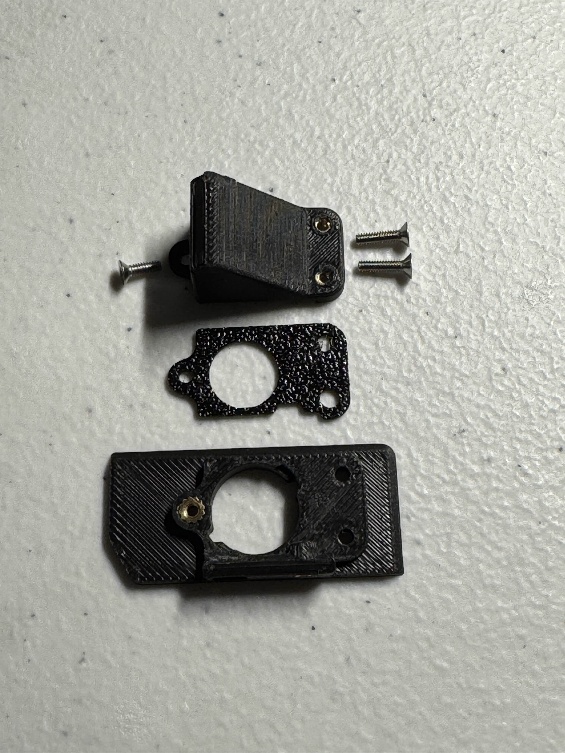

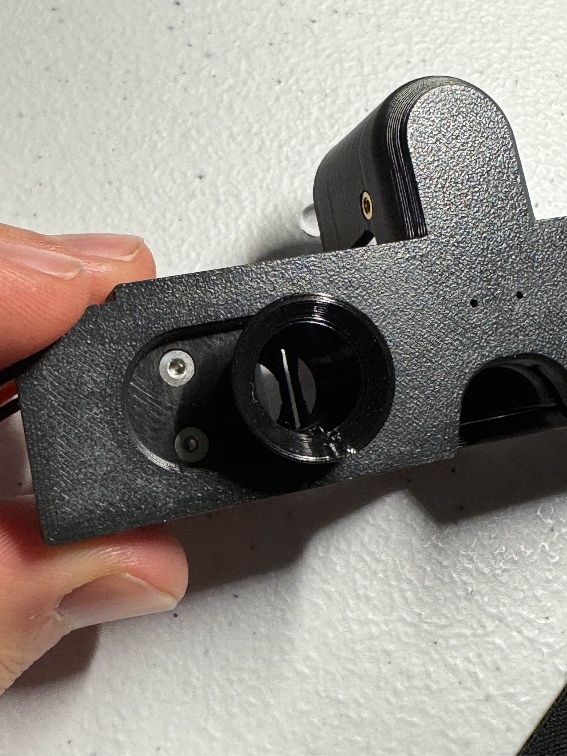
**

**Figure 65: Parts of the adjustable side mirror Figure 66: Adjustable screws**


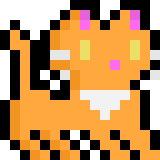

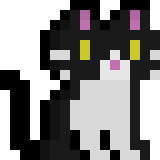

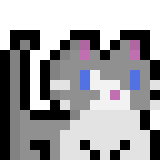

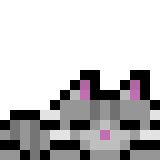

Supplement: Supporting Information — Additional supporting information can be found online in the Supporting Information section. [file 5638606.f1.zip › Build Guide.docx]
