# Supplementary material for: Low-Cost 3D-Printed Binocular Indirect Ophthalmoscope
Source: J Ophthalmol. 2025 Apr 17;2025:5638606. doi: 10.1155/joph/5638606 (PMC12021479; doi:10.1155/joph/5638606)
Supplement: Supporting Information — Additional supporting information can be found online in the Supporting Information section. [file 5638606.f1.zip › Table 1.docx]

**Components**

|  | **Component** | **Brand** | **Part #** | **Cost (USD)** |
| --- | --- | --- | --- | --- |
| ***Materials*** | ASA 1.75 mm filament | Polymaker |  | 2.33 |
|  | Transparent PETG 1.75 mm filament | Overture |  | 0.02 |
|  | TPU 1.75 mm filament  Polycarbonate sheet  M2.5 10 mm set screw  5x M2 x 4 x 3.5 inserts | Overture  Zonon  McMaster-Carr  HELIFOUNER |  | 0.17  0.02  0.36  0.40 |
|  | 3x M2 x 18 mm screws | McMaster-Carr |  | 0.33 |
|  | 6x M2 x 10 mm screws  Cyanoacrylate glue | McMaster-Carr  GH1200 |  | 1.49  0.04 |
| ***Electronics*** | 26 AWG wire | DAOKI |  | 0.08 |
|  | 8.2 ohm resistor  63/37 solder  Heat shrink tubing | CHANZON  Kester  DHOOZ |  | 0.05  0.02  0.02 |
|  | Micro-Lipo charger USB Type C | Adafruit | 4410 | 5.95 |
|  | 5 mm light-emitting diode | QT Brightek | QBL8IW15C-WW | 0.44 |
|  | 400 mAh 3.7 V battery | Adafruit | 3898 | 6.95 |
|  | PWM dimmer | ICStation | GY20784-1 | 3.91 |
| ***Optics*** | 4x 12.7 x 18 mm mirrors | Edmund Optics | 83-535 | 62.00 |
|  | 15 mm Dia. x 30 mm FL planoconvex lens | Edmund Optics | 23-085 | 27.50 |
|  | 2x 12.7mm Dia. x 500 mm FL biconvex lenses | Eksma Optics | 111-0152E | 34.00 |
|  | SK6 diaphragm | ViaGasaFamido |  | 10.28 |
| ***Accessories*** | Glasses strap | SHINKODA |  | 0.90 |
|  | Ergo Fit Nose Pad | Surgitel | 26905-1-3-8 | 25.00 |
|  |  | **Total** |  | **182.26** |

**Tools**

| **Tool** | **Brand** |  | **Cost (USD)** |
| --- | --- | --- | --- |
| X1-Carbon 3D Printer with AMS  File set  Deburring tool  X-ACTO knife #1  Micro hand drill  Hexagon screwdriver 1.3 mm  Slotted and Philips screwdriver set  Heat press insert tip M2  Tweezer nose pliers  Lexan scissors  Wire cutter and stripper  Air blower  FX888D-23BY digital soldering station  Lighter | Bambu Lab  Mudder  AFA Tooling  X-ACTO  Craft911  Wera  Wiha  Virtjoule  Xuron  Hobbypark  DOWELL  JJC  Hakko  BIC |  | 1449  2.66  14.99  5.99  12.99  8.32  25.45  8.95  17.03  9.97  6.99  7.99  133.62  3.25 |
|  | **Total** |  | **1707.20** |
